# Supplementary material for: Domain-inlaid Nme2Cas9 adenine base editors with improved activity and targeting scope
Source: Nat Commun. 2024 Feb 17;15:1458. doi: 10.1038/s41467-024-45763-5 (PMC10874451; doi:10.1038/s41467-024-45763-5)
Supplement: Supplementary file 1 — Supplementary Information [file 41467_2024_45763_MOESM1_ESM.pdf]

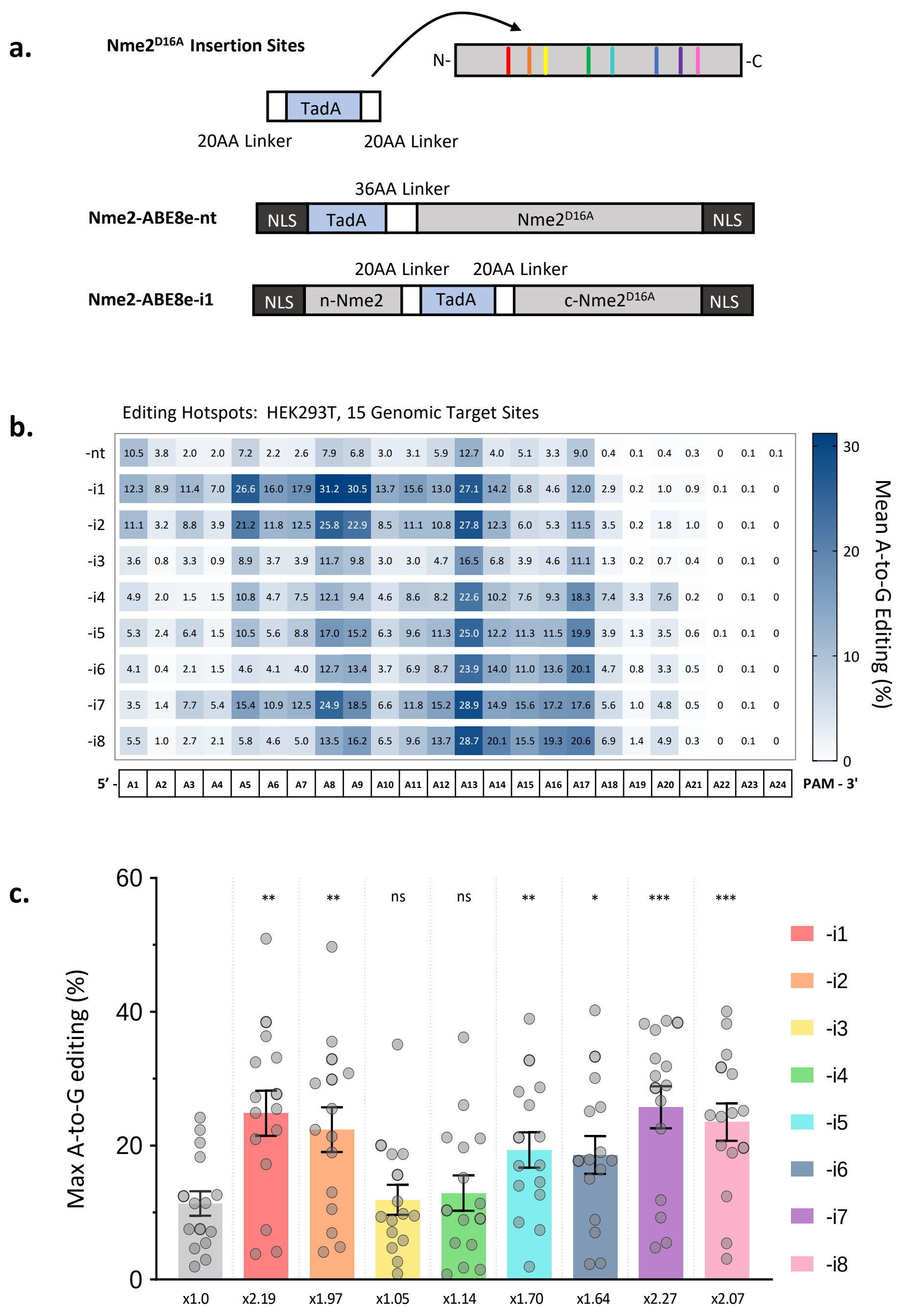

Supplementary Figure 1. Design of Domain-inlaid Nme2-ABEs. **a)** Schematic representation of Nme2Cas9-ABE8e constructs and sites of deaminase domain insertion. **b)** Summary of mean A-to-G editing activities and editing windows for the Nme2-ABE8e constructs in HEK293T cells. Numbers provided for each position in the protospacer represent the mean A-to-G editing efficiency across 15 endogenous target sites in HEK293T cells measured via amplicon deep sequencing (n = 3 biological replicates). **c)** Data from (B) were aggregated and replotted, with each data point representing the maximum A-to-G editing efficiency of an individual target site, as measured by amplicon deep sequencing (n = 3 biological replicates; data represent mean ± SEM). One-way ANOVA analysis: ns, $p > 0.05$; $^{**}p < 0.05$; $^{***}p < 0.001$ $^{****}p < 0.0001$.

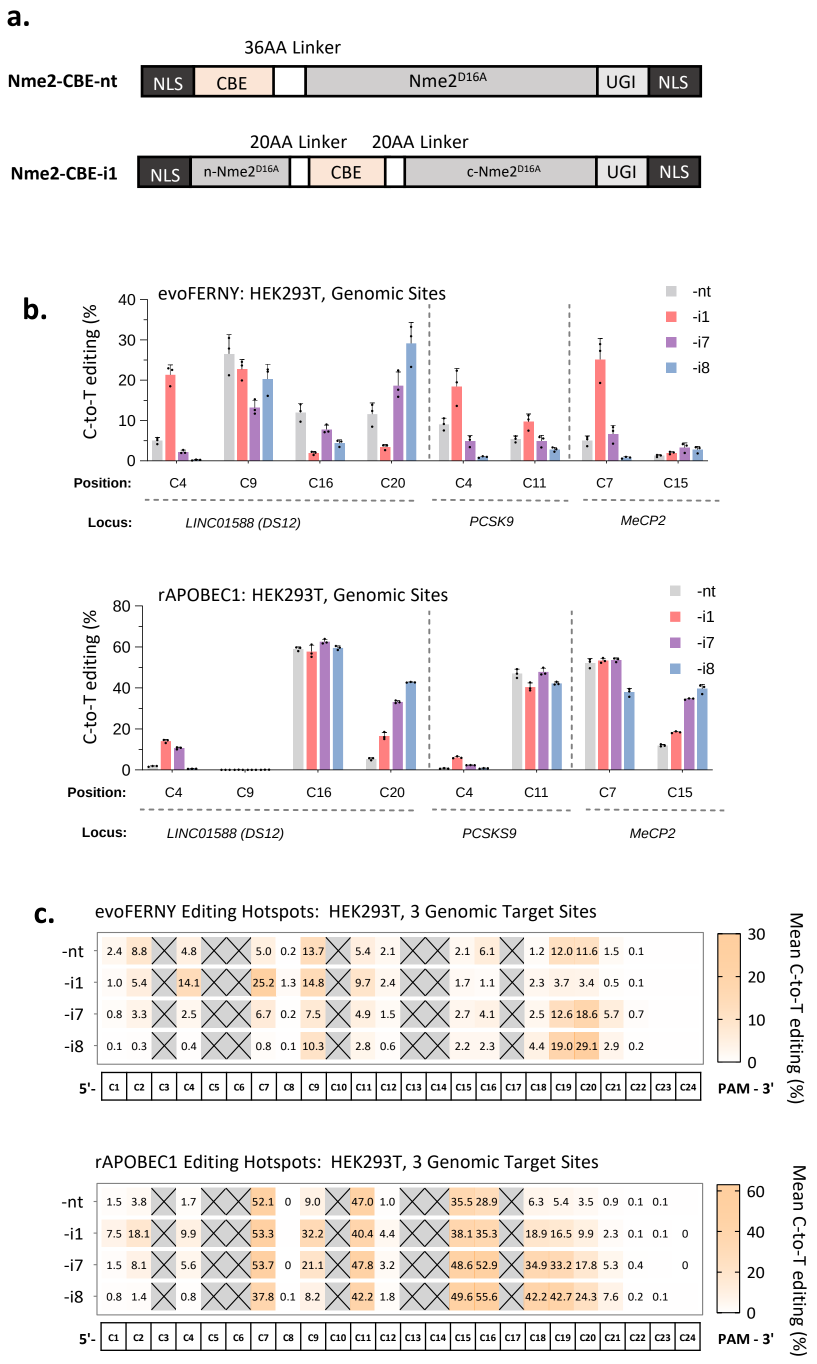

Supplementary Figure 2. Editing with the CBEs rAPOBEC1 and evoFERNY. a) Schematic representation of Nme2Cas9-CBE constructs with evoFERNY or rAPOBEC1 deaminases. **b)** Exemplary C-to-T editing at endogenous HEK293T genomic loci with Nme2-evoFERNY (top) or Nme2-rAPOBEC1 (bottom) constructs, measured by amplicon deep sequencing. (n = 3 biological replicates per off-target R-loop; data represent mean ± SD). **c)** Summary of Mean C-to-T editing at the three endogenous HEK293T genomic loci with Nme2-evoFERNY (top) or Nme2-rAPOBEC1 (bottom) constructs. Crossed out boxes denote no cytidine at the position within the target's tested. Measured via amplicon sequencing. n = 3 biological replicates.

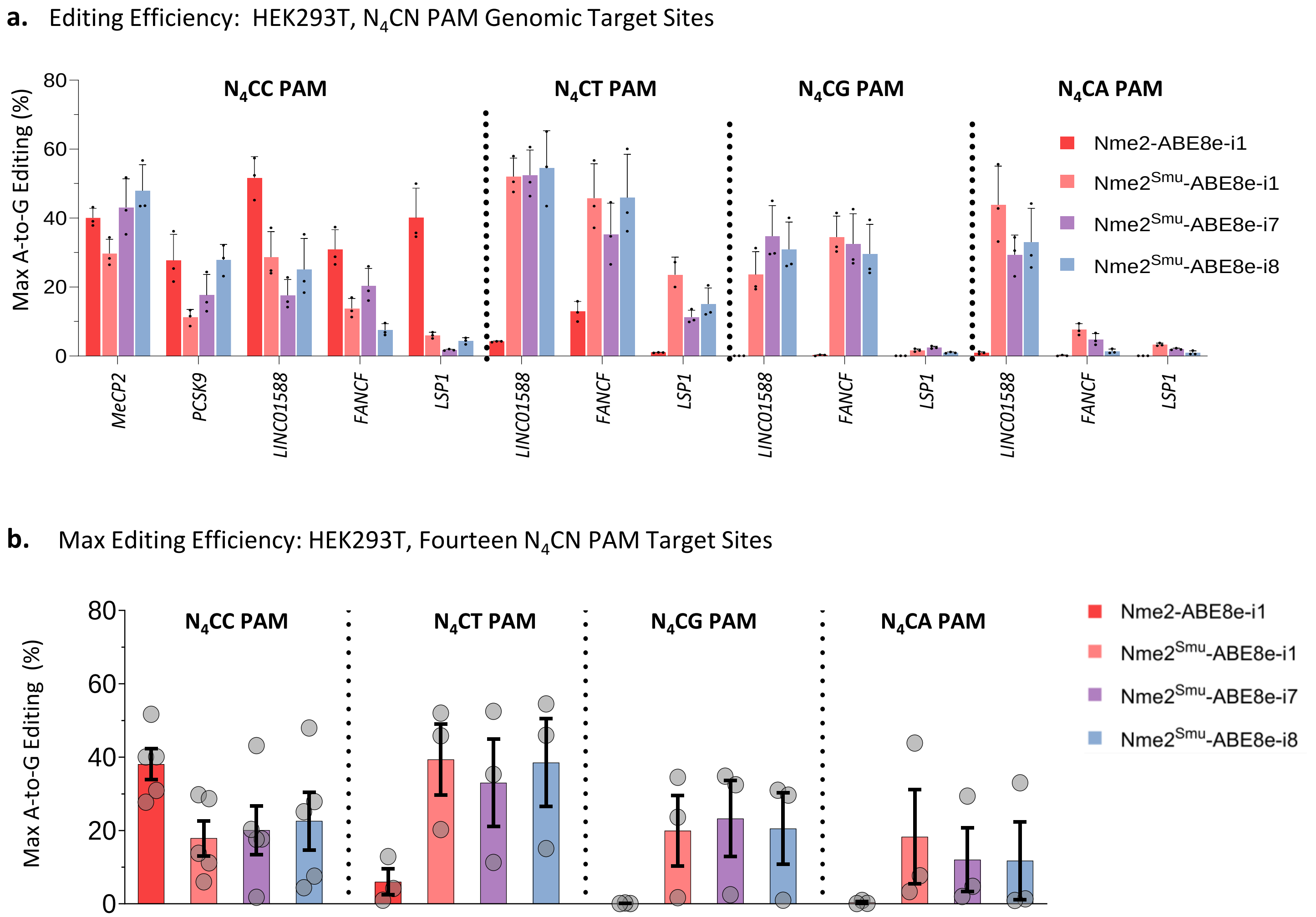

Supplementary Figure 3. PAM-Interacting Domain Chimeras Expand the Targeting Scope of Nme2Cas9 Effectors. **a)** A-to-G editing following transfection with WT or chimeric PID Nme2-ABE8e effectors at endogenous HEK293T genomic loci with N4CN PAMs by plasmid transfection. The editing efficiency at the maximally edited adenine for each target was plotted. Editing activities were measured by amplicon sequencing (n = 3 biological replicates; data represent mean ± SD). **b)** Data from (a) were aggregated and replotted, with each data point representing the maximum A-to-G editing efficiency of an individual target site, as measured by amplicon deep sequencing (n = 3 biological replicates; data represent mean ± SEM).

a.

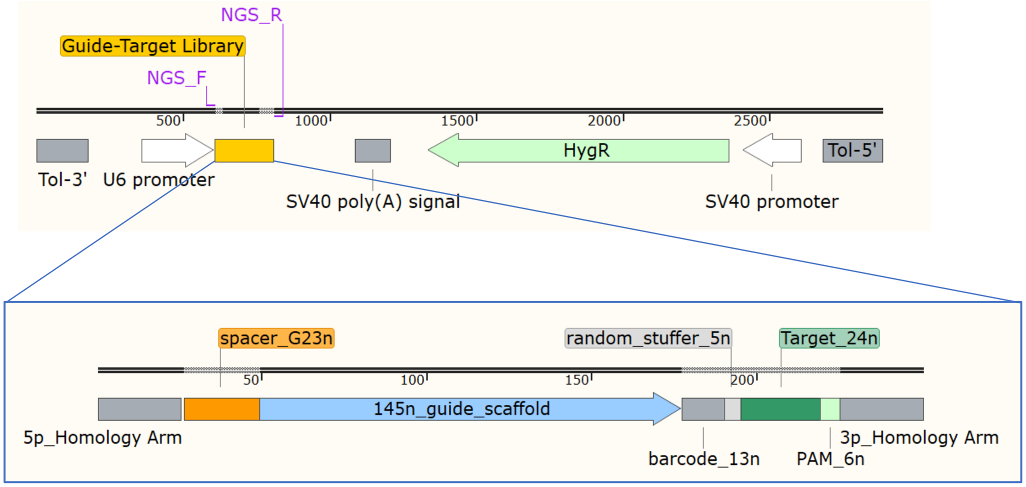

b.

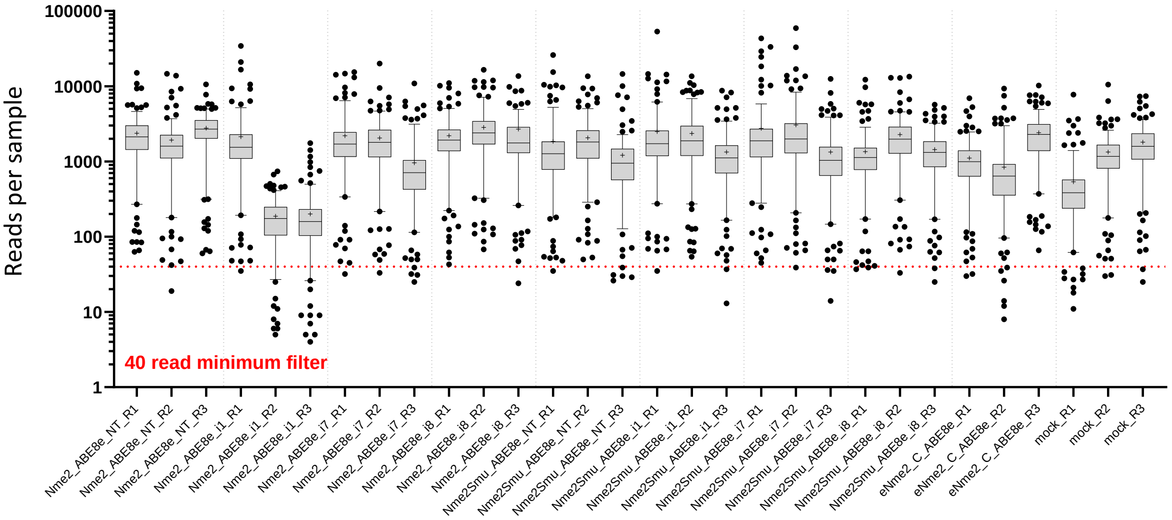

c.

	Pearson *r*		
**Sample**	**Rep1 vs 2**	**Rep1 vs 3**	**Rep2 vs 3**
Nme2-ABE8e-NT	0.88	0.92	0.87
Nme2-ABE8e-i1	0.76	0.81	0.70
Nme2-ABE8e-i7	0.79	0.82	0.87
Nme2-ABE8e-i8	0.89	0.91	0.92
Nme2Smu-ABE8e-NT	0.81	0.73	0.81
Nme2Smu-ABE8e-i1	0.73	0.63	0.68
Nme2Smu-ABE8e-i7	0.82	0.82	0.79
Nme2Smu-ABE8e-i8	0.86	0.86	0.88
eNme2-C-ABE8e	0.83	0.83	0.75

d.

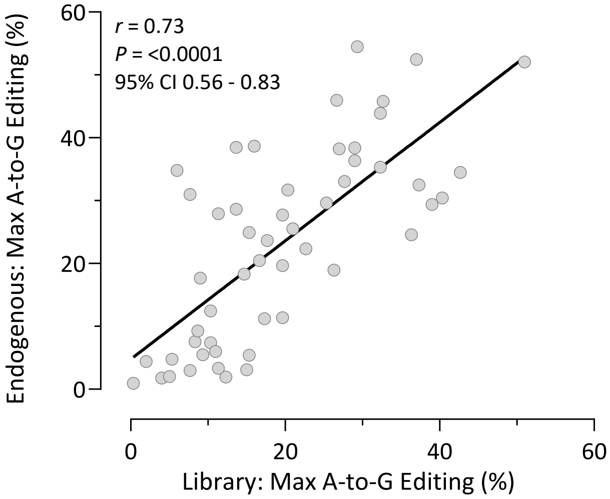

Supplementary Figure 4. Guide-Target Library Quality Control. a) Cartoon depicting guide-target library configuration. **b)** Number of reads per guide-target library member. Boxplots represent median and interquartile ranges; whiskers indicate 5th and 95th percentiles with the cross indicating the mean. **c)** Pearson correlation between biological replicates (Rep1, 2 or 3), comparing mean editing rates across the adenines of a library member target site from Figure 2b. See Supplementary Table 1 for additional summary statistics. **d**) Pearson correlation of editing activity between endogenous targets and their matched members within the guide-target library.

a. Editing Window: HEK293T, Integrated Guide-Target Library, N$_4$CN PAM Sites

5'-	1	2	3	4	5	6	7	8	9	10	11	12	13	14	15	16	17	18	19	20	21	22	23	24
Nme2-ABE8e-nt	0.1	0.8	1.2	1.9	2.7	2.5	3.3	1.6	3.8	3.4	1.6	2.7	2.9	1.5	2.1	2.5	2.1	0.6	0.3		0	0.1	0	
Nme2^{Smu}-ABE8e-nt	0.2	1.0	1.8	3.7	3.8	7.8	8.9	6.5	9.1	7.6	6.8	7.7	5.6	6.1	6.3	3.2	3.5	2.4	0.8	0.1		0.1	0	
Nme2-ABE8e-i1	0.5	1.4	3.0	3.9	5.7	5.6	7.7	4.5	7.2	5.2	4.3	3.1	3.6	2.5	3.1	3.1	1.9	0.9	0.3	0.8	2.4	0.1	0	0.1
Nme2^{Smu}-ABE8e-i1	1.2	2.7	8.4	14.9	20.5	23.4	26.2	23.8	22.3	18.7	16.7	15.1	11.7	13.3	12.6	7.9	5.3	4.6	1.1	0.4	0.1	0.1		0.1
Nme2-ABE8e-i7	0	0.3	1.5	1.7	3.3	3.3	3.7	1.7	5.3	3.0	2.4	2.9	3.9	2.0	3.0	4.2	3.6	1.7	0.9	0.1	0.5		0	0
Nme2^{Smu}-ABE8e-i7	0.2	0.5	2.2	5.3	7.1	12.1	14.6	10.1	12.7	11.7	10.4	12.8	9.5	11.0	10.4	9.6	6.8	5.5	2.2	0.5	0.1	0.1	0.1	0.1
Nme2-ABE8e-i8	0.1	0.7	1.1	1.5	2.5	2.4	3.2	2.4	6.0	4.8	4.4	4.5	7.4	3.4	5.3	7.2	5.6	3.0	1.4	0.2	0	0.1	0	0.1
Nme2^{Smu}-ABE8e-i8	0.2	0.5	0.8	1.4	1.6	4.6	7.1	5.9	9.6	9.3	9.7	11.6	8.5	10.0	10.3	7.4	6.8	5.7	3.0	0.5	0.2	0.1	0.1	
eNme2-C	0.2	0.8	2.9	5.2	7.3	7.5	11.8	13.6	14.9	13.0	14.8	13.2	12.8	7.6	4.9	3.4	2.2	1.3	0.4	0.1			0	0.1
mock	0	0	0.2	0.2	0.4	0.5	0.5	0.5	0.5	0.4	0.2	0.5	0.4	0.2	0.3	0.2	0.1	0.4		0	0.1	0.1	0	0

PAM -3'

Mean A-to-G Editing (%) (scale 0–30)

b. Normalized Window: HEK293T, Integrated Guide-Target Library, N$_4$CN PAM Sites

5'-	1	2	3	4	5	6	7	8	9	10	11	12	13	14	15	16	17	18	19	20	21	22	23	24
Nme2^{Smu}-ABE8e-nt	2.2	11.4	19.9	40.4	42.3	85.7	97.8	71.3	100.0	84.2	75.4	85.3	61.4	67.3	69.1	35.3	38.2	26.8	9.2	1.1	0.4	1.5	0	0.4
Nme2^{Smu}-ABE8e-i1	4.6	10.3	31.9	56.9	78.0	89.3	100.0	90.9	84.9	71.3	63.8	57.7	44.6	50.6	47.9	30.2	20.1	17.7	4.1	1.4	0.5	0.3	0.1	0.3
Nme2^{Smu}-ABE8e-i7	1.1	3.2	15.3	36.4	48.5	83.3	100.0	69.3	87.4	80.3	71.6	87.6	65.0	75.3	71.4	65.7	46.7	37.5	15.3	3.2	0.7	0.9	0.5	0.5
Nme2^{Smu}-ABE8e-i8	1.4	4.3	6.9	11.8	13.5	39.8	61.7	50.7	83.0	80.1	84.1	100.0	73.2	86.2	89.0	64.3	58.5	49.6	25.9	4.0	1.7	1.2	0.6	0.3
eNme2-C	1.1	5.6	19.5	34.9	48.8	50.3	79.4	91.5	100.0	87.5	99.3	88.6	86.1	51.2	32.7	22.6	15.0	8.9	2.9	0.7	0.2	0.2	0	0.7

PAM -3'

Normalized Window o (%) (scale 0–100)

Supplementary Figure 5. Editing Windows of Nme2-ABE8e Effectors at N$_4$CN PAM Sites. a) Summary of mean A-to-G editing activities and editing windows for eNme2-C, Nme2- and Nme2^{Smu}- ABE8e effectors at N$_4$CN PAM guide-target library members in HEK293T cells. **b)** Normalized **e**diting windows from (a); blue boxes refer to activity >50% of the window maximum.

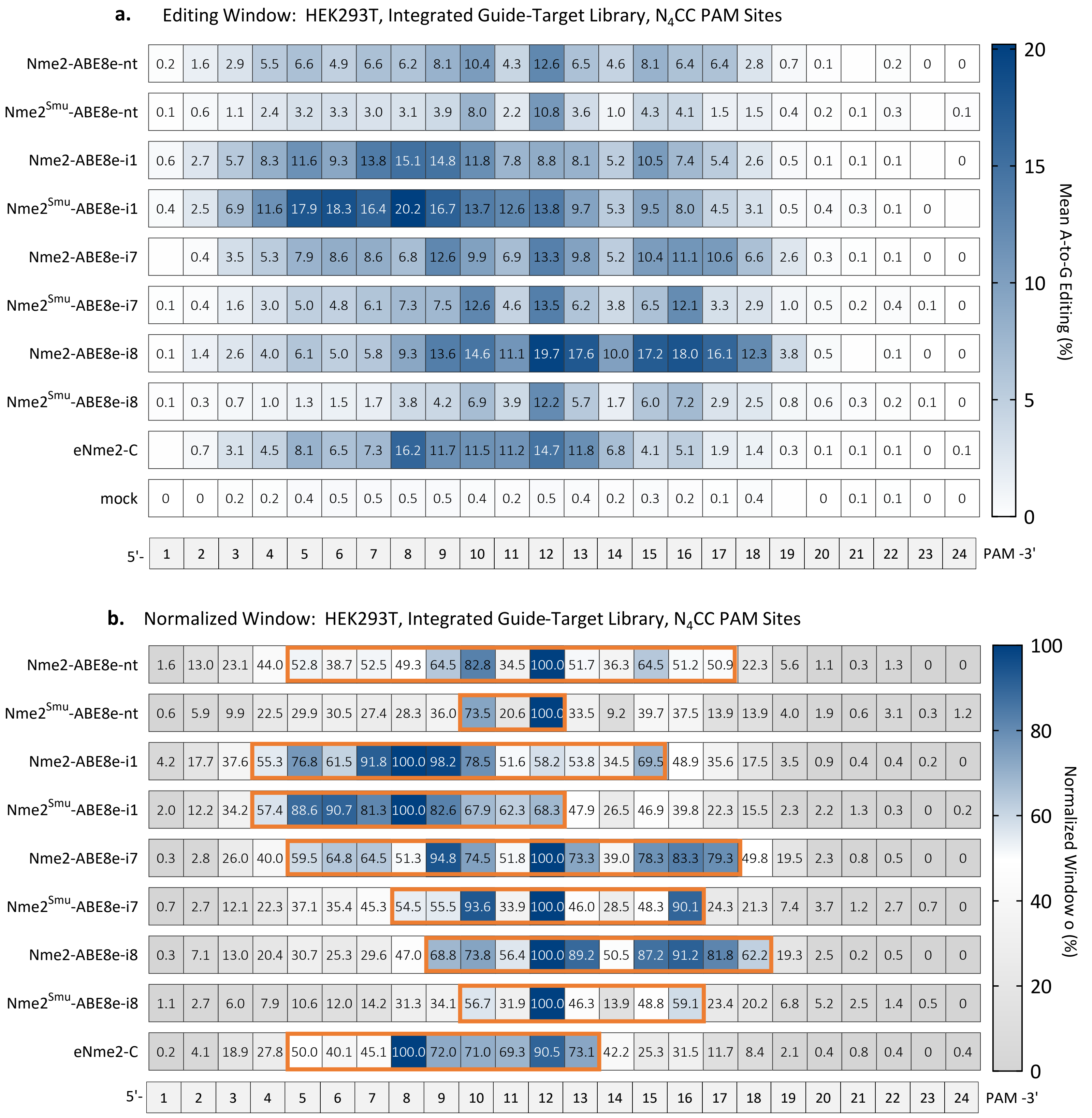

Supplementary Figure 6. Editing Windows of Nme2-ABE8e Effectors at N_4CC PAM Sites. a) Summary of mean A-to-G editing activities and editing windows for eNme2-C, Nme2- and Nme2^{Smu}- ABE8e effectors at N_4CC PAM guide-target library members in HEK293T cells. **b)** Normalized **e**diting windows from (**a**); orange boxes refer to activity >50% of the window maximum.

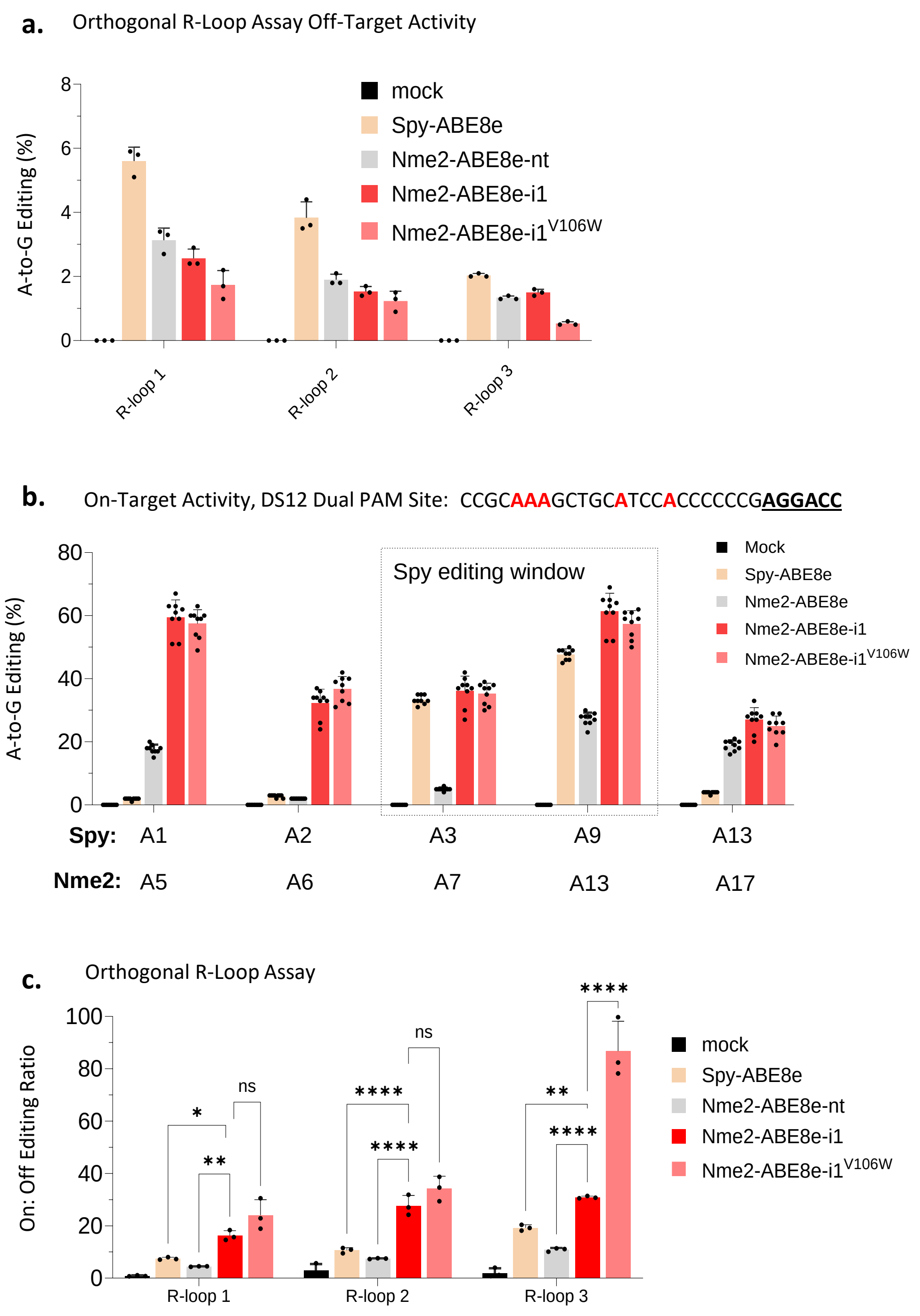

Supplementary Figure 7. Specificity of Domain-Inlaid Nme2Cas9-ABE8e. a) Guide-independent DNA off-target A-to-G editing at orthogonal SauCas9 R-loops measured via amplicon deep sequencing. SauCas9 HNH nickase was used to increase the sensitivity of editing at the orthogonal R-loops (n = 3 biological replicates; data represent mean ± SD). **b)** On-target activity of the ABE8e variants tested for the R-loop assay with a PAM-matched target site for Spy-ABE8e and Nme2-ABE8e effectors, measured via amplicon deep sequencing. Spy-ABE8e editing window is boxed. Overlapping target site sequence from 5' to 3' with adenines in red, and Spy- and Nme2- PAMs bold and underlined (n = 3 biological replicates per off-target R-loop in (**c**); data represent mean ± SD). **c)** Ratios of on-target vs. off-target editing of the ABE effectors tested at the overlapping *Linc01588* target site (see Supplementary Fig. 2A) and the orthogonal dSauCas9 R-loops (see Supplementary Fig. 2B) (n = 3 biological replicates, data represent mean ± SD). Two-way ANOVA analysis: ns, p > 0.05; *, p < 0.05; **, p < 0.01; ***, p < 0.001; ****, p < 0.0001. On-target editing efficiency for Spy-ABE8e is derived from the mean editing within its editing window, so as not to skew the ratio when compared to the wider on-target editing window of Nme2-ABE8e.

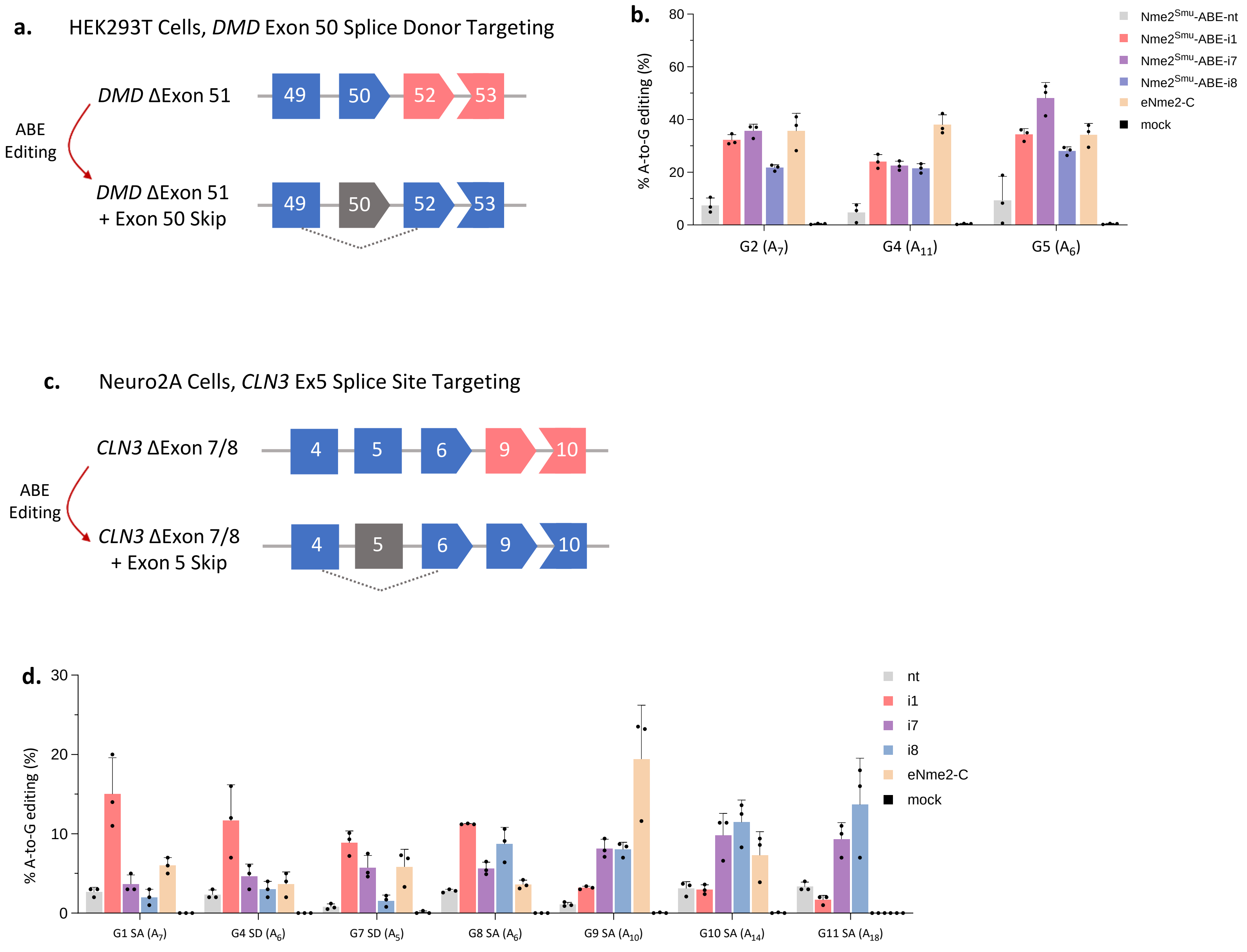

Supplementary Figure 8. Therapeutic Splice Site Disruption. a) Schematic of *DMD* exon 50 skipping approach to restore the *DMD* ΔExon 51 transcript reading frame. Top, *DMD* ΔEx51 generates a premature stop codon within *DMD* exon 52 (red). Bottom, ABE-mediated splice donor site (SDS) editing of *DMD* exon 50 by Nme2-ABE8e variants results in exon 50 skipping (gray), restoring the *DMD* reading frame. **b)** A-to-G editing of *DMD* exon 50 SDS in HEK293T cells, following transfection of eNme2-C and Nme2^{Smu}-ABE8e plasmids. Editing activities were measured by amplicon sequencing (n = 3 biological replicates; data represent mean ± SD). **c)** Schematic of *CLN3* exon 5 skipping to restore *CLN3* ΔExon 7/8 transcript reading frame. Top, *CLN3* ΔExon 7/8 generates a premature stop codon within *CLN3* exon 9 (red). Bottom, ABE-mediated splice site disruption of *CLN3* exon 5 by eNme2-C, Nme2- and Nme2^{Smu}-ABE8e variants for exon 5 skipping (gray), restoring the *CLN3* reading frame. **d)** A-to-G editing of mouse *Cln3* exon 5 splice donor or acceptor sites (SA, SD) in Neuro2A cells, following transfection of eNme2-C, Nme2- or Nme2^{Smu}-ABE8e plasmids. Editing activities were measured by amplicon sequencing (n = 3 biological replicates; data represent mean ± SD). Guide 1 and Guide 4 were edited with Nme2-ABE8e or eNme2-C, and Guides 7 to 11 were edited by Nme2^{Smu}-ABE8e or eNme2-C. Complete datasets can be found in Supplementary Table 1.

a.

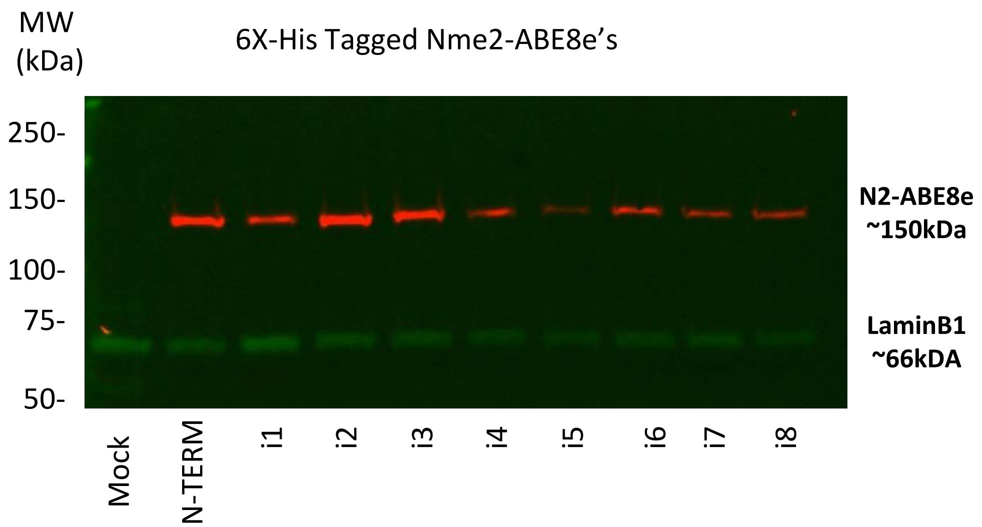

b.

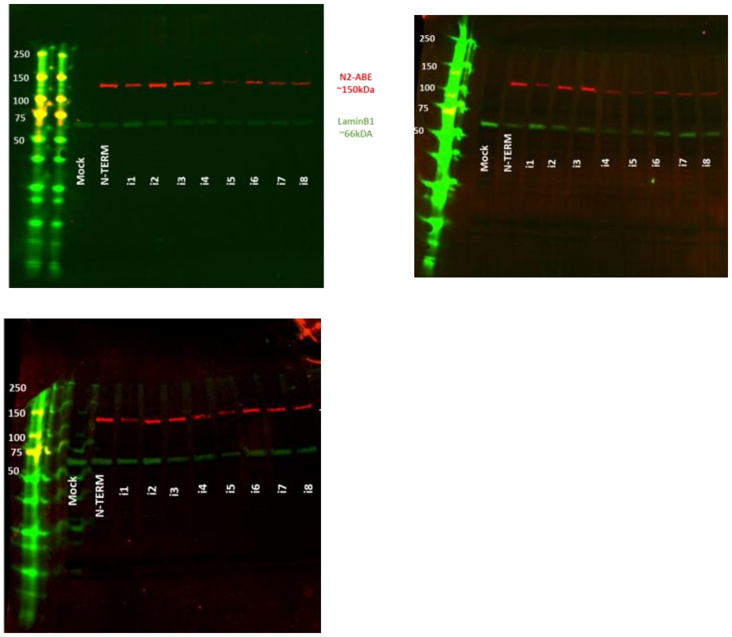

Supplementary Figure 9. Domain-Inlaid Nme2-ABE Expression. a) Nme2-ABE8e protein expression was confirmed by fluorescent western blot against the 6X-HIS tag epitope. Lamin-B1 (LMNB1) was used as a loading control. **b)** Uncropped images of triplicate data for Nme2-ABE8e variant expression with fluorescent western blot.

a.

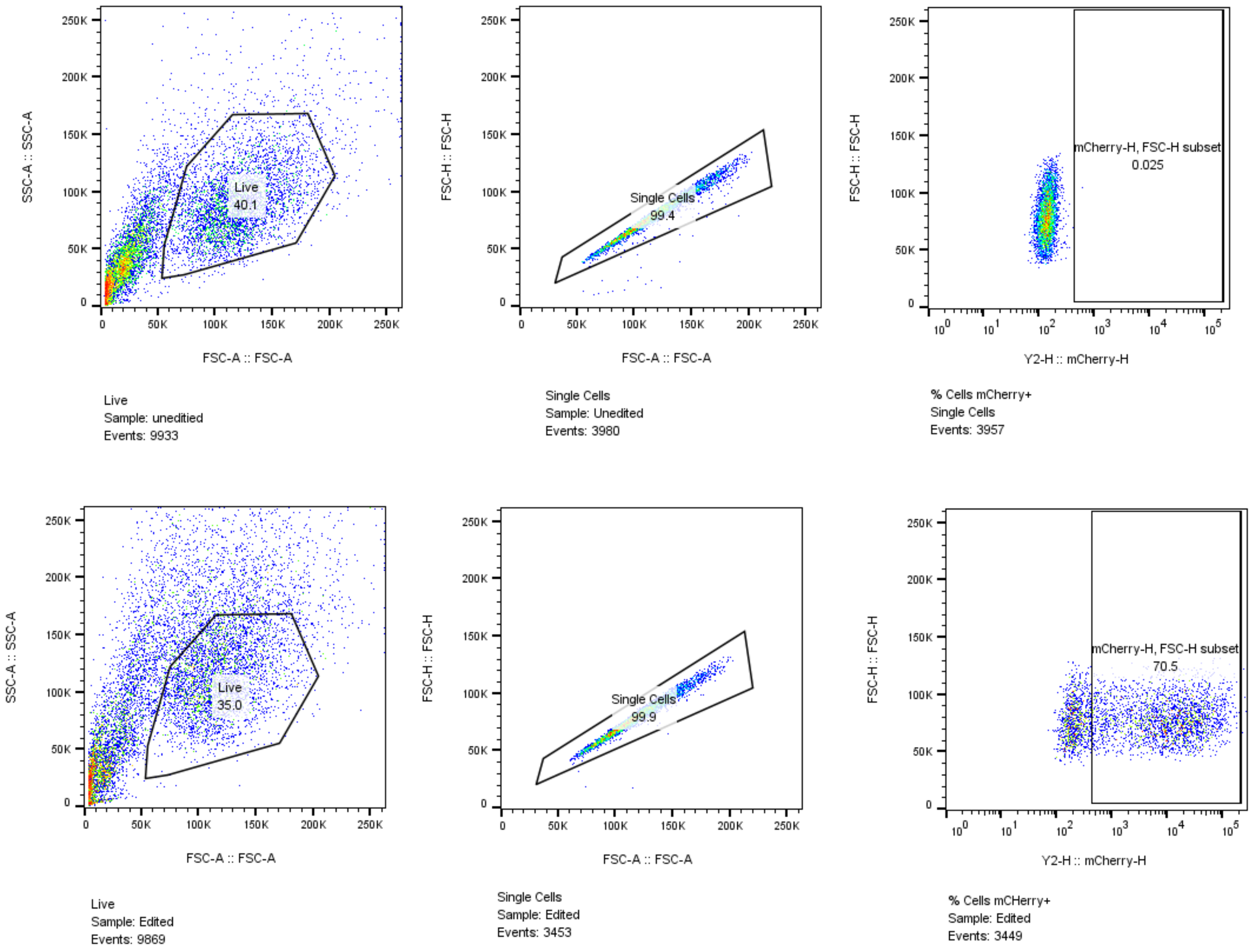

Supplementary Figure 10. Flow Cytometry Gating Strategy. a) Example gating strategy used for flow cytometry analysis for an unedited sample (top) or an edited sample (bottom).

Supplementary Note 1. Nucleotide sequences of Nme2Cas9 and Nme2^{Smu}Cas9 base editors described in this manuscript.

Nme2-ABE8e-nt: BPSV40-NLS, Nme2Cas9, TadA8e, Linkers

AAACGGACAGCCGACGGAAGCGAGTTCGAGTCACCAAAGAAGAAGCGGAAAGTCGGCGGTAGCGGCGGAGGCAGCGGTGGCGGCA
GCGGCTCTGAGGTGGAGTTTTCCCACGAGTACTGGATGAGACATGCCCTGACCCTGGCCAAGAGGGCACGCGATGAGAGGGAGGTGC
CTGTGGGAGCCGTGCTGGTGCTGAACAATAGAGTGATCGGCGAGGGCTGGAACAGAGCCATCGGCCTGCACGACCCAACAGCCCATG
CCGAAATTATGGCCCTGAGACAGGGCGGCCTGGTCATGCAGAACTACAGACTGATTGACGCCACCCTGTACGTGACATTCGAGCCTTG
CGTGATGTGCGCCGGCGCCATGATCCACTCTAGGATCGGCCGCGTGGTGTTTGGCGTGAGGAACAGCAAACGGGGCGCCGCAGGCTC
CCTGATGAACGTGCTGAACTACCCCGGCATGAATCACCGCGTCGAAATTACCGAGGGAATCCTGGCAGATGAATGTGCCGCCCTGCTG
TGCGACTTCTACCGGATGCCTAGACAGGTGTTCAATGCTCAGAAGAAGGCCCAGAGCTCCATCAACTCCGGAGGATCTAGCGGAGGCT
CCTCTGGCTCTGAGACACCTGGCACAAGCGAGAGCGCAACACCTGAAAGCAGCGGGGGCAGCAGCGGGGGGTCAATGGCCGCCTTCA
AGCCTAACCCAATCAATTACATCCTGGGACTGGCCATCGGAATCGCATCCGTGGGATGGGCTATGGTGGAGATCGACGAGGAGGAGA
ATCCTATCCGGCTGATCGATCTGGGCGTGAGAGTGTTTGAGAGGGCCGAGGTGCCAAAGACCGGCGATTCTCTGGCTATGGCCCGGA
GACTGGCACGGAGCGTGAGGCGCCTGACACGGAGAAGGGCACACAGGCTGCTGAGGGCACGCCGGCTGCTGAAGAGAGAGGGCGT
GCTGCAGGCAGCAGACTTCGATGAGAATGGCCTGATCAAGAGCCTGCCAAACACCCCCTGGCAGCTGAGAGCAGCCGCCCTGGACAG
GAAGCTGACACCACTGGAGTGGTCTGCCGTGCTGCTGCACCTGATCAAGCACCGCGGCTACCTGAGCCAGCGGAAGAACGAGGGAGA
GACAGCAGACAAGGAGCTGGGCGCCCTGCTGAAGGGAGTGGCCAACAATGCCCACGCCCTGCAGACCGGCGATTTCAGGACACCTGC
CGAGCTGGCCCTGAATAAGTTTGAGAAGGAGTCCGGCCACATCAGAAACCAGAGGGGCGACTATAGCCACACCTTCTCCCGCAAGGAT
CTGCAGGCCGAGCTGATCCTGCTGTTCGAGAAGCAGAAGGAGTTTGGCAATCCACACGTGAGCGGAGGCCTGAAGGAGGGAATCGA
GACCCTGCTGATGACACAGAGGCCTGCCCTGTCCGGCGACGCAGTGCAGAAGATGCTGGGACACTGCACCTTCGAGCCTGCAGAGCCA
AAGGCCGCCAAGAACACCTACACAGCCGAGCGGTTTATCTGGCTGACAAAGCTGAACAATCTGAGAATCCTGGAGCAGGGATCCGAG
AGGCCACTGACCGACACAGAGAGGGCCACCCTGATGGATGAGCCTTACCGGAAGTCTAAGCTGACATATGCCCAGGCCAGAAAGCTG
CTGGGCCTGGAGGACACCGCCTTCTTTAAGGGCCTGAGATACGGCAAGGATAATGCCGAGGCCTCCACACTGATGGAGATGAAGGCC
TATCACGCCATCTCTCGCGCCCTGGAGAAGGAGGGCCTGAAGGACAAGAAGTCCCCCCTGAACCTGAGCTCCGAGCTGCAGGATGAG
ATCGGCACCGCCTTCTCTCTGTTTAAGACCGACGAGGATATCACAGGCCGCCTGAAGGACAGGGTGCAGCCTGAGATCCTGGAGGCCC
TGCTGAAGCACATCTCTTTCGATAAGTTTGTGCAGATCAGCCTGAAGGCCCTGAGAAGGATCGTGCCACTGATGGAGCAGGGCAAGCG
GTACGACGAGGCCTGCGCCGAGATCTACGGCGATCACTATGGCAAGAAGAACACAGAGGAGAAGATCTATCTGCCCCCTATCCCTGCC
GACGAGATCAGAAATCCTGTGGTGCTGAGGGCCCTGTCCCAGGCAAGAAAAGTGATCAACGGAGTGGTGCGCCGGTACGGATCTCCA
GCCCGGATCCACATCGAGACCGCCAGAGAAGTGGGCAAGAGCTTCAAGGACCGGAAGGAGATCGAGAAGAGACAGGAGGAGAATC
GCAAGGATCGGGAGAAGGCCGCCGCCAAGTTTAGGGAGTACTTCCCTAACTTTGTGGGCGAGCCAAAGTCTAAGGACATCCTGAAGCT
GCGCCTGTACGAGCAGCAGCACGGCAAGTGTCTGTATAGCGGCAAGGAGATCAATCTGGTGCGGCTGAACGAGAAGGGCTATGTGG
AGATCGATCACGCCCTGCCTTTCTCCAGAACCTGGGACGATTCTTTTAACAATAAGGTGCTGGTGCTGGGCAGCGAGAACCAGAATAA
GGGCAATCAGACACCATACGAGTATTTCAATGGCAAGGACAACTCCAGGGAGTGGCAGGAGTTCAAGGCCCGCGTGGAGACCTCTAG
ATTTCCCAGGAGCAAGAAGCAGCGGATCCTGCTGCAGAAGTTCGACGAGGATGGCTTTAAGGAGTGCAACCTGAATGACACCAGATA
CGTGAACCGGTTCCTGTGCCAGTTTGTGGCCGATCACATCCTGCTGACCGGCAAGGGCAAGAGAAGGGTGTTCGCCTCTAATGGCCAG
ATCACAAACCTGCTGAGGGGATTTTGGGGACTGAGGAAGGTGCGGGCAGAGAATGACAGACACCACGCACTGGATGCAGTGGTGGT
GGCATGCAGCACCGTGGCAATGCAGCAGAAGATCACAAGATTCGTGAGGTATAAGGAGATGAACGCCTTTGACGGCAAGACCATCGA
TAAGGAGACAGGCAAGGTGCTGCACCAGAAGACCCACTTCCCCCAGCCTTGGGAGTTCTTTGCCCAGGAAGTGATGATCCGGGTGTTC
GGCAAGCCAGACGGCAAGCCTGAGTTTGAGGAGGCCGATACCCCAGAGAAGCTGAGGACACTGCTGGCAGAGAAGCTGTCTAGCAG
GCCAGAGGCAGTGCACGAGTACGTGACCCCACTGTTCGTGTCCAGGGCACCCAATCGGAAGATGTCTGGCGCCCACAAGGACACACTG
AGAAGCGCCAAGAGGTTTGTGAAGCACAACGAGAAGATCTCCGTGAAGAGAGTGTGGCTGACCGAGATCAAGCTGGCCGATCTGGA
GAACATGGTGAATTACAAGAACGGCAGGGAGATCGAGCTGTATGAGGCCCTGAAGGCAAGGCTGGAGGCCTACGGAGGAAATGCCA
AGCAGGCCTTCGACCCAAAGGATAACCCCTTTTATAAGAAGGGAGGACAGCTGGTGAAGGCCGTGCGGGTGGAGAAGACCCAGGAG
AGCGGCGTGCTGCTGAATAAGAAGAACGCCTACACAATCGCCGACAATGGCGATATGGTGAGAGTGGACGTGTTCTGTAAGGTGGAT
AAGAAGGGCAAGAATCAGTACTTTATCGTGCCTATCTATGCCTGGCAGGTGGCCGAGAACATCCTGCCAGACATCGATTGCAAGGGCT
ACAGAATCGACGATAGCTATACATTCTGTTTTTCCCTGCACAAGTATGACCTGATCGCCTTCCAGAAGGATGAGAAGTCCAAGGTGGAG
TTTGCCTACTATATCAATTGCGACTCCTCTAACGGCAGGTTCTACCTGGCCTGGCACGATAAGGGCAGCAAGGAGCAGCAGTTTCGCAT
CTCCACCCAGAATCTGGTGCTGATCCAGAAGTATCAGGTGAACGAGCTGGGCAAGGAGATCAGGCCATGTCGGCTGAAGAAGCGCCC
ACCCGTGCGGGAGGATAAAAGAACCGCCGACGGCAGCGAATTCGAGCCCAAGAAGAAGAGGAAAGTC

Nme2-ABE8e-i1: BPSV40-NLS, Nme2Cas9, TadA8e, Linkers

AAACGGACAGCCGACGGAAGCGAGTTCGAGTCACCAAAGAAGAAGCGGAAAGTCGAAGATATGGCCGCCTTCAAGCCTAACCCA
ATCAATTACATCCTGGGACTGGCCATCGGAATCGCATCCGTGGGATGGGCTATGGTGGAGATCGACGAGGAGGAGAATCCTATCC
GGCTGATCGATCTGGGCGTGAGAGTGTTTGAGAGGGCCGAGGTGCCAAAGACCGGCGATTCTCTGGCTATGGCCCGGAGACTGG
CACGGAGCGTGAGGCGCCTGACACGGAGAAGGGCACACAGGCTGCTGAGGGCACGCCGGCTGCTGAAGAGAGAGGGCGTGCTG
CAGGCAGCAGACTTCGATGAGAATGGCCTGATCAAGAGCCTGCCAAACACCCCCTGGCAGCTGAGAGCAGCCGCCCTGGACAGGA
AGCTGACACCACTGGAGTGGTCTGCCGTGCTGCTGCACCTGATCAAGCACCGCGGCTACCTGAGCCAGCGGAAGAACGAGGGAG
AGACAGCAGACAAGGAGCTGGGCGCCCTGCTGAAGGGAGTGGCCAACAATGCCCACGCCCTGCAGACCGGCGATTTCAGGACAC
CTGCCGAGCTGGCCCTGAATAAGTTTGAGAAGGAGTCCGGCCACATCAGAAACCAGAGGGGCGACTATAGCCACACCTTCTCCCG
CAAGGATCTGCAGGCCGAGCTGATCCTGCTGTTCGAGAAGCAGAAGGAGTTTGGCAATCCACACGTGAGCGGAGGCCTGAAGGA
GGGAATCGAGACCCTGCTGATGACACAGAGGCCTGCCCTGTCCGGCGACGCAGTGCAGAAGATGCTGGGACACTGCACCTTCGAG
CCTGCAGAGCCAAAGGCCGCCAAGAACACCTACACAGCCGAGCGGTTTATCTGGCTGACAAAGCTGAACAATCTGAGAATCCTGG
AGCAGGGCGGATCAGGAGGCTCTGGCGGTTCAGGTGGATCAGGCGGTAGCGGAGGTTCAGGTGGTTCTGAGGTGGAGTTTTCCC
ACGAGTACTGGATGAGACATGCCCTGACCCTGGCCAAGAGGGCACGCGATGAGAGGGAGGTGCCTGTGGGAGCCGTGCTGGTGC
TGAACAATAGAGTGATCGGCGAGGGCTGGAACAGAGCCATCGGCCTGCACGACCCAACAGCCCATGCCGAAATTATGGCCCTGAG
ACAGGGCGGCCTGGTCATGCAGAACTACAGACTGATTGACGCCACCCTGTACGTGACATTCGAGCCTTGCGTGATGTGCGCCGGC
GCCATGATCCACTCTAGGATCGGCCGCGTGGTGTTTGGCGTGAGGAACAGCAAACGGGGCGCCGCAGGCTCCCTGATGAACGTGC
TGAACTACCCCGGCATGAATCACCGCGTCGAAATTACCGAGGGAATCCTGGCAGATGAATGTGCCGCCCTGCTGTGCGACTTCTAC
CGGATGCCTAGACAGGTGTTCAATGCTCAGAAGAAGGCCCAGAGCTCCATCAACGGCTCCTCTGGCTCTGAGACACCTGGCACAA
GCGAGAGCGCAACACCTGAAAGCAGCGGCGGATCCGAGAGGCCACTGACCGACACAGAGAGGGCCACCCTGATGGATGAGCCTT
ACCGGAAGTCTAAGCTGACATATGCCCAGGCCAGAAAGCTGCTGGGCCTGGAGGACACCGCCTTCTTTAAGGGCCTGAGATACGG
CAAGGATAATGCCGAGGCCTCCACACTGATGGAGATGAAGGCCTATCACGCCATCTCTCGCGCCCTGGAGAAGGAGGGCCTGAAG
GACAAGAAGTCCCCCCTGAACCTGAGCTCCGAGCTGCAGGATGAGATCGGCACCGCCTTCTCTCTGTTTAAGACCGACGAGGATAT
CACAGGCCGCCTGAAGGACAGGGTGCAGCCTGAGATCCTGGAGGCCCTGCTGAAGCACATCTCTTTCGATAAGTTTGTGCAGATCA
GCCTGAAGGCCCTGAGAAGGATCGTGCCACTGATGGAGCAGGGCAAGCGGTACGACGAGGCCTGCGCCGAGATCTACGGCGATC
ACTATGGCAAGAAGAACACAGAGGAGAAGATCTATCTGCCCCCTATCCCTGCCGACGAGATCAGAAATCCTGTGGTGCTGAGGGC
CCTGTCCCAGGCAAGAAAAGTGATCAACGGAGTGGTGCGCCGGTACGGATCTCCAGCCCGGATCCACATCGAGACCGCCAGAGAA
GTGGGCAAGAGCTTCAAGGACCGGAAGGAGATCGAGAAGAGACAGGAGGAGAATCGCAAGGATCGGGAGAAGGCCGCCGCCA
AGTTTAGGGAGTACTTCCCTAACTTTGTGGGCGAGCCAAAGTCTAAGGACATCCTGAAGCTGCGCCTGTACGAGCAGCAGCACGG
CAAGTGTCTGTATAGCGGCAAGGAGATCAATCTGGTGCGGCTGAACGAGAAGGGCTATGTGGAGATCGATCACGCCCTGCCTTTC
TCCAGAACCTGGGACGATTCTTTTAACAATAAGGTGCTGGTGCTGGGCAGCGAGAACCAGAATAAGGGCAATCAGACACCATACG
AGTATTTCAATGGCAAGGACAACTCCAGGGAGTGGCAGGAGTTCAAGGCCCGCGTGGAGACCTCTAGATTTCCCAGGAGCAAGAA
GCAGCGGATCCTGCTGCAGAAGTTCGACGAGGATGGCTTTAAGGAGTGCAACCTGAATGACACCAGATACGTGAACCGGTTCCTG
TGCCAGTTTGTGGCCGATCACATCCTGCTGACCGGCAAGGGCAAGAGAAGGGTGTTCGCCTCTAATGGCCAGATCACAAACCTGCT
GAGGGGATTTTGGGGACTGAGGAAGGTGCGGGCAGAGAATGACAGACACCACGCACTGGATGCAGTGGTGGTGGCATGCAGCA
CCGTGGCAATGCAGCAGAAGATCACAAGATTCGTGAGGTATAAGGAGATGAACGCCTTTGACGGCAAGACCATCGATAAGGAGA
CAGGCAAGGTGCTGCACCAGAAGACCCACTTCCCCCAGCCTTGGGAGTTCTTTGCCCAGGAAGTGATGATCCGGGTGTTCGGCAA
GCCAGACGGCAAGCCTGAGTTTGAGGAGGCCGATACCCCAGAGAAGCTGAGGACACTGCTGGCAGAGAAGCTGTCTAGCAGGCC
AGAGGCAGTGCACGAGTACGTGACCCCACTGTTCGTGTCCAGGGCACCCAATCGGAAGATGTCTGGCGCCCACAAGGACACACTG
AGAAGCGCCAAGAGGTTTGTGAAGCACAACGAGAAGATCTCCGTGAAGAGAGTGTGGCTGACCGAGATCAAGCTGGCCGATCTG
GAGAACATGGTGAATTACAAGAACGGCAGGGAGATCGAGCTGTATGAGGCCCTGAAGGCAAGGCTGGAGGCCTACGGAGGAAA
TGCCAAGCAGGCCTTCGACCCAAAGGATAACCCCTTTTATAAGAAGGGAGGACAGCTGGTGAAGGCCGTGCGGGTGGAGAAGAC
CCAGGAGAGCGGCGTGCTGCTGAATAAGAAGAACGCCTACACAATCGCCGACAATGGCGATATGGTGAGAGTGGACGTGTTCTGT
AAGGTGGATAAGAAGGGCAAGAATCAGTACTTTATCGTGCCTATCTATGCCTGGCAGGTGGCCGAGAACATCCTGCCAGACATCG
ATTGCAAGGGCTACAGAATCGACGATAGCTATACATTCTGTTTTTCCCTGCACAAGTATGACCTGATCGCCTTCCAGAAGGATGAGA
AGTCCAAGGTGGAGTTTGCCTACTATATCAATTGCGACTCCTCTAACGGCAGGTTCTACCTGGCCTGGCACGATAAGGGCAGCAAG
GAGCAGCAGTTTCGCATCTCCACCCAGAATCTGGTGCTGATCCAGAAGTATCAGGTGAACGAGCTGGGCAAGGAGATCAGGCCAT
GTCGGCTGAAGAAGCGCCCACCCGTGCGGGAGGATAAAAGAACCGCCGACGGCAGCGAATTCGAGCCCAAGAAGAAGAGGAAA
GTC

Nme2-ABE8e-i2: BPSV40-NLS, Nme2Cas9, TadA8e, Linkers

AAACGGACAGCCGACGGAAGCGAGTTCGAGTCACCAAAGAAGAAGCGGAAAGTCGAAGATATGGCCGCCTTCAAGCCTAACCCA
ATCAATTACATCCTGGGACTGGCCATCGGAATCGCATCCGTGGGATGGGCTATGGTGGAGATCGACGAGGAGGAGAATCCTATCC
GGCTGATCGATCTGGGCGTGAGAGTGTTTGAGAGGGCCGAGGTGCCAAAGACCGGCGATTCTCTGGCTATGGCCCGGAGACTGG
CACGGAGCGTGAGGCGCCTGACACGGAGAAGGGCACACAGGCTGCTGAGGGCACGCCGGCTGCTGAAGAGAGAGGGCGTGCTG
CAGGCAGCAGACTTCGATGAGAATGGCCTGATCAAGAGCCTGCCAAACACCCCCTGGCAGCTGAGAGCAGCCGCCCTGGACAGGA
AGCTGACACCACTGGAGTGGTCTGCCGTGCTGCTGCACCTGATCAAGCACCGCGGCTACCTGAGCCAGCGGAAGAACGAGGGAG
AGACAGCAGACAAGGAGCTGGGCGCCCTGCTGAAGGGAGTGGCCAACAATGCCCACGCCCTGCAGACCGGCGATTTCAGGACAC
CTGCCGAGCTGGCCCTGAATAAGTTTGAGAAGGAGTCCGGCCACATCAGAAACCAGAGGGGCGACTATAGCCACACCTTCTCCCG
CAAGGATCTGCAGGCCGAGCTGATCCTGCTGTTCGAGAAGCAGAAGGAGTTTGGCAATCCACACGTGAGCGGAGGCCTGAAGGA
GGGAATCGAGACCCTGCTGATGACACAGAGGCCTGCCCTGTCCGGCGACGCAGTGCAGAAGATGCTGGGACACTGCACCTTCGAG
CCTGCAGAGCCAAAGGCCGCCAAGAACACCTACACAGCCGAGCGGTTTATCTGGCTGACAAAGCTGAACAATCTGAGAATCCTGG
AGCAGGGATCCGAGAGGCCACTGACCGACACAGAGAGGGCCACCCTGATGGATGAGCCTTACCGGAAGTCTAAGCTGACATATG
CCCAGGCCAGAAAGCTGCTGGGCCTGGAGGACGGCGGATCAGGAGGCTCTGGCGGTTCAGGTGGATCAGGCGGTAGCGGAGGT
TCAGGTGGTTCTGAGGTGGAGTTTTCCCACGAGTACTGGATGAGACATGCCCTGACCCTGGCCAAGAGGGCACGCGATGAGAGG
GAGGTGCCTGTGGGAGCCGTGCTGGTGCTGAACAATAGAGTGATCGGCGAGGGCTGGAACAGAGCCATCGGCCTGCACGACCCA
ACAGCCCATGCCGAAATTATGGCCCTGAGACAGGGCGGCCTGGTCATGCAGAACTACAGACTGATTGACGCCACCCTGTACGTGA
CATTCGAGCCTTGCGTGATGTGCGCCGGCGCCATGATCCACTCTAGGATCGGCCGCGTGGTGTTTGGCGTGAGGAACAGCAAACG
GGGCGCCGCAGGCTCCCTGATGAACGTGCTGAACTACCCCGGCATGAATCACCGCGTCGAAATTACCGAGGGAATCCTGGCAGAT
GAATGTGCCGCCCTGCTGTGCGACTTCTACCGGATGCCTAGACAGGTGTTCAATGCTCAGAAGAAGGCCCAGAGCTCCATCAACGG
CTCCTCTGGCTCTGAGACACCTGGCACAAGCGAGAGCGCAACACCTGAAAGCAGCGGCACCGCCTTCTTTAAGGGCCTGAGATAC
GGCAAGGATAATGCCGAGGCCTCCACACTGATGGAGATGAAGGCCTATCACGCCATCTCTCGCGCCCTGGAGAAGGAGGGCCTGA
AGGACAAGAAGTCCCCCCTGAACCTGAGCTCCGAGCTGCAGGATGAGATCGGCACCGCCTTCTCTCTGTTTAAGACCGACGAGGAT
ATCACAGGCCGCCTGAAGGACAGGGTGCAGCCTGAGATCCTGGAGGCCCTGCTGAAGCACATCTCTTTCGATAAGTTTGTGCAGAT
CAGCCTGAAGGCCCTGAGAAGGATCGTGCCACTGATGGAGCAGGGCAAGCGGTACGACGAGGCCTGCGCCGAGATCTACGGCGA
TCACTATGGCAAGAAGAACACAGAGGAGAAGATCTATCTGCCCCCTATCCCTGCCGACGAGATCAGAAATCCTGTGGTGCTGAGG
GCCCTGTCCCAGGCAAGAAAAGTGATCAACGGAGTGGTGCGCCGGTACGGATCTCCAGCCCGGATCCACATCGAGACCGCCAGAG
AAGTGGGCAAGAGCTTCAAGGACCGGAAGGAGATCGAGAAGAGACAGGAGGAGAATCGCAAGGATCGGGAGAAGGCCGCCGC
CAAGTTTAGGGAGTACTTCCCTAACTTTGTGGGCGAGCCAAAGTCTAAGGACATCCTGAAGCTGCGCCTGTACGAGCAGCAGCACG
GCAAGTGTCTGTATAGCGGCAAGGAGATCAATCTGGTGCGGCTGAACGAGAAGGGCTATGTGGAGATCGATCACGCCCTGCCTTT
CTCCAGAACCTGGGACGATTCTTTTAACAATAAGGTGCTGGTGCTGGGCAGCGAGAACCAGAATAAGGGCAATCAGACACCATAC
GAGTATTTCAATGGCAAGGACAACTCCAGGGAGTGGCAGGAGTTCAAGGCCCGCGTGGAGACCTCTAGATTTCCCAGGAGCAAGA
AGCAGCGGATCCTGCTGCAGAAGTTCGACGAGGATGGCTTTAAGGAGTGCAACCTGAATGACACCAGATACGTGAACCGGTTCCT
GTGCCAGTTTGTGGCCGATCACATCCTGCTGACCGGCAAGGGCAAGAGAAGGGTGTTCGCCTCTAATGGCCAGATCACAAACCTG
CTGAGGGGATTTTGGGGACTGAGGAAGGTGCGGGCAGAGAATGACAGACACCACGCACTGGATGCAGTGGTGGTGGCATGCAG
CACCGTGGCAATGCAGCAGAAGATCACAAGATTCGTGAGGTATAAGGAGATGAACGCCTTTGACGGCAAGACCATCGATAAGGA
GACAGGCAAGGTGCTGCACCAGAAGACCCACTTCCCCCAGCCTTGGGAGTTCTTTGCCCAGGAAGTGATGATCCGGGTGTTCGGC
AAGCCAGACGGCAAGCCTGAGTTTGAGGAGGCCGATACCCCAGAGAAGCTGAGGACACTGCTGGCAGAGAAGCTGTCTAGCAGG
CCAGAGGCAGTGCACGAGTACGTGACCCCACTGTTCGTGTCCAGGGCACCCAATCGGAAGATGTCTGGCGCCCACAAGGACACAC
TGAGAAGCGCCAAGAGGTTTGTGAAGCACAACGAGAAGATCTCCGTGAAGAGAGTGTGGCTGACCGAGATCAAGCTGGCCGATC
TGGAGAACATGGTGAATTACAAGAACGGCAGGGAGATCGAGCTGTATGAGGCCCTGAAGGCAAGGCTGGAGGCCTACGGAGGA
AATGCCAAGCAGGCCTTCGACCCAAAGGATAACCCCTTTTATAAGAAGGGAGGACAGCTGGTGAAGGCCGTGCGGGTGGAGAAG
ACCCAGGAGAGCGGCGTGCTGCTGAATAAGAAGAACGCCTACACAATCGCCGACAATGGCGATATGGTGAGAGTGGACGTGTTCT
GTAAGGTGGATAAGAAGGGCAAGAATCAGTACTTTATCGTGCCTATCTATGCCTGGCAGGTGGCCGAGAACATCCTGCCAGACAT
CGATTGCAAGGGCTACAGAATCGACGATAGCTATACATTCTGTTTTTCCCTGCACAAGTATGACCTGATCGCCTTCCAGAAGGATGA
GAAGTCCAAGGTGGAGTTTGCCTACTATATCAATTGCGACTCCTCTAACGGCAGGTTCTACCTGGCCTGGCACGATAAGGGCAGCA
AGGAGCAGCAGTTTCGCATCTCCACCCAGAATCTGGTGCTGATCCAGAAGTATCAGGTGAACGAGCTGGGCAAGGAGATCAGGCC
ATGTCGGCTGAAGAAGCGCCCACCCGTGCGGGAGGATAAAAGAACCGCCGACGGCAGCGAATTCGAGCCCAAGAAGAAGAGGA
AAGTC

Nme2-ABE8e-i3: BPSV40-NLS, Nme2Cas9, TadA8e, Linkers

AAACGGACAGCCGACGGAAGCGAGTTCGAGTCACCAAAGAAGAAGCGGAAAGTCGAAGATATGGCCGCCTTCAAGCCTAACCCA
ATCAATTACATCCTGGGACTGGCCATCGGAATCGCATCCGTGGGATGGGCTATGGTGGAGATCGACGAGGAGGAGAATCCTATCC
GGCTGATCGATCTGGGCGTGAGAGTGTTTGAGAGGGCCGAGGTGCCAAAGACCGGCGATTCTCTGGCTATGGCCCGGAGACTGG
CACGGAGCGTGAGGCGCCTGACACGGAGAAGGGCACACAGGCTGCTGAGGGCACGCCGGCTGCTGAAGAGAGAGGGCGTGCTG
CAGGCAGCAGACTTCGATGAGAATGGCCTGATCAAGAGCCTGCCAAACACCCCCTGGCAGCTGAGAGCAGCCGCCCTGGACAGGA
AGCTGACACCACTGGAGTGGTCTGCCGTGCTGCTGCACCTGATCAAGCACCGCGGCTACCTGAGCCAGCGGAAGAACGAGGGAG
AGACAGCAGACAAGGAGCTGGGCGCCCTGCTGAAGGGAGTGGCCAACAATGCCCACGCCCTGCAGACCGGCGATTTCAGGACAC
CTGCCGAGCTGGCCCTGAATAAGTTTGAGAAGGAGTCCGGCCACATCAGAAACCAGAGGGGCGACTATAGCCACACCTTCTCCCG
CAAGGATCTGCAGGCCGAGCTGATCCTGCTGTTCGAGAAGCAGAAGGAGTTTGGCAATCCACACGTGAGCGGAGGCCTGAAGGA
GGGAATCGAGACCCTGCTGATGACACAGAGGCCTGCCCTGTCCGGCGACGCAGTGCAGAAGATGCTGGGACACTGCACCTTCGAG
CCTGCAGAGCCAAAGGCCGCCAAGAACACCTACACAGCCGAGCGGTTTATCTGGCTGACAAAGCTGAACAATCTGAGAATCCTGG
AGCAGGGATCCGAGAGGCCACTGACCGACACAGAGAGGGCCACCCTGATGGATGAGCCTTACCGGAAGTCTAAGCTGACATATG
CCCAGGCCAGAAAGCTGCTGGGCCTGGAGGACACCGCCTTCTTTAAGGGCCTGAGATACGGCAAGGGCGGATCAGGAGGCTCTG
GCGGTTCAGGTGGATCAGGCGGTAGCGGAGGTTCAGGTGGTTCTGAGGTGGAGTTTTCCCACGAGTACTGGATGAGACATGCCCT
GACCCTGGCCAAGAGGGCACGCGATGAGAGGGAGGTGCCTGTGGGAGCCGTGCTGGTGCTGAACAATAGAGTGATCGGCGAGG
GCTGGAACAGAGCCATCGGCCTGCACGACCCAACAGCCCATGCCGAAATTATGGCCCTGAGACAGGGCGGCCTGGTCATGCAGAA
CTACAGACTGATTGACGCCACCCTGTACGTGACATTCGAGCCTTGCGTGATGTGCGCCGGCGCCATGATCCACTCTAGGATCGGCC
GCGTGGTGTTTGGCGTGAGGAACAGCAAACGGGGCGCCGCAGGCTCCCTGATGAACGTGCTGAACTACCCCGGCATGAATCACCG
CGTCGAAATTACCGAGGGAATCCTGGCAGATGAATGTGCCGCCCTGCTGTGCGACTTCTACCGGATGCCTAGACAGGTGTTCAATG
CTCAGAAGAAGGCCCAGAGCTCCATCAACGGCTCCTCTGGCTCTGAGACACCTGGCACAAGCGAGAGCGCAACACCTGAAAGCAG
CGGCGATAATGCCGAGGCCTCCACACTGATGGAGATGAAGGCCTATCACGCCATCTCTCGCGCCCTGGAGAAGGAGGGCCTGAAG
GACAAGAAGTCCCCCCTGAACCTGAGCTCCGAGCTGCAGGATGAGATCGGCACCGCCTTCTCTCTGTTTAAGACCGACGAGGATAT
CACAGGCCGCCTGAAGGACAGGGTGCAGCCTGAGATCCTGGAGGCCCTGCTGAAGCACATCTCTTTCGATAAGTTTGTGCAGATCA
GCCTGAAGGCCCTGAGAAGGATCGTGCCACTGATGGAGCAGGGCAAGCGGTACGACGAGGCCTGCGCCGAGATCTACGGCGATC
ACTATGGCAAGAAGAACACAGAGGAGAAGATCTATCTGCCCCCTATCCCTGCCGACGAGATCAGAAATCCTGTGGTGCTGAGGGC
CCTGTCCCAGGCAAGAAAAGTGATCAACGGAGTGGTGCGCCGGTACGGATCTCCAGCCCGGATCCACATCGAGACCGCCAGAGAA
GTGGGCAAGAGCTTCAAGGACCGGAAGGAGATCGAGAAGAGACAGGAGGAGAATCGCAAGGATCGGGAGAAGGCCGCCGCCA
AGTTTAGGGAGTACTTCCCTAACTTTGTGGGCGAGCCAAAGTCTAAGGACATCCTGAAGCTGCGCCTGTACGAGCAGCAGCACGG
CAAGTGTCTGTATAGCGGCAAGGAGATCAATCTGGTGCGGCTGAACGAGAAGGGCTATGTGGAGATCGATCACGCCCTGCCTTTC
TCCAGAACCTGGGACGATTCTTTTAACAATAAGGTGCTGGTGCTGGGCAGCGAGAACCAGAATAAGGGCAATCAGACACCATACG
AGTATTTCAATGGCAAGGACAACTCCAGGGAGTGGCAGGAGTTCAAGGCCCGCGTGGAGACCTCTAGATTTCCCAGGAGCAAGAA
GCAGCGGATCCTGCTGCAGAAGTTCGACGAGGATGGCTTTAAGGAGTGCAACCTGAATGACACCAGATACGTGAACCGGTTCCTG
TGCCAGTTTGTGGCCGATCACATCCTGCTGACCGGCAAGGGCAAGAGAAGGGTGTTCGCCTCTAATGGCCAGATCACAAACCTGCT
GAGGGGATTTTGGGGACTGAGGAAGGTGCGGGCAGAGAATGACAGACACCACGCACTGGATGCAGTGGTGGTGGCATGCAGCA
CCGTGGCAATGCAGCAGAAGATCACAAGATTCGTGAGGTATAAGGAGATGAACGCCTTTGACGGCAAGACCATCGATAAGGAGA
CAGGCAAGGTGCTGCACCAGAAGACCCACTTCCCCCAGCCTTGGGAGTTCTTTGCCCAGGAAGTGATGATCCGGGTGTTCGGCAA
GCCAGACGGCAAGCCTGAGTTTGAGGAGGCCGATACCCCAGAGAAGCTGAGGACACTGCTGGCAGAGAAGCTGTCTAGCAGGCC
AGAGGCAGTGCACGAGTACGTGACCCCACTGTTCGTGTCCAGGGCACCCAATCGGAAGATGTCTGGCGCCCACAAGGACACACTG
AGAAGCGCCAAGAGGTTTGTGAAGCACAACGAGAAGATCTCCGTGAAGAGAGTGTGGCTGACCGAGATCAAGCTGGCCGATCTG
GAGAACATGGTGAATTACAAGAACGGCAGGGAGATCGAGCTGTATGAGGCCCTGAAGGCAAGGCTGGAGGCCTACGGAGGAAA
TGCCAAGCAGGCCTTCGACCCAAAGGATAACCCCTTTTATAAGAAGGGAGGACAGCTGGTGAAGGCCGTGCGGGTGGAGAAGAC
CCAGGAGAGCGGCGTGCTGCTGAATAAGAAGAACGCCTACACAATCGCCGACAATGGCGATATGGTGAGAGTGGACGTGTTCTGT
AAGGTGGATAAGAAGGGCAAGAATCAGTACTTTATCGTGCCTATCTATGCCTGGCAGGTGGCCGAGAACATCCTGCCAGACATCG
ATTGCAAGGGCTACAGAATCGACGATAGCTATACATTCTGTTTTTCCCTGCACAAGTATGACCTGATCGCCTTCCAGAAGGATGAGA
AGTCCAAGGTGGAGTTTGCCTACTATATCAATTGCGACTCCTCTAACGGCAGGTTCTACCTGGCCTGGCACGATAAGGGCAGCAAG
GAGCAGCAGTTTCGCATCTCCACCCAGAATCTGGTGCTGATCCAGAAGTATCAGGTGAACGAGCTGGGCAAGGAGATCAGGCCAT
GTCGGCTGAAGAAGCGCCCACCCGTGCGGGAGGATAAAAGAACCGCCGACGGCAGCGAATTCGAGCCCAAGAAGAAGAGGAAA
GTC

Nme2-ABE8e-i4: BPSV40-NLS, Nme2Cas9, TadA8e, Linkers

AAACGGACAGCCGACGGAAGCGAGTTCGAGTCACCAAAGAAGAAGCGGAAAGTCGAAGATATGGCCGCCTTCAAGCCTAACCCA
ATCAATTACATCCTGGGACTGGCCATCGGAATCGCATCCGTGGGATGGGCTATGGTGGAGATCGACGAGGAGGAGAATCCTATCC
GGCTGATCGATCTGGGCGTGAGAGTGTTTGAGAGGGCCGAGGTGCCAAAGACCGGCGATTCTCTGGCTATGGCCCGGAGACTGG
CACGGAGCGTGAGGCGCCTGACACGGAGAAGGGCACACAGGCTGCTGAGGGCACGCCGGCTGCTGAAGAGAGAGGGCGTGCTG
CAGGCAGCAGACTTCGATGAGAATGGCCTGATCAAGAGCCTGCCAAACACCCCCTGGCAGCTGAGAGCAGCCGCCCTGGACAGGA
AGCTGACACCACTGGAGTGGTCTGCCGTGCTGCTGCACCTGATCAAGCACCGCGGCTACCTGAGCCAGCGGAAGAACGAGGGAG
AGACAGCAGACAAGGAGCTGGGCGCCCTGCTGAAGGGAGTGGCCAACAATGCCCACGCCCTGCAGACCGGCGATTTCAGGACAC
CTGCCGAGCTGGCCCTGAATAAGTTTGAGAAGGAGTCCGGCCACATCAGAAACCAGAGGGGCGACTATAGCCACACCTTCTCCCG
CAAGGATCTGCAGGCCGAGCTGATCCTGCTGTTCGAGAAGCAGAAGGAGTTTGGCAATCCACACGTGAGCGGAGGCCTGAAGGA
GGGAATCGAGACCCTGCTGATGACACAGAGGCCTGCCCTGTCCGGCGACGCAGTGCAGAAGATGCTGGGACACTGCACCTTCGAG
CCTGCAGAGCCAAAGGCCGCCAAGAACACCTACACAGCCGAGCGGTTTATCTGGCTGACAAAGCTGAACAATCTGAGAATCCTGG
AGCAGGGATCCGAGAGGCCACTGACCGACACAGAGAGGGCCACCCTGATGGATGAGCCTTACCGGAAGTCTAAGCTGACATATG
CCCAGGCCAGAAAGCTGCTGGGCCTGGAGGACACCGCCTTCTTTAAGGGCCTGAGATACGGCAAGGATAATGCCGAGGCCTCCAC
ACTGATGGAGATGAAGGCCTATCACGCCATCTCTCGCGCCCTGGAGAAGGAGGGCCTGAAGGACAAGAAGTCCCCCCTGAACCTG
AGCTCCGAGCTGCAGGATGAGATCGGCACCGCCTTCTCTCTGTTTAAGACCGACGAGGATATCACAGGCCGCCTGAAGGACAGGG
TGCAGCCTGAGATCCTGGAGGCCCTGCTGAAGCACATCTCTTTCGATAAGTTTGTGCAGATCAGCCTGAAGGCCCTGAGAAGGATC
GTGCCACTGATGGAGCAGGGCAAGCGGTACGACGAGGCCTGCGCCGAGATCTACGGCGATCACTATGGCAAGAAGAACACAGAG
GAGAAGATCTATCTGCCCCCTATCCCTGCCGACGAGATCAGAAATCCTGTGGTGCTGAGGGCCCTGTCCCAGGCAAGAAAAGTGAT
CAACGGAGTGGTGCGCCGGTACGGATCTCCAGCCCGGATCCACATCGAGACCGCCAGAGAAGTGGGCAAGAGCTTCAAGGACCG
GAAGGAGATCGAGAAGAGACAGGAGGAGAATCGCAAGGATCGGGAGAAGGCCGCCGCCAAGTTTAGGGAGTACTTCCCTAACTT
TGTGGGCGAGCCAAAGTCTAAGGACATCCTGAAGCTGCGCCTGTACGAGCAGCAGCACGGCAAGTGTCTGTATAGCGGCAAGGA
GATCAATCTGGTGCGGCTGAACGAGAAGGGCTATGTGGAGATCGATCACGCCCTGCCTTTCTCCAGAACCTGGGACGATTCTTTTA
ACAATAAGGTGCTGGTGCTGGGCAGCGAGAACCAGAATAAGGGCAATCAGACACCATACGAGTATTTCAATGGCAAGGACAACTC
CAGGGAGTGGCAGGAGTTCAAGGCCCGCGTGGAGACCTCTAGAGGCGGATCAGGAGGCTCTGGCGGTTCAGGTGGATCAGGCG
GTAGCGGAGGTTCAGGTGGTTCTGAGGTGGAGTTTTCCCACGAGTACTGGATGAGACATGCCCTGACCCTGGCCAAGAGGGCACG
CGATGAGAGGGAGGTGCCTGTGGGAGCCGTGCTGGTGCTGAACAATAGAGTGATCGGCGAGGGCTGGAACAGAGCCATCGGCC
TGCACGACCCAACAGCCCATGCCGAAATTATGGCCCTGAGACAGGGCGGCCTGGTCATGCAGAACTACAGACTGATTGACGCCAC
CCTGTACGTGACATTCGAGCCTTGCGTGATGTGCGCCGGCGCCATGATCCACTCTAGGATCGGCCGCGTGGTGTTTGGCGTGAGGA
ACAGCAAACGGGGCGCCGCAGGCTCCCTGATGAACGTGCTGAACTACCCCGGCATGAATCACCGCGTCGAAATTACCGAGGGAAT
CCTGGCAGATGAATGTGCCGCCCTGCTGTGCGACTTCTACCGGATGCCTAGACAGGTGTTCAATGCTCAGAAGAAGGCCCAGAGCT
CCATCAACGGCTCCTCTGGCTCTGAGACACCTGGCACAAGCGAGAGCGCAACACCTGAAAGCAGCGGCTTTCCCAGGAGCAAGAA
GCAGCGGATCCTGCTGCAGAAGTTCGACGAGGATGGCTTTAAGGAGTGCAACCTGAATGACACCAGATACGTGAACCGGTTCCTG
TGCCAGTTTGTGGCCGATCACATCCTGCTGACCGGCAAGGGCAAGAGAAGGGTGTTCGCCTCTAATGGCCAGATCACAAACCTGCT
GAGGGGATTTTGGGGACTGAGGAAGGTGCGGGCAGAGAATGACAGACACCACGCACTGGATGCAGTGGTGGTGGCATGCAGCA
CCGTGGCAATGCAGCAGAAGATCACAAGATTCGTGAGGTATAAGGAGATGAACGCCTTTGACGGCAAGACCATCGATAAGGAGA
CAGGCAAGGTGCTGCACCAGAAGACCCACTTCCCCCAGCCTTGGGAGTTCTTTGCCCAGGAAGTGATGATCCGGGTGTTCGGCAA
GCCAGACGGCAAGCCTGAGTTTGAGGAGGCCGATACCCCAGAGAAGCTGAGGACACTGCTGGCAGAGAAGCTGTCTAGCAGGCC
AGAGGCAGTGCACGAGTACGTGACCCCACTGTTCGTGTCCAGGGCACCCAATCGGAAGATGTCTGGCGCCCACAAGGACACACTG
AGAAGCGCCAAGAGGTTTGTGAAGCACAACGAGAAGATCTCCGTGAAGAGAGTGTGGCTGACCGAGATCAAGCTGGCCGATCTG
GAGAACATGGTGAATTACAAGAACGGCAGGGAGATCGAGCTGTATGAGGCCCTGAAGGCAAGGCTGGAGGCCTACGGAGGAAA
TGCCAAGCAGGCCTTCGACCCAAAGGATAACCCCTTTTATAAGAAGGGAGGACAGCTGGTGAAGGCCGTGCGGGTGGAGAAGAC
CCAGGAGAGCGGCGTGCTGCTGAATAAGAAGAACGCCTACACAATCGCCGACAATGGCGATATGGTGAGAGTGGACGTGTTCTGT
AAGGTGGATAAGAAGGGCAAGAATCAGTACTTTATCGTGCCTATCTATGCCTGGCAGGTGGCCGAGAACATCCTGCCAGACATCG
ATTGCAAGGGCTACAGAATCGACGATAGCTATACATTCTGTTTTTCCCTGCACAAGTATGACCTGATCGCCTTCCAGAAGGATGAGA
AGTCCAAGGTGGAGTTTGCCTACTATATCAATTGCGACTCCTCTAACGGCAGGTTCTACCTGGCCTGGCACGATAAGGGCAGCAAG
GAGCAGCAGTTTCGCATCTCCACCCAGAATCTGGTGCTGATCCAGAAGTATCAGGTGAACGAGCTGGGCAAGGAGATCAGGCCAT
GTCGGCTGAAGAAGCGCCCACCCGTGCGGGAGGATAAAAGAACCGCCGACGGCAGCGAATTCGAGCCCAAGAAGAAGAGGAAA
GTC

Nme2-ABE8e-i5: BPSV40-NLS, Nme2Cas9, TadA8e, Linkers

AAACGGACAGCCGACGGAAGCGAGTTCGAGTCACCAAAGAAGAAGCGGAAAGTCGAAGATATGGCCGCCTTCAAGCCTAACCCA
ATCAATTACATCCTGGGACTGGCCATCGGAATCGCATCCGTGGGATGGGCTATGGTGGAGATCGACGAGGAGGAGAATCCTATCC
GGCTGATCGATCTGGGCGTGAGAGTGTTTGAGAGGGCCGAGGTGCCAAAGACCGGCGATTCTCTGGCTATGGCCCGGAGACTGG
CACGGAGCGTGAGGCGCCTGACACGGAGAAGGGCACACAGGCTGCTGAGGGCACGCCGGCTGCTGAAGAGAGAGGGCGTGCTG
CAGGCAGCAGACTTCGATGAGAATGGCCTGATCAAGAGCCTGCCAAACACCCCCTGGCAGCTGAGAGCAGCCGCCCTGGACAGGA
AGCTGACACCACTGGAGTGGTCTGCCGTGCTGCTGCACCTGATCAAGCACCGCGGCTACCTGAGCCAGCGGAAGAACGAGGGAG
AGACAGCAGACAAGGAGCTGGGCGCCCTGCTGAAGGGAGTGGCCAACAATGCCCACGCCCTGCAGACCGGCGATTTCAGGACAC
CTGCCGAGCTGGCCCTGAATAAGTTTGAGAAGGAGTCCGGCCACATCAGAAACCAGAGGGGCGACTATAGCCACACCTTCTCCCG
CAAGGATCTGCAGGCCGAGCTGATCCTGCTGTTCGAGAAGCAGAAGGAGTTTGGCAATCCACACGTGAGCGGAGGCCTGAAGGA
GGGAATCGAGACCCTGCTGATGACACAGAGGCCTGCCCTGTCCGGCGACGCAGTGCAGAAGATGCTGGGACACTGCACCTTCGAG
CCTGCAGAGCCAAAGGCCGCCAAGAACACCTACACAGCCGAGCGGTTTATCTGGCTGACAAAGCTGAACAATCTGAGAATCCTGG
AGCAGGGATCCGAGAGGCCACTGACCGACACAGAGAGGGCCACCCTGATGGATGAGCCTTACCGGAAGTCTAAGCTGACATATG
CCCAGGCCAGAAAGCTGCTGGGCCTGGAGGACACCGCCTTCTTTAAGGGCCTGAGATACGGCAAGGATAATGCCGAGGCCTCCAC
ACTGATGGAGATGAAGGCCTATCACGCCATCTCTCGCGCCCTGGAGAAGGAGGGCCTGAAGGACAAGAAGTCCCCCCTGAACCTG
AGCTCCGAGCTGCAGGATGAGATCGGCACCGCCTTCTCTCTGTTTAAGACCGACGAGGATATCACAGGCCGCCTGAAGGACAGGG
TGCAGCCTGAGATCCTGGAGGCCCTGCTGAAGCACATCTCTTTCGATAAGTTTGTGCAGATCAGCCTGAAGGCCCTGAGAAGGATC
GTGCCACTGATGGAGCAGGGCAAGCGGTACGACGAGGCCTGCGCCGAGATCTACGGCGATCACTATGGCAAGAAGAACACAGAG
GAGAAGATCTATCTGCCCCCTATCCCTGCCGACGAGATCAGAAATCCTGTGGTGCTGAGGGCCCTGTCCCAGGCAAGAAAAGTGAT
CAACGGAGTGGTGCGCCGGTACGGATCTCCAGCCCGGATCCACATCGAGACCGCCAGAGAAGTGGGCAAGAGCTTCAAGGACCG
GAAGGAGATCGAGAAGAGACAGGAGGAGAATCGCAAGGATCGGGAGAAGGCCGCCGCCAAGTTTAGGGAGTACTTCCCTAACTT
TGTGGGCGAGCCAAAGTCTAAGGACATCCTGAAGCTGCGCCTGTACGAGCAGCAGCACGGCAAGTGTCTGTATAGCGGCAAGGA
GATCAATCTGGTGCGGCTGAACGAGAAGGGCTATGTGGAGATCGATCACGCCCTGCCTTTCTCCAGAACCTGGGACGATTCTTTTA
ACAATAAGGTGCTGGTGCTGGGCAGCGAGAACCAGAATAAGGGCAATCAGACACCATACGAGTATTTCAATGGCAAGGACAACTC
CAGGGAGTGGCAGGAGTTCAAGGCCCGCGTGGAGACCTCTAGATTTCCCAGGAGCAAGAAGCAGCGGATCCTGCTGCAGAAGTT
CGACGAGGGCGGATCAGGAGGCTCTGGCGGTTCAGGTGGATCAGGCGGTAGCGGAGGTTCAGGTGGTTCTGAGGTGGAGTTTTC
CCACGAGTACTGGATGAGACATGCCCTGACCCTGGCCAAGAGGGCACGCGATGAGAGGGAGGTGCCTGTGGGAGCCGTGCTGGT
GCTGAACAATAGAGTGATCGGCGAGGGCTGGAACAGAGCCATCGGCCTGCACGACCCAACAGCCCATGCCGAAATTATGGCCCTG
AGACAGGGCGGCCTGGTCATGCAGAACTACAGACTGATTGACGCCACCCTGTACGTGACATTCGAGCCTTGCGTGATGTGCGCCG
GCGCCATGATCCACTCTAGGATCGGCCGCGTGGTGTTTGGCGTGAGGAACAGCAAACGGGGCGCCGCAGGCTCCCTGATGAACGT
GCTGAACTACCCCGGCATGAATCACCGCGTCGAAATTACCGAGGGAATCCTGGCAGATGAATGTGCCGCCCTGCTGTGCGACTTCT
ACCGGATGCCTAGACAGGTGTTCAATGCTCAGAAGAAGGCCCAGAGCTCCATCAACGGCTCCTCTGGCTCTGAGACACCTGGCACA
AGCGAGAGCGCAACACCTGAAAGCAGCGGCGATGGCTTTAAGGAGTGCAACCTGAATGACACCAGATACGTGAACCGGTTCCTGT
GCCAGTTTGTGGCCGATCACATCCTGCTGACCGGCAAGGGCAAGAGAAGGGTGTTCGCCTCTAATGGCCAGATCACAAACCTGCT
GAGGGGATTTTGGGGACTGAGGAAGGTGCGGGCAGAGAATGACAGACACCACGCACTGGATGCAGTGGTGGTGGCATGCAGCA
CCGTGGCAATGCAGCAGAAGATCACAAGATTCGTGAGGTATAAGGAGATGAACGCCTTTGACGGCAAGACCATCGATAAGGAGA
CAGGCAAGGTGCTGCACCAGAAGACCCACTTCCCCCAGCCTTGGGAGTTCTTTGCCCAGGAAGTGATGATCCGGGTGTTCGGCAA
GCCAGACGGCAAGCCTGAGTTTGAGGAGGCCGATACCCCAGAGAAGCTGAGGACACTGCTGGCAGAGAAGCTGTCTAGCAGGCC
AGAGGCAGTGCACGAGTACGTGACCCCACTGTTCGTGTCCAGGGCACCCAATCGGAAGATGTCTGGCGCCCACAAGGACACACTG
AGAAGCGCCAAGAGGTTTGTGAAGCACAACGAGAAGATCTCCGTGAAGAGAGTGTGGCTGACCGAGATCAAGCTGGCCGATCTG
GAGAACATGGTGAATTACAAGAACGGCAGGGAGATCGAGCTGTATGAGGCCCTGAAGGCAAGGCTGGAGGCCTACGGAGGAAA
TGCCAAGCAGGCCTTCGACCCAAAGGATAACCCCTTTTATAAGAAGGGAGGACAGCTGGTGAAGGCCGTGCGGGTGGAGAAGAC
CCAGGAGAGCGGCGTGCTGCTGAATAAGAAGAACGCCTACACAATCGCCGACAATGGCGATATGGTGAGAGTGGACGTGTTCTGT
AAGGTGGATAAGAAGGGCAAGAATCAGTACTTTATCGTGCCTATCTATGCCTGGCAGGTGGCCGAGAACATCCTGCCAGACATCG
ATTGCAAGGGCTACAGAATCGACGATAGCTATACATTCTGTTTTTCCCTGCACAAGTATGACCTGATCGCCTTCCAGAAGGATGAGA
AGTCCAAGGTGGAGTTTGCCTACTATATCAATTGCGACTCCTCTAACGGCAGGTTCTACCTGGCCTGGCACGATAAGGGCAGCAAG
GAGCAGCAGTTTCGCATCTCCACCCAGAATCTGGTGCTGATCCAGAAGTATCAGGTGAACGAGCTGGGCAAGGAGATCAGGCCAT
GTCGGCTGAAGAAGCGCCCACCCGTGCGGGAGGATAAAAGAACCGCCGACGGCAGCGAATTCGAGCCCAAGAAGAAGAGGAAA
GTC

Nme2-ABE8e-i6: BPSV40-NLS, Nme2Cas9, TadA8e, Linkers

AAACGGACAGCCGACGGAAGCGAGTTCGAGTCACCAAAGAAGAAGCGGAAAGTCGAAGATATGGCCGCCTTCAAGCCTAACCCA
ATCAATTACATCCTGGGACTGGCCATCGGAATCGCATCCGTGGGATGGGCTATGGTGGAGATCGACGAGGAGGAGAATCCTATCC
GGCTGATCGATCTGGGCGTGAGAGTGTTTGAGAGGGCCGAGGTGCCAAAGACCGGCGATTCTCTGGCTATGGCCCGGAGACTGG
CACGGAGCGTGAGGCGCCTGACACGGAGAAGGGCACACAGGCTGCTGAGGGCACGCCGGCTGCTGAAGAGAGAGGGCGTGCTG
CAGGCAGCAGACTTCGATGAGAATGGCCTGATCAAGAGCCTGCCAAACACCCCCTGGCAGCTGAGAGCAGCCGCCCTGGACAGGA
AGCTGACACCACTGGAGTGGTCTGCCGTGCTGCTGCACCTGATCAAGCACCGCGGCTACCTGAGCCAGCGGAAGAACGAGGGAG
AGACAGCAGACAAGGAGCTGGGCGCCCTGCTGAAGGGAGTGGCCAACAATGCCCACGCCCTGCAGACCGGCGATTTCAGGACAC
CTGCCGAGCTGGCCCTGAATAAGTTTGAGAAGGAGTCCGGCCACATCAGAAACCAGAGGGGCGACTATAGCCACACCTTCTCCCG
CAAGGATCTGCAGGCCGAGCTGATCCTGCTGTTCGAGAAGCAGAAGGAGTTTGGCAATCCACACGTGAGCGGAGGCCTGAAGGA
GGGAATCGAGACCCTGCTGATGACACAGAGGCCTGCCCTGTCCGGCGACGCAGTGCAGAAGATGCTGGGACACTGCACCTTCGAG
CCTGCAGAGCCAAAGGCCGCCAAGAACACCTACACAGCCGAGCGGTTTATCTGGCTGACAAAGCTGAACAATCTGAGAATCCTGG
AGCAGGGATCCGAGAGGCCACTGACCGACACAGAGAGGGCCACCCTGATGGATGAGCCTTACCGGAAGTCTAAGCTGACATATG
CCCAGGCCAGAAAGCTGCTGGGCCTGGAGGACACCGCCTTCTTTAAGGGCCTGAGATACGGCAAGGATAATGCCGAGGCCTCCAC
ACTGATGGAGATGAAGGCCTATCACGCCATCTCTCGCGCCCTGGAGAAGGAGGGCCTGAAGGACAAGAAGTCCCCCCTGAACCTG
AGCTCCGAGCTGCAGGATGAGATCGGCACCGCCTTCTCTCTGTTTAAGACCGACGAGGATATCACAGGCCGCCTGAAGGACAGGG
TGCAGCCTGAGATCCTGGAGGCCCTGCTGAAGCACATCTCTTTCGATAAGTTTGTGCAGATCAGCCTGAAGGCCCTGAGAAGGATC
GTGCCACTGATGGAGCAGGGCAAGCGGTACGACGAGGCCTGCGCCGAGATCTACGGCGATCACTATGGCAAGAAGAACACAGAG
GAGAAGATCTATCTGCCCCCTATCCCTGCCGACGAGATCAGAAATCCTGTGGTGCTGAGGGCCCTGTCCCAGGCAAGAAAAGTGAT
CAACGGAGTGGTGCGCCGGTACGGATCTCCAGCCCGGATCCACATCGAGACCGCCAGAGAAGTGGGCAAGAGCTTCAAGGACCG
GAAGGAGATCGAGAAGAGACAGGAGGAGAATCGCAAGGATCGGGAGAAGGCCGCCGCCAAGTTTAGGGAGTACTTCCCTAACTT
TGTGGGCGAGCCAAAGTCTAAGGACATCCTGAAGCTGCGCCTGTACGAGCAGCAGCACGGCAAGTGTCTGTATAGCGGCAAGGA
GATCAATCTGGTGCGGCTGAACGAGAAGGGCTATGTGGAGATCGATCACGCCCTGCCTTTCTCCAGAACCTGGGACGATTCTTTTA
ACAATAAGGTGCTGGTGCTGGGCAGCGAGAACCAGAATAAGGGCAATCAGACACCATACGAGTATTTCAATGGCAAGGACAACTC
CAGGGAGTGGCAGGAGTTCAAGGCCCGCGTGGAGACCTCTAGATTTCCCAGGAGCAAGAAGCAGCGGATCCTGCTGCAGAAGTT
CGACGAGGATGGCTTTAAGGAGTGCAACCTGAATGACACCAGATACGTGAACCGGTTCCTGTGCCAGTTTGTGGCCGATCACATCC
TGCTGACCGGCAAGGGCAAGAGAAGGGTGTTCGCCTCTAATGGCCAGATCACAAACCTGCTGAGGGGATTTTGGGGACTGAGGA
AGGTGGGCGGATCAGGAGGCTCTGGCGGTTCAGGTGGATCAGGCGGTAGCGGAGGTTCAGGTGGTTCTGAGGTGGAGTTTTCCC
ACGAGTACTGGATGAGACATGCCCTGACCCTGGCCAAGAGGGCACGCGATGAGAGGGAGGTGCCTGTGGGAGCCGTGCTGGTGC
TGAACAATAGAGTGATCGGCGAGGGCTGGAACAGAGCCATCGGCCTGCACGACCCAACAGCCCATGCCGAAATTATGGCCCTGAG
ACAGGGCGGCCTGGTCATGCAGAACTACAGACTGATTGACGCCACCCTGTACGTGACATTCGAGCCTTGCGTGATGTGCGCCGGC
GCCATGATCCACTCTAGGATCGGCCGCGTGGTGTTTGGCGTGAGGAACAGCAAACGGGGCGCCGCAGGCTCCCTGATGAACGTGC
TGAACTACCCCGGCATGAATCACCGCGTCGAAATTACCGAGGGAATCCTGGCAGATGAATGTGCCGCCCTGCTGTGCGACTTCTAC
CGGATGCCTAGACAGGTGTTCAATGCTCAGAAGAAGGCCCAGAGCTCCATCAACGGCTCCTCTGGCTCTGAGACACCTGGCACAA
GCGAGAGCGCAACACCTGAAAGCAGCGGCCGGGCAGAGAATGACAGACACCACGCACTGGATGCAGTGGTGGTGGCATGCAGC
ACCGTGGCAATGCAGCAGAAGATCACAAGATTCGTGAGGTATAAGGAGATGAACGCCTTTGACGGCAAGACCATCGATAAGGAG
ACAGGCAAGGTGCTGCACCAGAAGACCCACTTCCCCCAGCCTTGGGAGTTCTTTGCCCAGGAAGTGATGATCCGGGTGTTCGGCAA
GCCAGACGGCAAGCCTGAGTTTGAGGAGGCCGATACCCCAGAGAAGCTGAGGACACTGCTGGCAGAGAAGCTGTCTAGCAGGCC
AGAGGCAGTGCACGAGTACGTGACCCCACTGTTCGTGTCCAGGGCACCCAATCGGAAGATGTCTGGCGCCCACAAGGACACACTG
AGAAGCGCCAAGAGGTTTGTGAAGCACAACGAGAAGATCTCCGTGAAGAGAGTGTGGCTGACCGAGATCAAGCTGGCCGATCTG
GAGAACATGGTGAATTACAAGAACGGCAGGGAGATCGAGCTGTATGAGGCCCTGAAGGCAAGGCTGGAGGCCTACGGAGGAAA
TGCCAAGCAGGCCTTCGACCCAAAGGATAACCCCTTTTATAAGAAGGGAGGACAGCTGGTGAAGGCCGTGCGGGTGGAGAAGAC
CCAGGAGAGCGGCGTGCTGCTGAATAAGAAGAACGCCTACACAATCGCCGACAATGGCGATATGGTGAGAGTGGACGTGTTCTGT
AAGGTGGATAAGAAGGGCAAGAATCAGTACTTTATCGTGCCTATCTATGCCTGGCAGGTGGCCGAGAACATCCTGCCAGACATCG
ATTGCAAGGGCTACAGAATCGACGATAGCTATACATTCTGTTTTTCCCTGCACAAGTATGACCTGATCGCCTTCCAGAAGGATGAGA
AGTCCAAGGTGGAGTTTGCCTACTATATCAATTGCGACTCCTCTAACGGCAGGTTCTACCTGGCCTGGCACGATAAGGGCAGCAAG
GAGCAGCAGTTTCGCATCTCCACCCAGAATCTGGTGCTGATCCAGAAGTATCAGGTGAACGAGCTGGGCAAGGAGATCAGGCCAT
GTCGGCTGAAGAAGCGCCCACCCGTGCGGGAGGATAAAAGAACCGCCGACGGCAGCGAATTCGAGCCCAAGAAGAAGAGGAAA
GTC

Nme2-ABE8e-i7: BPSV40-NLS, Nme2Cas9, TadA8e, Linkers

AAACGGACAGCCGACGGAAGCGAGTTCGAGTCACCAAAGAAGAAGCGGAAAGTCGAAGATATGGCCGCCTTCAAGCCTAACCCA
ATCAATTACATCCTGGGACTGGCCATCGGAATCGCATCCGTGGGATGGGCTATGGTGGAGATCGACGAGGAGGAGAATCCTATCC
GGCTGATCGATCTGGGCGTGAGAGTGTTTGAGAGGGCCGAGGTGCCAAAGACCGGCGATTCTCTGGCTATGGCCCGGAGACTGG
CACGGAGCGTGAGGCGCCTGACACGGAGAAGGGCACACAGGCTGCTGAGGGCACGCCGGCTGCTGAAGAGAGAGGGCGTGCTG
CAGGCAGCAGACTTCGATGAGAATGGCCTGATCAAGAGCCTGCCAAACACCCCCTGGCAGCTGAGAGCAGCCGCCCTGGACAGGA
AGCTGACACCACTGGAGTGGTCTGCCGTGCTGCTGCACCTGATCAAGCACCGCGGCTACCTGAGCCAGCGGAAGAACGAGGGAG
AGACAGCAGACAAGGAGCTGGGCGCCCTGCTGAAGGGAGTGGCCAACAATGCCCACGCCCTGCAGACCGGCGATTTCAGGACAC
CTGCCGAGCTGGCCCTGAATAAGTTTGAGAAGGAGTCCGGCCACATCAGAAACCAGAGGGGCGACTATAGCCACACCTTCTCCCG
CAAGGATCTGCAGGCCGAGCTGATCCTGCTGTTCGAGAAGCAGAAGGAGTTTGGCAATCCACACGTGAGCGGAGGCCTGAAGGA
GGGAATCGAGACCCTGCTGATGACACAGAGGCCTGCCCTGTCCGGCGACGCAGTGCAGAAGATGCTGGGACACTGCACCTTCGAG
CCTGCAGAGCCAAAGGCCGCCAAGAACACCTACACAGCCGAGCGGTTTATCTGGCTGACAAAGCTGAACAATCTGAGAATCCTGG
AGCAGGGATCCGAGAGGCCACTGACCGACACAGAGAGGGCCACCCTGATGGATGAGCCTTACCGGAAGTCTAAGCTGACATATG
CCCAGGCCAGAAAGCTGCTGGGCCTGGAGGACACCGCCTTCTTTAAGGGCCTGAGATACGGCAAGGATAATGCCGAGGCCTCCAC
ACTGATGGAGATGAAGGCCTATCACGCCATCTCTCGCGCCCTGGAGAAGGAGGGCCTGAAGGACAAGAAGTCCCCCCTGAACCTG
AGCTCCGAGCTGCAGGATGAGATCGGCACCGCCTTCTCTCTGTTTAAGACCGACGAGGATATCACAGGCCGCCTGAAGGACAGGG
TGCAGCCTGAGATCCTGGAGGCCCTGCTGAAGCACATCTCTTTCGATAAGTTTGTGCAGATCAGCCTGAAGGCCCTGAGAAGGATC
GTGCCACTGATGGAGCAGGGCAAGCGGTACGACGAGGCCTGCGCCGAGATCTACGGCGATCACTATGGCAAGAAGAACACAGAG
GAGAAGATCTATCTGCCCCCTATCCCTGCCGACGAGATCAGAAATCCTGTGGTGCTGAGGGCCCTGTCCCAGGCAAGAAAAGTGAT
CAACGGAGTGGTGCGCCGGTACGGATCTCCAGCCCGGATCCACATCGAGACCGCCAGAGAAGTGGGCAAGAGCTTCAAGGACCG
GAAGGAGATCGAGAAGAGACAGGAGGAGAATCGCAAGGATCGGGAGAAGGCCGCCGCCAAGTTTAGGGAGTACTTCCCTAACTT
TGTGGGCGAGCCAAAGTCTAAGGACATCCTGAAGCTGCGCCTGTACGAGCAGCAGCACGGCAAGTGTCTGTATAGCGGCAAGGA
GATCAATCTGGTGCGGCTGAACGAGAAGGGCTATGTGGAGATCGATCACGCCCTGCCTTTCTCCAGAACCTGGGACGATTCTTTA
ACAATAAGGTGCTGGTGCTGGGCAGCGAGAACCAGAATAAGGGCAATCAGACACCATACGAGTATTTCAATGGCAAGGACAACTC
CAGGGAGTGGCAGGAGTTCAAGGCCCGCGTGGAGACCTCTAGATTTCCCAGGAGCAAGAAGCAGCGGATCCTGCTGCAGAAGTT
CGACGAGGATGGCTTTAAGGAGTGCAACCTGAATGACACCAGATACGTGAACCGGTTCCTGTGCCAGTTTGTGGCCGATCACATCC
TGCTGACCGGCAAGGGCAAGAGAAGGGTGTTCGCCTCTAATGGCCAGATCACAAACCTGCTGAGGGGATTTTGGGGACTGAGGA
AGGTGCGGGCAGAGAATGACAGACACCACGCACTGGATGCAGTGGTGGTGGCATGCAGCACCGTGGCAATGCAGCAGAAGATCA
CAAGATTCGTGAGGTATAAGGAGATGAACGCCTTTGACGGCAAGACCATCGATAAGGAGGGCGGATCAGGAGGCTCTGGCGGTT
CAGGTGGATCAGGCGGTAGCGGAGGTTCAGGTGGTTCTGAGGTGGAGTTTTCCCACGAGTACTGGATGAGACATGCCCTGACCCT
GGCCAAGAGGGCACGCGATGAGAGGGAGGTGCCTGTGGGAGCCGTGCTGGTGCTGAACAATAGAGTGATCGGCGAGGGCTGGA
ACAGAGCCATCGGCCTGCACGACCCAACAGCCCATGCCGAAATTATGGCCCTGAGACAGGGCGGCCTGGTCATGCAGAACTACAG
ACTGATTGACGCCACCCTGTACGTGACATTCGAGCCTTGCGTGATGTGCGCCGGCGCCATGATCCACTCTAGGATCGGCCGCGTGG
TGTTTGGCGTGAGGAACAGCAAACGGGGCGCCGCAGGCTCCCTGATGAACGTGCTGAACTACCCCGGCATGAATCACCGCGTCGA
AATTACCGAGGGAATCCTGGCAGATGAATGTGCCGCCCTGCTGTGCGACTTCTACCGGATGCCTAGACAGGTGTTCAATGCTCAGA
AGAAGGCCCAGAGCTCCATCAACGGCTCCTCTGGCTCTGAGACACCTGGCACAAGCGAGAGCGCAACACCTGAAAGCAGCGGCAC
AGGCAAGGTGCTGCACCAGAAGACCCACTTCCCCCAGCCTTGGGAGTTCTTTGCCCAGGAAGTGATGATCCGGGTGTTCGGCAAG
CCAGACGGCAAGCCTGAGTTTGAGGAGGCCGATACCCCAGAGAAGCTGAGGACACTGCTGGCAGAGAAGCTGTCTAGCAGGCCA
GAGGCAGTGCACGAGTACGTGACCCCACTGTTCGTGTCCAGGGCACCCAATCGGAAGATGTCTGGCGCCCACAAGGACACACTGA
GAAGCGCCAAGAGGTTTGTGAAGCACAACGAGAAGATCTCCGTGAAGAGAGTGTGGCTGACCGAGATCAAGCTGGCCGATCTGG
AGAACATGGTGAATTACAAGAACGGCAGGGAGATCGAGCTGTATGAGGCCCTGAAGGCAAGGCTGGAGGCCTACGGAGGAAAT
GCCAAGCAGGCCTTCGACCCAAAGGATAACCCCTTTTATAAGAAGGGAGGACAGCTGGTGAAGGCCGTGCGGGTGGAGAAGACC
CAGGAGAGCGGCGTGCTGCTGAATAAGAAGAACGCCTACACAATCGCCGACAATGGCGATATGGTGAGAGTGGACGTGTTCTGT
AAGGTGGATAAGAAGGGCAAGAATCAGTACTTTATCGTGCCTATCTATGCCTGGCAGGTGGCCGAGAACATCCTGCCAGACATCG
ATTGCAAGGGCTACAGAATCGACGATAGCTATACATTCTGTTTTTCCCTGCACAAGTATGACCTGATCGCCTTCCAGAAGGATGAGA
AGTCCAAGGTGGAGTTTGCCTACTATATCAATTGCGACTCCTCTAACGGCAGGTTCTACCTGGCCTGGCACGATAAGGGCAGCAAG
GAGCAGCAGTTTCGCATCTCCACCCAGAATCTGGTGCTGATCCAGAAGTATCAGGTGAACGAGCTGGGCAAGGAGATCAGGCCAT
GTCGGCTGAAGAAGCGCCCACCCGTGCGGGAGGATAAAAGAACCGCCGACGGCAGCGAATTCGAGCCCAAGAAGAAGAGGAAA
GTC

Nme2-ABE8e-i8: BPSV40-NLS, Nme2Cas9, TadA8e, Linkers

AAACGGACAGCCGACGGAAGCGAGTTCGAGTCACCAAAGAAGAAGCGGAAAGTCGAAGATATGGCCGCCTTCAAGCCTAACCCA
ATCAATTACATCCTGGGACTGGCCATCGGAATCGCATCCGTGGGATGGGCTATGGTGGAGATCGACGAGGAGGAGAATCCTATCC
GGCTGATCGATCTGGGCGTGAGAGTGTTTGAGAGGGCCGAGGTGCCAAAGACCGGCGATTCTCTGGCTATGGCCCGGAGACTGG
CACGGAGCGTGAGGCGCCTGACACGGAGAAGGGCACACAGGCTGCTGAGGGCACGCCGGCTGCTGAAGAGAGAGGGCGTGCTG
CAGGCAGCAGACTTCGATGAGAATGGCCTGATCAAGAGCCTGCCAAACACCCCCTGGCAGCTGAGAGCAGCCGCCCTGGACAGGA
AGCTGACACCACTGGAGTGGTCTGCCGTGCTGCTGCACCTGATCAAGCACCGCGGCTACCTGAGCCAGCGGAAGAACGAGGGAG
AGACAGCAGACAAGGAGCTGGGCGCCCTGCTGAAGGGAGTGGCCAACAATGCCCACGCCCTGCAGACCGGCGATTTCAGGACAC
CTGCCGAGCTGGCCCTGAATAAGTTTGAGAAGGAGTCCGGCCACATCAGAAACCAGAGGGGCGACTATAGCCACACCTTCTCCCG
CAAGGATCTGCAGGCCGAGCTGATCCTGCTGTTCGAGAAGCAGAAGGAGTTTGGCAATCCACACGTGAGCGGAGGCCTGAAGGA
GGGAATCGAGACCCTGCTGATGACACAGAGGCCTGCCCTGTCCGGCGACGCAGTGCAGAAGATGCTGGGACACTGCACCTTCGAG
CCTGCAGAGCCAAAGGCCGCCAAGAACACCTACACAGCCGAGCGGTTTATCTGGCTGACAAAGCTGAACAATCTGAGAATCCTGG
AGCAGGGATCCGAGAGGCCACTGACCGACACAGAGAGGGCCACCCTGATGGATGAGCCTTACCGGAAGTCTAAGCTGACATATG
CCCAGGCCAGAAAGCTGCTGGGCCTGGAGGACACCGCCTTCTTTAAGGGCCTGAGATACGGCAAGGATAATGCCGAGGCCTCCAC
ACTGATGGAGATGAAGGCCTATCACGCCATCTCTCGCGCCCTGGAGAAGGAGGGCCTGAAGGACAAGAAGTCCCCCCTGAACCTG
AGCTCCGAGCTGCAGGATGAGATCGGCACCGCCTTCTCTCTGTTTAAGACCGACGAGGATATCACAGGCCGCCTGAAGGACAGGG
TGCAGCCTGAGATCCTGGAGGCCCTGCTGAAGCACATCTCTTTCGATAAGTTTGTGCAGATCAGCCTGAAGGCCCTGAGAAGGATC
GTGCCACTGATGGAGCAGGGCAAGCGGTACGACGAGGCCTGCGCCGAGATCTACGGCGATCACTATGGCAAGAAGAACACAGAG
GAGAAGATCTATCTGCCCCCTATCCCTGCCGACGAGATCAGAAATCCTGTGGTGCTGAGGGCCCTGTCCCAGGCAAGAAAAGTGAT
CAACGGAGTGGTGCGCCGGTACGGATCTCCAGCCCGGATCCACATCGAGACCGCCAGAGAAGTGGGCAAGAGCTTCAAGGACCG
GAAGGAGATCGAGAAGAGACAGGAGGAGAATCGCAAGGATCGGGAGAAGGCCGCCGCCAAGTTTAGGGAGTACTTCCCTAACTT
TGTGGGCGAGCCAAAGTCTAAGGACATCCTGAAGCTGCGCCTGTACGAGCAGCAGCACGGCAAGTGTCTGTATAGCGGCAAGGA
GATCAATCTGGTGCGGCTGAACGAGAAGGGCTATGTGGAGATCGATCACGCCCTGCCTTTCTCCAGAACCTGGGACGATTCTTTTA
ACAATAAGGTGCTGGTGCTGGGCAGCGAGAACCAGAATAAGGGCAATCAGACACCATACGAGTATTTCAATGGCAAGGACAACTC
CAGGGAGTGGCAGGAGTTCAAGGCCCGCGTGGAGACCTCTAGATTTCCCAGGAGCAAGAAGCAGCGGATCCTGCTGCAGAAGTT
CGACGAGGATGGCTTTAAGGAGTGCAACCTGAATGACACCAGATACGTGAACCGGTTCCTGTGCCAGTTTGTGGCCGATCACATCC
TGCTGACCGGCAAGGGCAAGAGAAGGGTGTTCGCCTCTAATGGCCAGATCACAAACCTGCTGAGGGGATTTTGGGGACTGAGGA
AGGTGCGGGCAGAGAATGACAGACACCACGCACTGGATGCAGTGGTGGTGGCATGCAGCACCGTGGCAATGCAGCAGAAGATCA
CAAGATTCGTGAGGTATAAGGAGATGAACGCCTTTGACGGCAAGACCATCGATAAGGAGACAGGCAAGGTGCTGCACCAGAAGA
CCCACTTCCCCCAGCCTTGGGAGTTCTTTGCCCAGGAAGTGATGATCCGGGTGTTCGGCAAGCCAGACGGCAAGCCTGGCGGATCA
GGAGGCTCTGGCGGTTCAGGTGGATCAGGCGGTAGCGGAGGTTCAGGTGGTTCTGAGGTGGAGTTTTCCCACGAGTACTGGATG
AGACATGCCCTGACCCTGGCCAAGAGGGCACGCGATGAGAGGGAGGTGCCTGTGGGAGCCGTGCTGGTGCTGAACAATAGAGTG
ATCGGCGAGGGCTGGAACAGAGCCATCGGCCTGCACGACCCAACAGCCCATGCCGAAATTATGGCCCTGAGACAGGGCGGCCTG
GTCATGCAGAACTACAGACTGATTGACGCCACCCTGTACGTGACATTCGAGCCTTGCGTGATGTGCGCCGGCGCCATGATCCACTC
TAGGATCGGCCGCGTGGTGTTTGGCGTGAGGAACAGCAAACGGGGCGCCGCAGGCTCCCTGATGAACGTGCTGAACTACCCCGG
CATGAATCACCGCGTCGAAATTACCGAGGGAATCCTGGCAGATGAATGTGCCGCCCTGCTGTGCGACTTCTACCGGATGCCTAGAC
AGGTGTTCAATGCTCAGAAGAAGGCCCAGAGCTCCATCAACGGCTCCTCTGGCTCTGAGACACCTGGCACAAGCGAGAGCGCAAC
ACCTGAAAGCAGCGGCGAGTTTGAGGAGGCCGATACCCCAGAGAAGCTGAGGACACTGCTGGCAGAGAAGCTGTCTAGCAGGCC
AGAGGCAGTGCACGAGTACGTGACCCCACTGTTCGTGTCCAGGGCACCCAATCGGAAGATGTCTGGCGCCCACAAGGACACACTG
AGAAGCGCCAAGAGGTTTGTGAAGCACAACGAGAAGATCTCCGTGAAGAGAGTGTGGCTGACCGAGATCAAGCTGGCCGATCTG
GAGAACATGGTGAATTACAAGAACGGCAGGGAGATCGAGCTGTATGAGGCCCTGAAGGCAAGGCTGGAGGCCTACGGAGGAAA
TGCCAAGCAGGCCTTCGACCCAAAGGATAACCCCTTTTATAAGAAGGGAGGACAGCTGGTGAAGGCCGTGCGGGTGGAGAAGAC
CCAGGAGAGCGGCGTGCTGCTGAATAAGAAGAACGCCTACACAATCGCCGACAATGGCGATATGGTGAGAGTGGACGTGTTCTGT
AAGGTGGATAAGAAGGGCAAGAATCAGTACTTTATCGTGCCTATCTATGCCTGGCAGGTGGCCGAGAACATCCTGCCAGACATCG
ATTGCAAGGGCTACAGAATCGACGATAGCTATACATTCTGTTTTTCCCTGCACAAGTATGACCTGATCGCCTTCCAGAAGGATGAGA
AGTCCAAGGTGGAGTTTGCCTACTATATCAATTGCGACTCCTCTAACGGCAGGTTCTACCTGGCCTGGCACGATAAGGGCAGCAAG
GAGCAGCAGTTTCGCATCTCCACCCAGAATCTGGTGCTGATCCAGAAGTATCAGGTGAACGAGCTGGGCAAGGAGATCAGGCCAT
GTCGGCTGAAGAAGCGCCCACCCGTGCGGGAGGATAAAAGAACCGCCGACGGCAGCGAATTCGAGCCCAAGAAGAAGAGGAAA
GTC

Nme2^{smu}-ABE8e-nt: BPSV40-NLS, Nme2Cas9 – delta PID, TadA8e, SmuCas9 PID, Linkers

AAACGGACAGCCGACGGAAGCGAGTTCGAGTCACCAAAGAAGAAGCGGAAAGTCGGCGGTAGCGGCGGAGGCAGCGGTGGCG
GCAGCGGCTCTGAGGTGGAGTTTTCCCACGAGTACTGGATGAGACATGCCCTGACCCTGGCCAAGAGGGCACGCGATGAGAGGG
AGGTGCCTGTGGGAGCCGTGCTGGTGCTGAACAATAGAGTGATCGGCGAGGGCTGGAACAGAGCCATCGGCCTGCACGACCCAA
CAGCCCATGCCGAAATTATGGCCCTGAGACAGGGCGGCCTGGTCATGCAGAACTACAGACTGATTGACGCCACCCTGTACGTGAC
ATTCGAGCCTTGCGTGATGTGCGCCGGCGCCATGATCCACTCTAGGATCGGCCGCGTGGTGTTTGGCGTGAGGAACAGCAAACGG
GGCGCCGCAGGCTCCCTGATGAACGTGCTGAACTACCCCGGCATGAATCACCGCGTCGAAATTACCGAGGGAATCCTGGCAGATG
AATGTGCCGCCCTGCTGTGCGACTTCTACCGGATGCCTAGACAGGTGTTCAATGCTCAGAAGAAGGCCCAGAGCTCCATCAACTCC
GGAGGATCTAGCGGAGGCTCCTCTGGCTCTGAGACACCTGGCACAAGCGAGAGCGCAACACCTGAAAGCAGCGGGGGCAGCAGC
GGGGGGTCAATGGCCGCCTTCAAGCCTAACCCAATCAATTACATCCTGGGACTGGCCATCGGAATCGCATCCGTGGGATGGGCTAT
GGTGGAGATCGACGAGGAGGAGAATCCTATCCGGCTGATCGATCTGGGCGTGAGAGTGTTTGAGAGGGCCGAGGTGCCAAAGAC
CGGCGATTCTCTGGCTATGGCCCGGAGACTGGCACGGAGCGTGAGGCGCCTGACACGGAGAAGGGCACACAGGCTGCTGAGGGC
ACGCCGGCTGCTGAAGAGAGAGGGCGTGCTGCAGGCAGCAGACTTCGATGAGAATGGCCTGATCAAGAGCCTGCCAAACACCCC
CTGGCAGCTGAGAGCAGCCGCCCTGGACAGGAAGCTGACACCACTGGAGTGGTCTGCCGTGCTGCTGCACCTGATCAAGCACCGC
GGCTACCTGAGCCAGCGGAAGAACGAGGGAGAGACAGCAGACAAGGAGCTGGGCGCCCTGCTGAAGGGAGTGGCCAACAATGC
CCACGCCCTGCAGACCGGCGATTTCAGGACACCTGCCGAGCTGGCCCTGAATAAGTTTGAGAAGGAGTCCGGCCACATCAGAAAC
CAGAGGGGCGACTATAGCCACACCTTCTCCCGCAAGGATCTGCAGGCCGAGCTGATCCTGCTGTTCGAGAAGCAGAAGGAGTTTG
GCAATCCACACGTGAGCGGAGGCCTGAAGGAGGGAATCGAGACCCTGCTGATGACACAGAGGCCTGCCCTGTCCGGCGACGCAG
TGCAGAAGATGCTGGGACACTGCACCTTCGAGCCTGCAGAGCCAAAGGCCGCCAAGAACACCTACACAGCCGAGCGGTTTATCTG
GCTGACAAAGCTGAACAATCTGAGAATCCTGGAGCAGGGATCCGAGAGGCCACTGACCGACACAGAGAGGGCCACCCTGATGGA
TGAGCCTTACCGGAAGTCTAAGCTGACATATGCCCAGGCCAGAAAGCTGCTGGGCCTGGAGGACACCGCCTTCTTTAAGGGCCTG
AGATACGGCAAGGATAATGCCGAGGCCTCCACACTGATGGAGATGAAGGCCTATCACGCCATCTCTCGCGCCCTGGAGAAGGAG
GCCTGAAGGACAAGAAGTCCCCCCTGAACCTGAGCTCCGAGCTGCAGGATGAGATCGGCACCGCCTTCTCTCTGTTTAAGACCGAC
GAGGATATCACAGGCCGCCTGAAGGACAGGGTGCAGCCTGAGATCCTGGAGGCCCTGCTGAAGCACATCTCTTTCGATAAGTTTG
TGCAGATCAGCCTGAAGGCCCTGAGAAGGATCGTGCCACTGATGGAGCAGGGCAAGCGGTACGACGAGGCCTGCGCCGAGATCT
ACGGCGATCACTATGGCAAGAAGAACACAGAGGAGAAGATCTATCTGCCCCCTATCCCTGCCGACGAGATCAGAAATCCTGTGGT
GCTGAGGGCCCTGTCCCAGGCAAGAAAAGTGATCAACGGAGTGGTGCGCCGGTACGGATCTCCAGCCCGGATCCACATCGAGACC
GCCAGAGAAGTGGGCAAGAGCTTCAAGGACCGGAAGGAGATCGAGAAGAGACAGGAGGAGAATCGCAAGGATCGGGAGAAGG
CCGCCGCCAAGTTTAGGGAGTACTTCCCTAACTTTGTGGGCGAGCCAAAGTCTAAGGACATCCTGAAGCTGCGCCTGTACGAGCAG
CAGCACGGCAAGTGTCTGTATAGCGGCAAGGAGATCAATCTGGTGCGGCTGAACGAGAAGGGCTATGTGGAGATCGATCACGCC
CTGCCTTTCTCCAGAACCTGGGACGATTCTTTTAACAATAAGGTGCTGGTGCTGGGCAGCGAGAACCAGAATAAGGGCAATCAGAC
ACCATACGAGTATTTCAATGGCAAGGACAACTCCAGGGAGTGGCAGGAGTTCAAGGCCCGCGTGGAGACCTCTAGATTTCCCAGG
AGCAAGAAGCAGCGGATCCTGCTGCAGAAGTTCGACGAGGATGGCTTTAAGGAGTGCAACCTGAATGACACCAGATACGTGAACC
GGTTCCTGTGCCAGTTTGTGGCCGATCACATCCTGCTGACCGGCAAGGGCAAGAGAAGGGTGTTCGCCTCTAATGGCCAGATCACA
AACCTGCTGAGGGGATTTTGGGGACTGAGGAAGGTGCGGGCAGAGAATGACAGACACCACGCACTGGATGCAGTGGTGGTGGC
ATGCAGCACCGTGGCAATGCAGCAGAAGATCACAAGATTCGTGAGGTATAAGGAGATGAACGCCTTTGACGGCAAGACCATCGAT
AAGGAGACAGGCAAGGTGCTGCACCAGAAGACCCACTTCCCCCAGCCTTGGGAGTTCTTTGCCCAGGAAGTGATGATCCGGGTGT
TCGGCAAGCCAGACGGCAAGCCTGAGTTTGAGGAGGCCGATACCCCAGAGAAGCTGAGGACACTGCTGGCAGAGAAGCTGTCTA
GCAGGCCAGAGGCAGTGCACGAGTACGTGACCCCACTGTTCGTGTCCAGGGCACCCAATCGGAAGATGTCTGGCGCCCACAAGGA
CACACTGAGAAGCGCCAAGAGGTTTGTGAAGCACAACGAGAAGATCTCCGTGAAGAGAGTGTGGCTGACCGAGATCAAGCTGGC
CGATCTGGAGAACATGGTGAATTACAAGAACGGCAGGGAGATCGAGCTGTATGAGGCCCTGAAGGCAAGGCTGGAGGCCTACGG
AGGAAATGCCAAGCAGGCCTTCGACCCAAAGGATAACCCCTTTTATAAGAAGGGAGGACAGCTGGTGAAGGCCGTGCGGGTGGA
GAAGACCCAGGAGAGCGGCGTGCTGCTGAATAAGAAGAACGCCTACACAATCGCCGACAACGCCACCATGGTGCGGGTGGACGT
GTACACCAAGGCCGGCAAGAACTACCTGGTTCCTGTGTACGTGTGGCAGGTGGCCCAGGGCATCTTACCCAACCGCGCCGTGACC
AGCGGCAAGTCCGAGGCTGACTGGGACCTGATCGATGAGAGCTTCGAGTTCAAGTTCTCTCTGTCCCGGGGAGATCTCGTGGAAA
TGATCTCCAACAAGGGCAGAATCTTCGGCTACTACAACGGCCTGGACAGAGCCAACGGCTCTATTGGAATTAGAGAGCACGACCTA
GAGAAGAGCAAGGGCAAAGACGGCGTGCATAGAGTGGGAGTGAAAACAGCTACAGCATTTAACAAGTACCACGTGGATCCCCTG
GGCAAAGAGATCCACAGATGCAGCAGCGAACCCAGACCTACACTGAAAATCAAGTCTAAGAAGGAGGATAAAAGAACCGCCGAC
GGCAGCGAATTCGAGCCCAAGAAGAAGAGGAAAGTC

Nme2^{Smu}-ABE8e-i1: BPSV40-NLS, Nme2Cas9 – delta PID, TadA8e, SmuCas9 PID, Linkers

AAACGGACAGCCGACGGAAGCGAGTTCGAGTCACCAAAGAAGAAGCGGAAAGTCGAAGATATGGCCGCCTTCAAGCCTAACCCA
ATCAATTACATCCTGGGACTGGCCATCGGAATCGCATCCGTGGGATGGGCTATGGTGGAGATCGACGAGGAGGAGAATCCTATCC
GGCTGATCGATCTGGGCGTGAGAGTGTTTGAGAGGGCCGAGGTGCCAAAGACCGGCGATTCTCTGGCTATGGCCCGGAGACTGG
CACGGAGCGTGAGGCGCCTGACACGGAGAAGGGCACACAGGCTGCTGAGGGCACGCCGGCTGCTGAAGAGAGAGGGCGTGCTG
CAGGCAGCAGACTTCGATGAGAATGGCCTGATCAAGAGCCTGCCAAACACCCCCTGGCAGCTGAGAGCAGCCGCCCTGGACAGGA
AGCTGACACCACTGGAGTGGTCTGCCGTGCTGCTGCACCTGATCAAGCACCGCGGCTACCTGAGCCAGCGGAAGAACGAGGGAG
AGACAGCAGACAAGGAGCTGGGCGCCCTGCTGAAGGGAGTGGCCAACAATGCCCACGCCCTGCAGACCGGCGATTTCAGGACAC
CTGCCGAGCTGGCCCTGAATAAGTTTGAGAAGGAGTCCGGCCACATCAGAAACCAGAGGGGCGACTATAGCCACACCTTCTCCCG
CAAGGATCTGCAGGCCGAGCTGATCCTGCTGTTCGAGAAGCAGAAGGAGTTTGGCAATCCACACGTGAGCGGAGGCCTGAAGGA
GGGAATCGAGACCCTGCTGATGACACAGAGGCCTGCCCTGTCCGGCGACGCAGTGCAGAAGATGCTGGGACACTGCACCTTCGAG
CCTGCAGAGCCAAAGGCCGCCAAGAACACCTACACAGCCGAGCGGTTTATCTGGCTGACAAAGCTGAACAATCTGAGAATCCTGG
AGCAGGGCGGATCAGGAGGCTCTGGCGGTTCAGGTGGATCAGGCGGTAGCGGAGGTTCAGGTGGTTCTGAGGTGGAGTTTTCCC
ACGAGTACTGGATGAGACATGCCCTGACCCTGGCCAAGAGGGCACGCGATGAGAGGGAGGTGCCTGTGGGAGCCGTGCTGGTGC
TGAACAATAGAGTGATCGGCGAGGGCTGGAACAGAGCCATCGGCCTGCACGACCCAACAGCCCATGCCGAAATTATGGCCCTGAG
ACAGGGCGGCCTGGTCATGCAGAACTACAGACTGATTGACGCCACCCTGTACGTGACATTCGAGCCTTGCGTGATGTGCGCCGGC
GCCATGATCCACTCTAGGATCGGCCGCGTGGTGTTTGGCGTGAGGAACAGCAAACGGGGCGCCGCAGGCTCCCTGATGAACGTGC
TGAACTACCCCGGCATGAATCACCGCGTCGAAATTACCGAGGGAATCCTGGCAGATGAATGTGCCGCCCTGCTGTGCGACTTCTAC
CGGATGCCTAGACAGGTGTTCAATGCTCAGAAGAAGGCCCAGAGCTCCATCAACGGCTCCTCTGGCTCTGAGACACCTGGCACAA
GCGAGAGCGCAACACCTGAAAGCAGCGGCGGATCCGAGAGGCCACTGACCGACACAGAGAGGGCCACCCTGATGGATGAGCCTT
ACCGGAAGTCTAAGCTGACATATGCCCAGGCCAGAAAGCTGCTGGGCCTGGAGGACACCGCCTTCTTTAAGGGCCTGAGATACGG
CAAGGATAATGCCGAGGCCTCCACACTGATGGAGATGAAGGCCTATCACGCCATCTCTCGCGCCCTGGAGAAGGAGGGCCTGAAG
GACAAGAAGTCCCCCCTGAACCTGAGCTCCGAGCTGCAGGATGAGATCGGCACCGCCTTCTCTCTGTTTAAGACCGACGAGGATAT
CACAGGCCGCCTGAAGGACAGGGTGCAGCCTGAGATCCTGGAGGCCCTGCTGAAGCACATCTCTTTCGATAAGTTTGTGCAGATCA
GCCTGAAGGCCCTGAGAAGGATCGTGCCACTGATGGAGCAGGGCAAGCGGTACGACGAGGCCTGCGCCGAGATCTACGGCGATC
ACTATGGCAAGAAGAACACAGAGGAGAAGATCTATCTGCCCCCTATCCCTGCCGACGAGATCAGAAATCCTGTGGTGCTGAGGGC
CCTGTCCCAGGCAAGAAAAGTGATCAACGGAGTGGTGCGCCGGTACGGATCTCCAGCCCGGATCCACATCGAGACCGCCAGAGAA
GTGGGCAAGAGCTTCAAGGACCGGAAGGAGATCGAGAAGAGACAGGAGGAGAATCGCAAGGATCGGGAGAAGGCCGCCGCCA
AGTTTAGGGAGTACTTCCCTAACTTTGTGGGCGAGCCAAAGTCTAAGGACATCCTGAAGCTGCGCCTGTACGAGCAGCAGCACGG
CAAGTGTCTGTATAGCGGCAAGGAGATCAATCTGGTGCGGCTGAACGAGAAGGGCTATGTGGAGATCGATCACGCCCTGCCTTTC
TCCAGAACCTGGGACGATTCTTTTAACAATAAGGTGCTGGTGCTGGGCAGCGAGAACCAGAATAAGGGCAATCAGACACCATACG
AGTATTTCAATGGCAAGGACAACTCCAGGGAGTGGCAGGAGTTCAAGGCCCGCGTGGAGACCTCTAGATTTCCCAGGAGCAAGAA
GCAGCGGATCCTGCTGCAGAAGTTCGACGAGGATGGCTTTAAGGAGTGCAACCTGAATGACACCAGATACGTGAACCGGTTCCTG
TGCCAGTTTGTGGCCGATCACATCCTGCTGACCGGCAAGGGCAAGAGAAGGGTGTTCGCCTCTAATGGCCAGATCACAAACCTGCT
GAGGGGATTTTGGGGACTGAGGAAGGTGCGGGCAGAGAATGACAGACACCACGCACTGGATGCAGTGGTGGTGGCATGCAGCA
CCGTGGCAATGCAGCAGAAGATCACAAGATTCGTGAGGTATAAGGAGATGAACGCCTTTGACGGCAAGACCATCGATAAGGAGA
CAGGCAAGGTGCTGCACCAGAAGACCCACTTCCCCCAGCCTTGGGAGTTCTTTGCCCAGGAAGTGATGATCCGGGTGTTCGGCAA
GCCAGACGGCAAGCCTGAGTTTGAGGAGGCCGATACCCCAGAGAAGCTGAGGACACTGCTGGCAGAGAAGCTGTCTAGCAGGCC
AGAGGCAGTGCACGAGTACGTGACCCCACTGTTCGTGTCCAGGGCACCCAATCGGAAGATGTCTGGCGCCCACAAGGACACACTG
AGAAGCGCCAAGAGGTTTGTGAAGCACAACGAGAAGATCTCCGTGAAGAGAGTGTGGCTGACCGAGATCAAGCTGGCCGATCTG
GAGAACATGGTGAATTACAAGAACGGCAGGGAGATCGAGCTGTATGAGGCCCTGAAGGCAAGGCTGGAGGCCTACGGAGGAAA
TGCCAAGCAGGCCTTCGACCCAAAGGATAACCCCTTTTATAAGAAGGGAGGACAGCTGGTGAAGGCCGTGCGGGTGGAGAAGAC
CCAGGAGAGCGGCGTGCTGCTGAATAAGAAGAACGCCTACACAATCGCCGACAACGCCACCATGGTGCGGGTGGACGTGTACAC
CAAGGCCGGCAAGAACTACCTGGTTCCTGTGTACGTGTGGCAGGTGGCCCAGGGCATCTTACCCAACCGCGCCGTGACCAGCGGC
AAGTCCGAGGCTGACTGGGACCTGATCGATGAGAGCTTCGAGTTCAAGTTCTCTCTGTCCCGGGGAGATCTCGTGGAAATGATCTC
CAACAAGGGCAGAATCTTCGGCTACTACAACGGCCTGGACAGAGCCAACGGCTCTATTGGAATTAGAGAGCACGACCTAGAGAAG
AGCAAGGGCAAAGACGGCGTGCATAGAGTGGGAGTGAAAACAGCTACAGCATTTAACAAGTACCACGTGGATCCCCTGGGCAAA
GAGATCCACAGATGCAGCAGCGAACCCAGACCTACACTGAAAATCAAGTCTAAGAAGGAGGATAAAAGAACCGCCGACGGCAGC
GAATTCGAGCCCAAGAAGAAGAGGAAAGTC

Nme2^{Smu}-ABE8e-i7: BPSV40-NLS, Nme2Cas9 – delta PID, TadA8e, SmuCas9 PID, Linkers

AAACGGACAGCCGACGGAAGCGAGTTCGAGTCACCAAAGAAGAAGCGGAAAGTCGAAGATATGGCCGCCTTCAAGCCTAACCCA
ATCAATTACATCCTGGGACTGGCCATCGGAATCGCATCCGTGGGATGGGCTATGGTGGAGATCGACGAGGAGGAGAATCCTATCC
GGCTGATCGATCTGGGCGTGAGAGTGTTTGAGAGGGCCGAGGTGCCAAAGACCGGCGATTCTCTGGCTATGGCCCGGAGACTGG
CACGGAGCGTGAGGCGCCTGACACGGAGAAGGGCACACAGGCTGCTGAGGGCACGCCGGCTGCTGAAGAGAGAGGGCGTGCTG
CAGGCAGCAGACTTCGATGAGAATGGCCTGATCAAGAGCCTGCCAAACACCCCCTGGCAGCTGAGAGCAGCCGCCCTGGACAGGA
AGCTGACACCACTGGAGTGGTCTGCCGTGCTGCTGCACCTGATCAAGCACCGCGGCTACCTGAGCCAGCGGAAGAACGAGGGAG
AGACAGCAGACAAGGAGCTGGGCGCCCTGCTGAAGGGAGTGGCCAACAATGCCCACGCCCTGCAGACCGGCGATTTCAGGACAC
CTGCCGAGCTGGCCCTGAATAAGTTTGAGAAGGAGTCCGGCCACATCAGAAACCAGAGGGGCGACTATAGCCACACCTTCTCCCG
CAAGGATCTGCAGGCCGAGCTGATCCTGCTGTTCGAGAAGCAGAAGGAGTTTGGCAATCCACACGTGAGCGGAGGCCTGAAGGA
GGGAATCGAGACCCTGCTGATGACACAGAGGCCTGCCCTGTCCGGCGACGCAGTGCAGAAGATGCTGGGACACTGCACCTTCGAG
CCTGCAGAGCCAAAGGCCGCCAAGAACACCTACACAGCCGAGCGGTTTATCTGGCTGACAAAGCTGAACAATCTGAGAATCCTGG
AGCAGGGATCCGAGAGGCCACTGACCGACACAGAGAGGGCCACCCTGATGGATGAGCCTTACCGGAAGTCTAAGCTGACATATG
CCCAGGCCAGAAAGCTGCTGGGCCTGGAGGACACCGCCTTCTTTAAGGGCCTGAGATACGGCAAGGATAATGCCGAGGCCTCCAC
ACTGATGGAGATGAAGGCCTATCACGCCATCTCTCGCGCCCTGGAGAAGGAGGGCCTGAAGGACAAGAAGTCCCCCCTGAACCTG
AGCTCCGAGCTGCAGGATGAGATCGGCACCGCCTTCTCTCTGTTTAAGACCGACGAGGATATCACAGGCCGCCTGAAGGACAGGG
TGCAGCCTGAGATCCTGGAGGCCCTGCTGAAGCACATCTCTTTCGATAAGTTTGTGCAGATCAGCCTGAAGGCCCTGAGAAGGATC
GTGCCACTGATGGAGCAGGGCAAGCGGTACGACGAGGCCTGCGCCGAGATCTACGGCGATCACTATGGCAAGAAGAACACAGAG
GAGAAGATCTATCTGCCCCCTATCCCTGCCGACGAGATCAGAAATCCTGTGGTGCTGAGGGCCCTGTCCCAGGCAAGAAAAGTGAT
CAACGGAGTGGTGCGCCGGTACGGATCTCCAGCCCGGATCCACATCGAGACCGCCAGAGAAGTGGGCAAGAGCTTCAAGGACCG
GAAGGAGATCGAGAAGAGACAGGAGGAGAATCGCAAGGATCGGGAGAAGGCCGCCGCCAAGTTTAGGGAGTACTTCCCTAACTT
TGTGGGCGAGCCAAAGTCTAAGGACATCCTGAAGCTGCGCCTGTACGAGCAGCAGCACGGCAAGTGTCTGTATAGCGGCAAGGA
GATCAATCTGGTGCGGCTGAACGAGAAGGGCTATGTGGAGATCGATCACGCCCTGCCTTTCTCCAGAACCTGGGACGATTCTTTTA
ACAATAAGGTGCTGGTGCTGGGCAGCGAGAACCAGAATAAGGGCAATCAGACACCATACGAGTATTTCAATGGCAAGGACAACTC
CAGGGAGTGGCAGGAGTTCAAGGCCCGCGTGGAGACCTCTAGATTTCCCAGGAGCAAGAAGCAGCGGATCCTGCTGCAGAAGTT
CGACGAGGATGGCTTTAAGGAGTGCAACCTGAATGACACCAGATACGTGAACCGGTTCCTGTGCCAGTTTGTGGCCGATCACATCC
TGCTGACCGGCAAGGGCAAGAGAAGGGTGTTCGCCTCTAATGGCCAGATCACAAACCTGCTGAGGGGATTTTGGGGACTGAGGA
AGGTGCGGGCAGAGAATGACAGACACCACGCACTGGATGCAGTGGTGGTGGCATGCAGCACCGTGGCAATGCAGCAGAAGATCA
CAAGATTCGTGAGGTATAAGGAGATGAACGCCTTTGACGGCAAGACCATCGATAAGGAGGGCGGATCAGGAGGCTCTGGCGGTT
CAGGTGGATCAGGCGGTAGCGGAGGTTCAGGTGGTTCTGAGGTGGAGTTTTCCCACGAGTACTGGATGAGACATGCCCTGACCCT
GGCCAAGAGGGCACGCGATGAGAGGGAGGTGCCTGTGGGAGCCGTGCTGGTGCTGAACAATAGAGTGATCGGCGAGGGCTGGA
ACAGAGCCATCGGCCTGCACGACCCAACAGCCCATGCCGAAATTATGGCCCTGAGACAGGGCGGCCTGGTCATGCAGAACTACAG
ACTGATTGACGCCACCCTGTACGTGACATTCGAGCCTTGCGTGATGTGCGCCGGCGCCATGATCCACTCTAGGATCGGCCGCGTGG
TGTTTGGCGTGAGGAACAGCAAACGGGGCGCCGCAGGCTCCCTGATGAACGTGCTGAACTACCCCGGCATGAATCACCGCGTCGA
AATTACCGAGGGAATCCTGGCAGATGAATGTGCCGCCCTGCTGTGCGACTTCTACCGGATGCCTAGACAGGTGTTCAATGCTCAGA
AGAAGGCCCAGAGCTCCATCAACGGCTCCTCTGGCTCTGAGACACCTGGCACAAGCGAGAGCGCAACACCTGAAAGCAGCGGCAC
AGGCAAGGTGCTGCACCAGAAGACCCACTTCCCCCAGCCTTGGGAGTTCTTTGCCCAGGAAGTGATGATCCGGGTGTTCGGCAAG
CCAGACGGCAAGCCTGAGTTTGAGGAGGCCGATACCCCAGAGAAGCTGAGGACACTGCTGGCAGAGAAGCTGTCTAGCAGGCCA
GAGGCAGTGCACGAGTACGTGACCCCACTGTTCGTGTCCAGGGCACCCAATCGGAAGATGTCTGGCGCCCACAAGGACACACTGA
GAAGCGCCAAGAGGTTTGTGAAGCACAACGAGAAGATCTCCGTGAAGAGAGTGTGGCTGACCGAGATCAAGCTGGCCGATCTGG
AGAACATGGTGAATTACAAGAACGGCAGGGAGATCGAGCTGTATGAGGCCCTGAAGGCAAGGCTGGAGGCCTACGGAGGAAAT
GCCAAGCAGGCCTTCGACCCAAAGGATAACCCCTTTTATAAGAAGGGAGGACAGCTGGTGAAGGCCGTGCGGGTGGAGAAGACC
CAGGAGAGCGGCGTGCTGCTGAATAAGAAGAACGCCTACACAATCGCCGACAACGCCACCATGGTGCGGGTGGACGTGTACACC
AAGGCCGGCAAGAACTACCTGGTTCCTGTGTACGTGTGGCAGGTGGCCCAGGGCATCTTACCCAACCGCGCCGTGACCAGCGGCA
AGTCCGAGGCTGACTGGGACCTGATCGATGAGAGCTTCGAGTTCAAGTTCTCTCTGTCCCGGGGAGATCTCGTGGAAATGATCTCC
AACAAGGGCAGAATCTTCGGCTACTACAACGGCCTGGACAGAGCCAACGGCTCTATTGGAATTAGAGAGCACGACCTAGAGAAGA
GCAAGGGCAAAGACGGCGTGCATAGAGTGGGAGTGAAAACAGCTACAGCATTTAACAAGTACCACGTGGATCCCCTGGGCAAAG
AGATCCACAGATGCAGCAGCGAACCCAGACCTACACTGAAAATCAAGTCTAAGAAGGAGGATAAAAGAACCGCCGACGGCAGCG
AATTCGAGCCCAAGAAGAAGAGGAAAGTC

Nme2^{Smu}-ABE8e-i8: BPSV40-NLS, Nme2Cas9 – delta PID, TadA8e, SmuCas9 PID, Linkers

AAACGGACAGCCGACGGAAGCGAGTTCGAGTCACCAAAGAAGAAGCGGAAAGTCGAAGATATGGCCGCCTTCAAGCCTAACCCA
ATCAATTACATCCTGGGACTGGCCATCGGAATCGCATCCGTGGGATGGGCTATGGTGGAGATCGACGAGGAGGAGAATCCTATCC
GGCTGATCGATCTGGGCGTGAGAGTGTTTGAGAGGGCCGAGGTGCCAAAGACCGGCGATTCTCTGGCTATGGCCCGGAGACTGG
CACGGAGCGTGAGGCGCCTGACACGGAGAAGGGCACACAGGCTGCTGAGGGCACGCCGGCTGCTGAAGAGAGAGGGCGTGCTG
CAGGCAGCAGACTTCGATGAGAATGGCCTGATCAAGAGCCTGCCAAACACCCCCTGGCAGCTGAGAGCAGCCGCCCTGGACAGGA
AGCTGACACCACTGGAGTGGTCTGCCGTGCTGCTGCACCTGATCAAGCACCGCGGCTACCTGAGCCAGCGGAAGAACGAGGGAG
AGACAGCAGACAAGGAGCTGGGCGCCCTGCTGAAGGGAGTGGCCAACAATGCCCACGCCCTGCAGACCGGCGATTTCAGGACAC
CTGCCGAGCTGGCCCTGAATAAGTTTGAGAAGGAGTCCGGCCACATCAGAAACCAGAGGGGCGACTATAGCCACACCTTCTCCCG
CAAGGATCTGCAGGCCGAGCTGATCCTGCTGTTCGAGAAGCAGAAGGAGTTTGGCAATCCACACGTGAGCGGAGGCCTGAAGGA
GGGAATCGAGACCCTGCTGATGACACAGAGGCCTGCCCTGTCCGGCGACGCAGTGCAGAAGATGCTGGGACACTGCACCTTCGAG
CCTGCAGAGCCAAAGGCCGCCAAGAACACCTACACAGCCGAGCGGTTTATCTGGCTGACAAAGCTGAACAATCTGAGAATCCTGG
AGCAGGGATCCGAGAGGCCACTGACCGACACAGAGAGGGCCACCCTGATGGATGAGCCTTACCGGAAGTCTAAGCTGACATATG
CCCAGGCCAGAAAGCTGCTGGGCCTGGAGGACACCGCCTTCTTTAAGGGCCTGAGATACGGCAAGGATAATGCCGAGGCCTCCAC
ACTGATGGAGATGAAGGCCTATCACGCCATCTCTCGCGCCCTGGAGAAGGAGGGCCTGAAGGACAAGAAGTCCCCCCTGAACCTG
AGCTCCGAGCTGCAGGATGAGATCGGCACCGCCTTCTCTCTGTTTAAGACCGACGAGGATATCACAGGCCGCCTGAAGGACAGGG
TGCAGCCTGAGATCCTGGAGGCCCTGCTGAAGCACATCTCTTTCGATAAGTTTGTGCAGATCAGCCTGAAGGCCCTGAGAAGGATC
GTGCCACTGATGGAGCAGGGCAAGCGGTACGACGAGGCCTGCGCCGAGATCTACGGCGATCACTATGGCAAGAAGAACACAGAG
GAGAAGATCTATCTGCCCCCTATCCCTGCCGACGAGATCAGAAATCCTGTGGTGCTGAGGGCCCTGTCCCAGGCAAGAAAAGTGAT
CAACGGAGTGGTGCGCCGGTACGGATCTCCAGCCCGGATCCACATCGAGACCGCCAGAGAAGTGGGCAAGAGCTTCAAGGACCG
GAAGGAGATCGAGAAGAGACAGGAGGAGAATCGCAAGGATCGGGAGAAGGCCGCCGCCAAGTTTAGGGAGTACTTCCCTAACTT
TGTGGGCGAGCCAAAGTCTAAGGACATCCTGAAGCTGCGCCTGTACGAGCAGCAGCACGGCAAGTGTCTGTATAGCGGCAAGGA
GATCAATCTGGTGCGGCTGAACGAGAAGGGCTATGTGGAGATCGATCACGCCCTGCCTTTCTCCAGAACCTGGGACGATTCTTTTA
ACAATAAGGTGCTGGTGCTGGGCAGCGAGAACCAGAATAAGGGCAATCAGACACCATACGAGTATTTCAATGGCAAGGACAACTC
CAGGGAGTGGCAGGAGTTCAAGGCCCGCGTGGAGACCTCTAGATTTCCCAGGAGCAAGAAGCAGCGGATCCTGCTGCAGAAGTT
CGACGAGGATGGCTTTAAGGAGTGCAACCTGAATGACACCAGATACGTGAACCGGTTCCTGTGCCAGTTTGTGGCCGATCACATCC
TGCTGACCGGCAAGGGCAAGAGAAGGGTGTTCGCCTCTAATGGCCAGATCACAAACCTGCTGAGGGGATTTTGGGGACTGAGGA
AGGTGCGGGCAGAGAATGACAGACACCACGCACTGGATGCAGTGGTGGTGGCATGCAGCACCGTGGCAATGCAGCAGAAGATCA
CAAGATTCGTGAGGTATAAGGAGATGAACGCCTTTGACGGCAAGACCATCGATAAGGAGACAGGCAAGGTGCTGCACCAGAAGA
CCCACTTCCCCCAGCCTTGGGAGTTCTTTGCCCAGGAAGTGATGATCCGGGTGTTCGGCAAGCCAGACGGCAAGCCTGGCGGATCA
GGAGGCTCTGGCGGTTCAGGTGGATCAGGCGGTAGCGGAGGTTCAGGTGGTTCTGAGGTGGAGTTTTCCCACGAGTACTGGATG
AGACATGCCCTGACCCTGGCCAAGAGGGCACGCGATGAGAGGGAGGTGCCTGTGGGAGCCGTGCTGGTGCTGAACAATAGAGTG
ATCGGCGAGGGCTGGAACAGAGCCATCGGCCTGCACGACCCAACAGCCCATGCCGAAATTATGGCCCTGAGACAGGGCGGCCTG
GTCATGCAGAACTACAGACTGATTGACGCCACCCTGTACGTGACATTCGAGCCTTGCGTGATGTGCGCCGGCGCCATGATCCACTC
TAGGATCGGCCGCGTGGTGTTTGGCGTGAGGAACAGCAAACGGGGCGCCGCAGGCTCCCTGATGAACGTGCTGAACTACCCCGG
CATGAATCACCGCGTCGAAATTACCGAGGGAATCCTGGCAGATGAATGTGCCGCCCTGCTGTGCGACTTCTACCGGATGCCTAGAC
AGGTGTTCAATGCTCAGAAGAAGGCCCAGAGCTCCATCAACGGCTCCTCTGGCTCTGAGACACCTGGCACAAGCGAGAGCGCAAC
ACCTGAAAGCAGCGGCGAGTTTGAGGAGGCCGATACCCCAGAGAAGCTGAGGACACTGCTGGCAGAGAAGCTGTCTAGCAGGCC
AGAGGCAGTGCACGAGTACGTGACCCCACTGTTCGTGTCCAGGGCACCCAATCGGAAGATGTCTGGCGCCCACAAGGACACACTG
AGAAGCGCCAAGAGGTTTGTGAAGCACAACGAGAAGATCTCCGTGAAGAGAGTGTGGCTGACCGAGATCAAGCTGGCCGATCTG
GAGAACATGGTGAATTACAAGAACGGCAGGGAGATCGAGCTGTATGAGGCCCTGAAGGCAAGGCTGGAGGCCTACGGAGGAAA
TGCCAAGCAGGCCTTCGACCCAAAGGATAACCCCTTTTATAAGAAGGGAGGACAGCTGGTGAAGGCCGTGCGGGTGGAGAAGAC
CCAGGAGAGCGGCGTGCTGCTGAATAAGAAGAACGCCTACACAATCGCCGACAACGCCACCATGGTGCGGGTGGACGTGTACAC
CAAGGCCGGCAAGAACTACCTGGTTCCTGTGTACGTGTGGCAGGTGGCCCAGGGCATCTTACCCAACCGCGCCGTGACCAGCGGC
AAGTCCGAGGCTGACTGGGACCTGATCGATGAGAGCTTCGAGTTCAAGTTCTCTCTGTCCCGGGGAGATCTCGTGGAAATGATCTC
CAACAAGGGCAGAATCTTCGGCTACTACAACGGCCTGGACAGAGCCAACGGCTCTATTGGAATTAGAGAGCACGACCTAGAGAAG
AGCAAGGGCAAAGACGGCGTGCATAGAGTGGGAGTGAAAACAGCTACAGCATTTAACAAGTACCACGTGGATCCCCTGGGCAAA
GAGATCCACAGATGCAGCAGCGAACCCAGACCTACACTGAAAATCAAGTCTAAGAAGGAGGATAAAAGAACCGCCGACGGCAGC
GAATTCGAGCCCAAGAAGAAGAGGAAAGTC

eNme2-C: BPSV40-NLS, TadA8e, eNme2-C, Linkers

AAACGGACAGCCGACGGAAGCGAGTTCGAGTCACCAAAGAAGAAGCGGAAAGTCTCTGAGGTGGAGTTTTCCCACGAGTACTGG
ATGAGACATGCCCTGACCCTGGCCAAGAGGGCACGGGATGAGAGGGAGGTGCCTGTGGGAGCCGTGCTGGTGCTGAACAATAGA
GTGATCGGCGAGGGCTGGAACAGAGCCATCGGCCTGCACGACCCAACAGCCCATGCCGAAATTATGGCCCTGAGACAGGGCGGC
CTGGTCATGCAGAACTACAGACTGATTGACGCCACCCTGTACGTGACATTCGAGCCTTGCGTGATGTGCGCCGGCGCCATGATCCA
CTCTAGGATCGGCCGCGTGGTGTTTGGCGTGAGGAACTCAAAAAGAGGCGCCGCAGGCTCCCTGATGAACGTGCTGAACTACCCC
GGCATGAATCACCGCGTCGAAATTACCGAGGGAATCCTGGCAGATGAATGTGCCGCCCTGCTGTGCGATTTCTATCGGATGCCTAG
ACAGGTGTTCAATGCTCAGAAGAAGGCCCAGAGCTCCATCAACTCTGGAGGATCTAGCGGAGGATCCTCTGGCAGCGAGACACCA
GGAACAAGCGAGTCAGCAACACCAGAGAGCAGTGGCGGCAGCAGCGGCGGCAGCGCAGCATTCAAGTCAAACCCAATCAATTAC
ATCCTGGGACTGGCAATCGGAATCGCATCCGTGGGATGGCTATGGTGGAGATCGACGAGGAGGGGAATCCTATCCGGCTGATC
GATCTGGGCGTGAGAGTGTTTGAGAGGGCCGAGGTGCCAAAGACCGGCGATTCTCTGGCTATGGCCCGGAGACTGGCACGGAGC
GTGAGGCGCCTGACACGGAGAAGGGCACACAGGCTGCTGAGGGCACGCCGGCTGCTGAAGAGAGAGGGCGTGCTGCAGGCAGC
AGACTTCGATGAGAATGGCCTGATCACGAGCTTGCCAAACACCCCCTGGCAGCTGAGAGCAGCCGCCCTGGACAGGAAGCTGACA
CCACTGGAGTGGTCTGCCGTGCTGCTGCACCTGATCAAGCACCGCGGCTACCTGAGCCAGCGGAAGAACGAGGGAGAGACAGCA
GCCAAGGAGCTGGGCGCCCTGCTGAAGGGAGTGGCCAACAATGCCCACGCCCTGCAGACCGGCGATTTCAGGACACCTGCCGAG
CTGGCCCTGAATAAGTTTGAGAAGGAGTCCGGCCACATCAGAAACCAGAGGGGCGACTATAGCCACACCTTCTCCCGCAAGGATCT
GCAGGCCGAGCTGATCCTGCTGTTCGAGAAGCAGAAGGAGTTTGGCAATCCACACGTGAGCGGAGGCCTGAAGGAGGGAATCGA
GACCCTGCTGATGACACAGAGGCCTGCCCTGTCCGGCGACGCAGTGCAGAAGATGCTGGGGCACTGCACCCTCGAGCCTACAGAG
CCAAAGGCCGCCAAGAACACCTACACAGCCGAGCGGTTTATCTGGCTGACAAAGCTGAACAATCTGAGAATCCTGGAGCAGGGAT
CCGAGAGGCCACTGACCGACACAGAGAGGTCCACCCTGATGGATGAGCCTTACCGGAAGTCTAAACTGACATATGCCCAGGCCAG
AAAGCTGCTGGGCCTGGAGGACACCGCCTTCTTTAAGGGCCTGAGATACGGCAAGGATAATGCCGAGGCCTCCACACTGATGGAG
ATGAAGGCCTATCACGCCATCTCTCGCGCCCTGGAGAAGGAGGGCCTGAAGGACAAGAAGTCCCCCCTGAACCTGAGCTCCGAGC
TGCAGGATGAGATCGGCACCGCCTTCTCTCTGTTTAAGACCGACGAGGATATCACAGGCCGCCTGAAGGACAGGGTGCAGCCTGA
GATCCTGGAGGCCCTGCTGAAGCACATCTCTTTCGATAAGTTTGTGCAGATCAGCCTGAAGGCCCTGAGAAGGATCGTGCCACTGA
TGGAGCAGGGCAAGCGGTACGACGAGGCCTGCGCCGAGATCTACGGCGTTCACTATGGCAAGAAGAACACAGAGGAGAAGATCT
ATCTGCCCCCTATCCCTGCCGACGAGATCAGAAATCCTGTGGTGCTGAGGGCCCTGTCCCAGGCAAGAAAAGTGATCAACGGAGT
GGTGCGCCGGTACGGATCTCCAGCCCGGATCCACATCGAGACCGCCAGAGAAGTGGGCAAGAGCTTCAAGGACCGGAAGGAGAT
CGCGAAGAGACAGGAGGAGAATCGCAAGGATCGGGAGAAGGCCGCCGCCAAGTTTAGGGAGTACTTCCCTAACTTTGTGGGCGA
GCCAAAGTCTAAGGACATCCTGAAGCTGCGCCTGTACGAGCAGCAGCACGGCAAGTGTCTGTATAGCGGCAAAGAGATCAATCTG
GTGCGGCTGAACGAGAAGGGCTATGTGGAGATCGATCACGCCCTGCCTTTCTCCAGAACCTGGGACGATTCTTTTAACAATAAGGT
GCTGGTGCTGGGCAGCGAGAACCAGAATAAGGGCAATCAGACACCATACGAGTATTTCAATGGCAAGGACAACTCCAGGGAGTG
GCAGGAGTTCAAGGCCCGCGTGGAGACCTCTAGATTTCCCAGTAGCAAGAAGCAGCGGATCCTGCTGCAGAAGTTCGACGAGGAT
GGCTTTAAGGAGTGCAACCTGAATGACACCAGATACGTGAACCGGTTCCTGTGCCAGTTTGTGGCCGATCACATCCTGCTGACCGG
CAAGGGCAAGAGAAGGGTGGTCGCCTCTAATGGCCAGATCACAAACCTGCTGAGGGGGTTTTGGAGACTGAGGAAGGTGCGGGC
AGAGAATGACAGACACCACGCACTGGATGCAGTGGTGGTGGCATGCAGCACCGTGGCAATGCAGCAGAAGATCACAAGATTCGT
GAGGTATAAGGAGATGAACGCCTTTGACGGCAAGACCGTCGATAAGGAGACAGGCAAGGTGCTGTACCAGAAGACCCACTTCCCC
CAGCCTTGGGAGTTCTTTGCCCAGGAAGTTATGATCCGGGTGTTCGGCAAGCCAGACGGCAAGCCTGAGTTTGAGGAGGCCGATA
CCCCAGAGAAGCTGAGGACACTGCTGGCAGAGAAGCTGTCTAGCAGGCCAGAGGCAGTGCACGAGTACGTGACCCCGCTGTTCGT
GTCCAGGGCACCCAATCGGAAGATGTCTGGCGCCCACAAGGACACACTGAGAAGCGCCAAGAGGTTTGTGAAGCACAACGAGAA
GATCTCCGTGAAGAGAGTGTGGCTGACCGAGATCAAGCTGGCCGATCTGGAGAACATGGTGAATTACAAGAACGGCAGGGAGAT
CGAGCTGTATGAGGCCCTGAAGGCAAGGCTGGAGGCCTACGGAGGAAATGCCAAGCAGGCCTTCGACCCAAAGGATAACCCCTTT
TATAAGAAGGGAGGACAGCTGGTGAAGGCCGTGCGGGTGGAGAAGACCCAGAAGAGCGGCGTGCTGCTGAATAAGAAGAACGC
CTACACAATCGCCGACAATGGTGATATGGTGAGAGTGGACGTGTTCTGTAAGGTGGATAAGAAGGGCAAGAATCAGTACTTTATC
GTGCCTATCTATGCCTGGCAGGTGGCCGAGAACATCCTGCCAGACATCGATTGCAAGGGCTACAGAATCGACGATAGCTATACATT
CTGTTTTTCCCTGCACAAGTATGACCTGATCGCCTTCCAGAAGGATGAGAAGTCCAAGGTGGAGTTTGCCTACTATATCAATTGCGA
CTCCTCTAGCGGCGGGTTCTACCTGGCCTGGCACGATAAGGGCAGCAGGGAGCAGCGGTTTCGCATCTCCACCCAGAATCTGGCG
CTGATCCAGAAGTATCAGGTGAACGAGCTGGGCAAGGAGATCAGGCCATGTCGGCTGAAGAAGCGCCCACCCGTGCGGTCTGGC
GGCTCAAAAAGAACCGCCGACGGCAGCGAATTCGAGCCCAAGAAGAAGAGGAAAGTC

Nme2-evoFERNY-nt: BPSV40-NLS, Nme2Cas9, EvoFERNY, UGI, Linkers

AAACGGACAGCCGACGGAAGCGAGTTCGAGTCACCAAAGAAGAAGCGGAAAGTCAGTTTTGAGAGGAACTACGACCCCCGGGAG
CTGAGAAAGGAGACATACCTGCTGTATGAGATCAAGTGGGGCAAGTCCGGCAAGCTGTGGAGGCACTGGTGCCAGAACAATCGC
ACACAGCACGCCGAGGTGTACTTCCTGGAGAACATCTTTAATGCCCGGAGATTCAATCCATCTACCCACTGTAGCATCACATGGTAT
CTGAGCTGGTCCCCCTGCGCCGAGTGTTCTCAGAAGATCGTGGATTTCCTGAAGGAGCACCCTAACGTGAATCTGGAGATCTATGT
GGCCCGGCTGTACTATCCAGAGAACGAGAGGAATAGGCAGGGCCTGCGGGATCTGGTGAATTCCGGCGTGACCATCAGAATCAT
GGACCTGCCAGATTACAACTATTGCTGGAAGACCTTCGTGAGCGATCAGGGAGGCGACGAGGATTACTGGCCAGGACACTTCGCC
CCTTGGATCAAGCAGTATAGCCTGAAGCTGTCTGGCGGATCTAGCGGAGGATCCTCTGGCAGCGAGACACCAGGAACAAGCGAGT
CAGCAACACCAGAGAGCAGTGGCGGCAGCAGCGGCGGCAGCGCCGCCTTCAAGCCTAACCCAATCAATTACATCCTGGGACTGGC
CATCGGAATCGCATCCGTGGGATGGGCTATGGTGGAGATCGACGAGGAGGAGAATCCTATCCGGCTGATCGATCTGGGCGTGAG
AGTGTTTGAGAGGGCCGAGGTGCCAAAGACCGGCGATTCTCTGGCTATGGCCCGGAGACTGGCACGGAGCGTGAGGCGCCTGAC
ACGGAGAAGGGCACACAGGCTGCTGAGGGCACGCCGGCTGCTGAAGAGAGAGGGCGTGCTGCAGGCAGCAGACTTCGATGAGA
ATGGCCTGATCAAGAGCCTGCCAAACACCCCCTGGCAGCTGAGAGCAGCCGCCCTGGACAGGAAGCTGACACCACTGGAGTGGTC
TGCCGTGCTGCTGCACCTGATCAAGCACCGCGGCTACCTGAGCCAGCGGAAGAACGAGGGAGAGACAGCAGACAAGGAGCTGGG
CGCCCTGCTGAAGGGAGTGGCCAACAATGCCCACGCCCTGCAGACCGGCGATTTCAGGACACCTGCCGAGCTGGCCCTGAATAAG
TTTGAGAAGGAGTCCGGCCACATCAGAAACCAGAGGGGCGACTATAGCCACACCTTCTCCCGCAAGGATCTGCAGGCCGAGCTGA
TCCTGCTGTTCGAGAAGCAGAAGGAGTTTGGCAATCCACACGTGAGCGGAGGCCTGAAGGAGGGAATCGAGACCCTGCTGATGA
CACAGAGGCCTGCCCTGTCCGGCGACGCAGTGCAGAAGATGCTGGGACACTGCACCTTCGAGCCTGCAGAGCCAAAGGCCGCCAA
GAACACCTACACAGCCGAGCGGTTTATCTGGCTGACAAAGCTGAACAATCTGAGAATCCTGGAGCAGGGATCCGAGAGGCCACTG
ACCGACACAGAGAGGGCCACCCTGATGGATGAGCCTTACCGGAAGTCTAAGCTGACATATGCCCAGGCCAGAAAGCTGCTGGGCC
TGGAGGACACCGCCTTCTTTAAGGGCCTGAGATACGGCAAGGATAATGCCGAGGCCTCCACACTGATGGAGATGAAGGCCTATCA
CGCCATCTCTCGCGCCCTGGAGAAGGAGGGCCTGAAGGACAAGAAGTCCCCCCTGAACCTGAGCTCCGAGCTGCAGGATGAGATC
GGCACCGCCTTCTCTCTGTTTAAGACCGACGAGGATATCACAGGCCGCCTGAAGGACAGGGTGCAGCCTGAGATCCTGGAGGCCC
TGCTGAAGCACATCTCTTTCGATAAGTTTGTGCAGATCAGCCTGAAGGCCCTGAGAAGGATCGTGCCACTGATGGAGCAGGGCAA
GCGGTACGACGAGGCCTGCGCCGAGATCTACGGCGATCACTATGGCAAGAAGAACACAGAGGAGAAGATCTATCTGCCCCCTATC
CCTGCCGACGAGATCAGAAATCCTGTGGTGCTGAGGGCCCTGTCCCAGGCAAGAAAAGTGATCAACGGAGTGGTGCGCCGGTAC
GGATCTCCAGCCCGGATCCACATCGAGACCGCCAGAGAAGTGGGCAAGAGCTTCAAGGACCGGAAGGAGATCGAGAAGAGACAG
GAGGAGAATCGCAAGGATCGGGAGAAGGCCGCCGCCAAGTTTAGGGAGTACTTCCCTAACTTTGTGGGCGAGCCAAAGTCTAAG
GACATCCTGAAGCTGCGCCTGTACGAGCAGCAGCACGGCAAGTGTCTGTATAGCGGCAAGGAGATCAATCTGGTGCGGCTGAACG
AGAAGGGCTATGTGGAGATCGATCACGCCCTGCCTTTCTCCAGAACCTGGGACGATTCTTTTAACAATAAGGTGCTGGTGCTGGGC
AGCGAGAACCAGAATAAGGGCAATCAGACACCATACGAGTATTTCAATGGCAAGGACAACTCCAGGGAGTGGCAGGAGTTCAAG
GCCCGCGTGGAGACCTCTAGATTTCCCAGGAGCAAGAAGCAGCGGATCCTGCTGCAGAAGTTCGACGAGGATGGCTTTAAGGAGT
GCAACCTGAATGACACCAGATACGTGAACCGGTTCCTGTGCCAGTTTGTGGCCGATCACATCCTGCTGACCGGCAAGGGCAAGAG
AAGGGTGTTCGCCTCTAATGGCCAGATCACAAACCTGCTGAGGGGATTTTGGGGACTGAGGAAGGTGCGGGCAGAGAATGACAG
ACACCACGCACTGGATGCAGTGGTGGTGGCATGCAGCACCGTGGCAATGCAGCAGAAGATCACAAGATTCGTGAGGTATAAGGA
GATGAACGCCTTTGACGGCAAGACCATCGATAAGGAGACAGGCAAGGTGCTGCACCAGAAGACCCACTTCCCCCAGCCTTGGGAG
TTCTTTGCCCAGGAAGTGATGATCCGGGTGTTCGGCAAGCCAGACGGCAAGCCTGAGTTTGAGGAGGCCGATACCCCAGAGAAGC
TGAGGACACTGCTGGCAGAGAAGCTGTCTAGCAGGCCAGAGGCAGTGCACGAGTACGTGACCCCACTGTTCGTGTCCAGGGCACC
CAATCGGAAGATGTCTGGCGCCCACAAGGACACACTGAGAAGCGCCAAGAGGTTTGTGAAGCACAACGAGAAGATCTCCGTGAA
GAGAGTGTGGCTGACCGAGATCAAGCTGGCCGATCTGGAGAACATGGTGAATTACAAGAACGGCAGGGAGATCGAGCTGTATGA
GGCCCTGAAGGCAAGGCTGGAGGCCTACGGAGGAAATGCCAAGCAGGCCTTCGACCCAAAGGATAACCCCTTTTATAAGAAGGG
AGGACAGCTGGTGAAGGCCGTGCGGGTGGAGAAGACCCAGGAGAGCGGCGTGCTGCTGAATAAGAAGAACGCCTACACAATCG
CCGACAATGGCGATATGGTGAGAGTGGACGTGTTCTGTAAGGTGGATAAGAAGGGCAAGAATCAGTACTTTATCGTGCCTATCTA
TGCCTGGCAGGTGGCCGAGAACATCCTGCCAGACATCGATTGCAAGGGCTACAGAATCGACGATAGCTATACATTCTGTTTTTCCCT
GCACAAGTATGACCTGATCGCCTTCCAGAAGGATGAGAAGTCCAAGGTGGAGTTTGCCTACTATATCAATTGCGACTCCTCTAACG
GCAGGTTCTACCTGGCCTGGCACGATAAGGGCAGCAAGGAGCAGCAGTTTCGCATCTCCACCCAGAATCTGGTGCTGATCCAGAA
GTATCAGGTGAACGAGCTGGGCAAGGAGATCAGGCCATGTCGGCTGAAGAAGCGCCCACCCGTGCGGAGCGGAGGATCCGGAG
GATCTGGAGGCAGCACCAACCTGTCTGACATCATCGAGAAGGAGACAGGCAAGCAGCTGGTCATCCAGGAGAGCATCCTGATGCT
GCCCGAAGAAGTCGAAGAAGTGATCGGAAACAAGCCTGAGAGCGATATCCTGGTCCATACCGCCTACGACGAGAGTACCGACGA
AAATGTGATGCTGCTGACATCCGACGCCCCAGAGTATAAGCCCTGGGCTCTGGTCATCCAGGATTCCAACGGAGAGAACAAAATCA
AAATGCTGTCTGGCGGCTCAAAAAGAACCGCCGACGGCAGCGAATTCGAGCCCAAGAAGAAGAGGAAAGTC

Nme2-evoFERNY-i1: BPSV40-NLS, Nme2Cas9, EvoFERNY, UGI, Linkers

AAACGGACAGCCGACGGAAGCGAGTTCGAGTCACCAAAGAAGAAGCGGAAAGTCGAAGATATGGCCGCCTTCAAGCCTAACCCA
ATCAATTACATCCTGGGACTGGCCATCGGAATCGCATCCGTGGGATGGGCTATGGTGGAGATCGACGAGGAGGAGAATCCTATCC
GGCTGATCGATCTGGGCGTGAGAGTGTTTGAGAGGGCCGAGGTGCCAAAGACCGGCGATTCTCTGGCTATGGCCCGGAGACTGG
CACGGAGCGTGAGGCGCCTGACACGGAGAAGGGCACACAGGCTGCTGAGGGCACGCCGGCTGCTGAAGAGAGAGGGCGTGCTG
CAGGCAGCAGACTTCGATGAGAATGGCCTGATCAAGAGCCTGCCAAACACCCCCTGGCAGCTGAGAGCAGCCGCCCTGGACAGGA
AGCTGACACCACTGGAGTGGTCTGCCGTGCTGCTGCACCTGATCAAGCACCGCGGCTACCTGAGCCAGCGGAAGAACGAGGGAG
AGACAGCAGACAAGGAGCTGGGCGCCCTGCTGAAGGGAGTGGCCAACAATGCCCACGCCCTGCAGACCGGCGATTTCAGGACAC
CTGCCGAGCTGGCCCTGAATAAGTTTGAGAAGGAGTCCGGCCACATCAGAAACCAGAGGGGCGACTATAGCCACACCTTCTCCCG
CAAGGATCTGCAGGCCGAGCTGATCCTGCTGTTCGAGAAGCAGAAGGAGTTTGGCAATCCACACGTGAGCGGAGGCCTGAAGGA
GGGAATCGAGACCCTGCTGATGACACAGAGGCCTGCCCTGTCCGGCGACGCAGTGCAGAAGATGCTGGGACACTGCACCTTCGAG
CCTGCAGAGCCAAAGGCCGCCAAGAACACCTACACAGCCGAGCGGTTTATCTGGCTGACAAAGCTGAACAATCTGAGAATCCTGG
AGCAGGGCGGATCAGGAGGCTCTGGCGGTTCAGGTGGATCAGGCGGTAGCGGAGGTTCAGGTGGTTTTGAGAGGAACTACGACC
CCCGGGAGCTGAGAAAGGAGACATACCTGCTGTATGAGATCAAGTGGGGCAAGTCCGGCAAGCTGTGGAGGCACTGGTGCCAGA
ACAATCGCACACAGCACGCCGAGGTGTACTTCCTGGAGAACATCTTTAATGCCCGGAGATTCAATCCATCTACCCACTGTAGCATCA
CATGGTATCTGAGCTGGTCCCCCTGCGCCGAGTGTTCTCAGAAGATCGTGGATTTCCTGAAGGAGCACCCTAACGTGAATCTGGAG
ATCTATGTGGCCCGGCTGTACTATCCAGAGAACGAGAGGAATAGGCAGGGCCTGCGGGATCTGGTGAATTCCGGCGTGACCATCA
GAATCATGGACCTGCCAGATTACAACTATTGCTGGAAGACCTTCGTGAGCGATCAGGGAGGCGACGAGGATTACTGGCCAGGACA
CTTCGCCCCTTGGATCAAGCAGTATAGCCTGAAGCTGGGCTCCTCTGGCTCTGAGACACCTGGCACAAGCGAGAGCGCAACACCTG
AAAGCAGCGGCGGATCCGAGAGGCCACTGACCGACACAGAGAGGGCCACCCTGATGGATGAGCCTTACCGGAAGTCTAAGCTGA
CATATGCCCAGGCCAGAAAGCTGCTGGGCCTGGAGGACACCGCCTTCTTTAAGGGCCTGAGATACGGCAAGGATAATGCCGAGGC
CTCCACACTGATGGAGATGAAGGCCTATCACGCCATCTCTCGCGCCCTGGAGAAGGAGGGCCTGAAGGACAAGAAGTCCCCCCTG
AACCTGAGCTCCGAGCTGCAGGATGAGATCGGCACCGCCTTCTCTCTGTTTAAGACCGACGAGGATATCACAGGCCGCCTGAAGG
ACAGGGTGCAGCCTGAGATCCTGGAGGCCCTGCTGAAGCACATCTCTTTCGATAAGTTTGTGCAGATCAGCCTGAAGGCCCTGAGA
AGGATCGTGCCACTGATGGAGCAGGGCAAGCGGTACGACGAGGCCTGCGCCGAGATCTACGGCGATCACTATGGCAAGAAGAAC
ACAGAGGAGAAGATCTATCTGCCCCCTATCCCTGCCGACGAGATCAGAAATCCTGTGGTGCTGAGGGCCCTGTCCCAGGCAAGAA
AAGTGATCAACGGAGTGGTGCGCCGGTACGGATCTCCAGCCCGGATCCACATCGAGACCGCCAGAGAAGTGGGCAAGAGCTTCA
AGGACCGGAAGGAGATCGAGAAGAGACAGGAGGAGAATCGCAAGGATCGGGAGAAGGCCGCCGCCAAGTTTAGGGAGTACTTC
CCTAACTTTGTGGGCGAGCCAAAGTCTAAGGACATCCTGAAGCTGCGCCTGTACGAGCAGCAGCACGGCAAGTGTCTGTATAGCG
GCAAGGAGATCAATCTGGTGCGGCTGAACGAGAAGGGCTATGTGGAGATCGATCACGCCCTGCCTTTCTCCAGAACCTGGGACGA
TTCTTTTAACAATAAGGTGCTGGTGCTGGGCAGCGAGAACCAGAATAAGGGCAATCAGACACCATACGAGTATTTCAATGGCAAG
GACAACTCCAGGGAGTGGCAGGAGTTCAAGGCCCGCGTGGAGACCTCTAGATTTCCCAGGAGCAAGAAGCAGCGGATCCTGCTG
CAGAAGTTCGACGAGGATGGCTTTAAGGAGTGCAACCTGAATGACACCAGATACGTGAACCGGTTCCTGTGCCAGTTTGTGGCCG
ATCACATCCTGCTGACCGGCAAGGGCAAGAGAAGGGTGTTCGCCTCTAATGGCCAGATCACAAACCTGCTGAGGGGATTTTGGGG
ACTGAGGAAGGTGCGGGCAGAGAATGACAGACACCACGCACTGGATGCAGTGGTGGTGGCATGCAGCACCGTGGCAATGCAGCA
GAAGATCACAAGATTCGTGAGGTATAAGGAGATGAACGCCTTTGACGGCAAGACCATCGATAAGGAGACAGGCAAGGTGCTGCA
CCAGAAGACCCACTTCCCCCAGCCTTGGGAGTTCTTTGCCCAGGAAGTGATGATCCGGGTGTTCGGCAAGCCAGACGGCAAGCCTG
AGTTTGAGGAGGCCGATACCCCAGAGAAGCTGAGGACACTGCTGGCAGAGAAGCTGTCTAGCAGGCCAGAGGCAGTGCACGAGT
ACGTGACCCCACTGTTCGTGTCCAGGGCACCCAATCGGAAGATGTCTGGCGCCCACAAGGACACACTGAGAAGCGCCAAGAGGTT
TGTGAAGCACAACGAGAAGATCTCCGTGAAGAGAGTGTGGCTGACCGAGATCAAGCTGGCCGATCTGGAGAACATGGTGAATTA
CAAGAACGGCAGGGAGATCGAGCTGTATGAGGCCCTGAAGGCAAGGCTGGAGGCCTACGGAGGAAATGCCAAGCAGGCCTTCG
ACCCAAAGGATAACCCCTTTTATAAGAAGGGAGGACAGCTGGTGAAGGCCGTGCGGGTGGAGAAGACCCAGGAGAGCGGCGTG
CTGCTGAATAAGAAGAACGCCTACACAATCGCCGACAATGGCGATATGGTGAGAGTGGACGTGTTCTGTAAGGTGGATAAGAAG
GGCAAGAATCAGTACTTTATCGTGCCTATCTATGCCTGGCAGGTGGCCGAGAACATCCTGCCAGACATCGATTGCAAGGGCTACAG
AATCGACGATAGCTATACATTCTGTTTTTCCCTGCACAAGTATGACCTGATCGCCTTCCAGAAGGATGAGAAGTCCAAGGTGGAGTT
TGCCTACTATATCAATTGCGACTCCTCTAACGGCAGGTTCTACCTGGCCTGGCACGATAAGGGCAGCAAGGAGCAGCAGTTTCGCA
TCTCCACCCAGAATCTGGTGCTGATCCAGAAGTATCAGGTGAACGAGCTGGGCAAGGAGATCAGGCCATGTCGGCTGAAGAAGCG
CCCACCCGTGCGGAGCGGAGGATCCGGAGGATCTGGAGGCAGCACCAACCTGTCTGACATCATCGAGAAGGAGACAGGCAAGCA
GCTGGTCATCCAGGAGAGCATCCTGATGCTGCCCGAAGAAGTCGAAGAAGTGATCGGAAACAAGCCTGAGAGCGATATCCTGGTC
CATACCGCCTACGACGAGAGTACCGACGAAAATGTGATGCTGCTGACATCCGACGCCCCAGAGTATAAGCCCTGGGCTCTGGTCAT
CCAGGATTCCAACGGAGAGAACAAAATCAAAATGCTGTCTGGCGGCTCAAAAAGAACCGCCGACGGCAGCGAATTCGAGCCCAAG
AAGAAGAGGAAAGTC

Nme2-evoFERNY-i7: BPSV40-NLS, Nme2Cas9, EvoFERNY, UGI, Linkers

AAACGGACAGCCGACGGAAGCGAGTTCGAGTCACCAAAGAAGAAGCGGAAAGTCGAAGATATGGCCGCCTTCAAGCCTAACCCA
ATCAATTACATCCTGGGACTGGCCATCGGAATCGCATCCGTGGGATGGGCTATGGTGGAGATCGACGAGGAGGAGAATCCTATCC
GGCTGATCGATCTGGGCGTGAGAGTGTTTGAGAGGGCCGAGGTGCCAAAGACCGGCGATTCTCTGGCTATGGCCCGGAGACTGG
CACGGAGCGTGAGGCGCCTGACACGGAGAAGGGCACACAGGCTGCTGAGGGCACGCCGGCTGCTGAAGAGAGAGGGCGTGCTG
CAGGCAGCAGACTTCGATGAGAATGGCCTGATCAAGAGCCTGCCAAACACCCCCTGGCAGCTGAGAGCAGCCGCCCTGGACAGGA
AGCTGACACCACTGGAGTGGTCTGCCGTGCTGCTGCACCTGATCAAGCACCGCGGCTACCTGAGCCAGCGGAAGAACGAGGGAG
AGACAGCAGACAAGGAGCTGGGCGCCCTGCTGAAGGGAGTGGCCAACAATGCCCACGCCCTGCAGACCGGCGATTTCAGGACAC
CTGCCGAGCTGGCCCTGAATAAGTTTGAGAAGGAGTCCGGCCACATCAGAAACCAGAGGGGCGACTATAGCCACACCTTCTCCCG
CAAGGATCTGCAGGCCGAGCTGATCCTGCTGTTCGAGAAGCAGAAGGAGTTTGGCAATCCACACGTGAGCGGAGGCCTGAAGGA
GGGAATCGAGACCCTGCTGATGACACAGAGGCCTGCCCTGTCCGGCGACGCAGTGCAGAAGATGCTGGGACACTGCACCTTCGAG
CCTGCAGAGCCAAAGGCCGCCAAGAACACCTACACAGCCGAGCGGTTTATCTGGCTGACAAAGCTGAACAATCTGAGAATCCTGG
AGCAGGGATCCGAGAGGCCACTGACCGACACAGAGAGGGCCACCCTGATGGATGAGCCTTACCGGAAGTCTAAGCTGACATATG
CCCAGGCCAGAAAGCTGCTGGGCCTGGAGGACACCGCCTTCTTTAAGGGCCTGAGATACGGCAAGGATAATGCCGAGGCCTCCAC
ACTGATGGAGATGAAGGCCTATCACGCCATCTCTCGCGCCCTGGAGAAGGAGGGCCTGAAGGACAAGAAGTCCCCCCTGAACCTG
AGCTCCGAGCTGCAGGATGAGATCGGCACCGCCTTCTCTCTGTTTAAGACCGACGAGGATATCACAGGCCGCCTGAAGGACAGGG
TGCAGCCTGAGATCCTGGAGGCCCTGCTGAAGCACATCTCTTTCGATAAGTTTGTGCAGATCAGCCTGAAGGCCCTGAGAAGGATC
GTGCCACTGATGGAGCAGGGCAAGCGGTACGACGAGGCCTGCGCCGAGATCTACGGCGATCACTATGGCAAGAAGAACACAGAG
GAGAAGATCTATCTGCCCCCTATCCCTGCCGACGAGATCAGAAATCCTGTGGTGCTGAGGGCCCTGTCCCAGGCAAGAAAAGTGAT
CAACGGAGTGGTGCGCCGGTACGGATCTCCAGCCCGGATCCACATCGAGACCGCCAGAGAAGTGGGCAAGAGCTTCAAGGACCG
GAAGGAGATCGAGAAGAGACAGGAGGAGAATCGCAAGGATCGGGAGAAGGCCGCCGCCAAGTTTAGGGAGTACTTCCCTAACTT
TGTGGGCGAGCCAAAGTCTAAGGACATCCTGAAGCTGCGCCTGTACGAGCAGCAGCACGGCAAGTGTCTGTATAGCGGCAAGGA
GATCAATCTGGTGCGGCTGAACGAGAAGGGCTATGTGGAGATCGATCACGCCCTGCCTTTCTCCAGAACCTGGGACGATTCTTTTA
ACAATAAGGTGCTGGTGCTGGGCAGCGAGAACCAGAATAAGGGCAATCAGACACCATACGAGTATTTCAATGGCAAGGACAACTC
CAGGGAGTGGCAGGAGTTCAAGGCCCGCGTGGAGACCTCTAGATTTCCCAGGAGCAAGAAGCAGCGGATCCTGCTGCAGAAGTT
CGACGAGGATGGCTTTAAGGAGTGCAACCTGAATGACACCAGATACGTGAACCGGTTCCTGTGCCAGTTTGTGGCCGATCACATCC
TGCTGACCGGCAAGGGCAAGAGAAGGGTGTTCGCCTCTAATGGCCAGATCACAAACCTGCTGAGGGGATTTTGGGGACTGAGGA
AGGTGCGGGCAGAGAATGACAGACACCACGCACTGGATGCAGTGGTGGTGGCATGCAGCACCGTGGCAATGCAGCAGAAGATCA
CAAGATTCGTGAGGTATAAGGAGATGAACGCCTTTGACGGCAAGACCATCGATAAGGAGGGCGGATCAGGAGGCTCTGGCGGTT
CAGGTGGATCAGGCGGTAGCGGAGGTTCAGGTGGTTTTGAGAGGAACTACGACCCCCGGGAGCTGAGAAAGGAGACATACCTGC
TGTATGAGATCAAGTGGGGCAAGTCCGGCAAGCTGTGGAGGCACTGGTGCCAGAACAATCGCACACAGCACGCCGAGGTGTACTT
CCTGGAGAACATCTTTAATGCCCGGAGATTCAATCCATCTACCCACTGTAGCATCACATGGTATCTGAGCTGGTCCCCCTGCGCCGA
GTGTTCTCAGAAGATCGTGGATTTCCTGAAGGAGCACCCTAACGTGAATCTGGAGATCTATGTGGCCCGGCTGTACTATCCAGAGA
ACGAGAGGAATAGGCAGGGCCTGCGGGATCTGGTGAATTCCGGCGTGACCATCAGAATCATGGACCTGCCAGATTACAACTATTG
CTGGAAGACCTTCGTGAGCGATCAGGGAGGCGACGAGGATTACTGGCCAGGACACTTCGCCCCTTGGATCAAGCAGTATAGCCTG
AAGCTGGGCTCCTCTGGCTCTGAGACACCTGGCACAAGCGAGAGCGCAACACCTGAAAGCAGCGGCACAGGCAAGGTGCTGCAC
CAGAAGACCCACTTCCCCCAGCCTTGGGAGTTCTTTGCCCAGGAAGTGATGATCCGGGTGTTCGGCAAGCCAGACGGCAAGCCTG
AGTTTGAGGAGGCCGATACCCCAGAGAAGCTGAGGACACTGCTGGCAGAGAAGCTGTCTAGCAGGCCAGAGGCAGTGCACGAGT
ACGTGACCCCACTGTTCGTGTCCAGGGCACCCAATCGGAAGATGTCTGGCGCCCACAAGGACACACTGAGAAGCGCCAAGAGGTT
TGTGAAGCACAACGAGAAGATCTCCGTGAAGAGAGTGTGGCTGACCGAGATCAAGCTGGCCGATCTGGAGAACATGGTGAATTA
CAAGAACGGCAGGGAGATCGAGCTGTATGAGGCCCTGAAGGCAAGGCTGGAGGCCTACGGAGGAAATGCCAAGCAGGCCTTCG
ACCCAAAGGATAACCCCTTTTATAAGAAGGGAGGACAGCTGGTGAAGGCCGTGCGGGTGGAGAAGACCCAGGAGAGCGGCGTG
CTGCTGAATAAGAAGAACGCCTACACAATCGCCGACAATGGCGATATGGTGAGAGTGGACGTGTTCTGTAAGGTGGATAAGAAG
GGCAAGAATCAGTACTTTATCGTGCCTATCTATGCCTGGCAGGTGGCCGAGAACATCCTGCCAGACATCGATTGCAAGGGCTACAG
AATCGACGATAGCTATACATTCTGTTTTTCCCTGCACAAGTATGACCTGATCGCCTTCCAGAAGGATGAGAAGTCCAAGGTGGAGTT
TGCCTACTATATCAATTGCGACTCCTCTAACGGCAGGTTCTACCTGGCCTGGCACGATAAGGGCAGCAAGGAGCAGCAGTTTCGCA
TCTCCACCCAGAATCTGGTGCTGATCCAGAAGTATCAGGTGAACGAGCTGGGCAAGGAGATCAGGCCATGTCGGCTGAAGAAGCG
CCCACCCGTGCGGAGCGGAGGATCCGGAGGATCTGGAGGCAGCACCAACCTGTCTGACATCATCGAGAAGGAGACAGGCAAGCA
GCTGGTCATCCAGGAGAGCATCCTGATGCTGCCCGAAGAAGTCGAAGAAGTGATCGGAAACAAGCCTGAGAGCGATATCCTGGTC
CATACCGCCTACGACGAGAGTACCGACGAAAATGTGATGCTGCTGACATCCGACGCCCCAGAGTATAAGCCCTGGGCTCTGGTCAT
CCAGGATTCCAACGGAGAGAACAAAATCAAAATGCTGTCTGGCGGCTCAAAAAGAACCGCCGACGGCAGCGAATTCGAGCCCAAG
AAGAAGAGGAAAGTC

Nme2-evoFERNY-i8: BPSV40-NLS, Nme2Cas9, EvoFERNY, UGI, Linkers

AAACGGACAGCCGACGGAAGCGAGTTCGAGTCACCAAAGAAGAAGCGGAAAGTCGAAGATATGGCCGCCTTCAAGCCTAACCCA
ATCAATTACATCCTGGGACTGGCCATCGGAATCGCATCCGTGGGATGGGCTATGGTGGAGATCGACGAGGAGGAGAATCCTATCC
GGCTGATCGATCTGGGCGTGAGAGTGTTTGAGAGGGCCGAGGTGCCAAAGACCGGCGATTCTCTGGCTATGGCCCGGAGACTGG
CACGGAGCGTGAGGCGCCTGACACGGAGAAGGGCACACAGGCTGCTGAGGGCACGCCGGCTGCTGAAGAGAGAGGGCGTGCTG
CAGGCAGCAGACTTCGATGAGAATGGCCTGATCAAGAGCCTGCCAAACACCCCCTGGCAGCTGAGAGCAGCCGCCCTGGACAGGA
AGCTGACACCACTGGAGTGGTCTGCCGTGCTGCTGCACCTGATCAAGCACCGCGGCTACCTGAGCCAGCGGAAGAACGAGGGAG
AGACAGCAGACAAGGAGCTGGGCGCCCTGCTGAAGGGAGTGGCCAACAATGCCCACGCCCTGCAGACCGGCGATTTCAGGACAC
CTGCCGAGCTGGCCCTGAATAAGTTTGAGAAGGAGTCCGGCCACATCAGAAACCAGAGGGGCGACTATAGCCACACCTTCTCCCG
CAAGGATCTGCAGGCCGAGCTGATCCTGCTGTTCGAGAAGCAGAAGGAGTTTGGCAATCCACACGTGAGCGGAGGCCTGAAGGA
GGGAATCGAGACCCTGCTGATGACACAGAGGCCTGCCCTGTCCGGCGACGCAGTGCAGAAGATGCTGGGACACTGCACCTTCGAG
CCTGCAGAGCCAAAGGCCGCCAAGAACACCTACACAGCCGAGCGGTTTATCTGGCTGACAAAGCTGAACAATCTGAGAATCCTGG
AGCAGGGATCCGAGAGGCCACTGACCGACACAGAGAGGGCCACCCTGATGGATGAGCCTTACCGGAAGTCTAAGCTGACATATG
CCCAGGCCAGAAAGCTGCTGGGCCTGGAGGACACCGCCTTCTTTAAGGGCCTGAGATACGGCAAGGATAATGCCGAGGCCTCCAC
ACTGATGGAGATGAAGGCCTATCACGCCATCTCTCGCGCCCTGGAGAAGGAGGGCCTGAAGGACAAGAAGTCCCCCCTGAACCTG
AGCTCCGAGCTGCAGGATGAGATCGGCACCGCCTTCTCTCTGTTTAAGACCGACGAGGATATCACAGGCCGCCTGAAGGACAGGG
TGCAGCCTGAGATCCTGGAGGCCCTGCTGAAGCACATCTCTTTCGATAAGTTTGTGCAGATCAGCCTGAAGGCCCTGAGAAGGATC
GTGCCACTGATGGAGCAGGGCAAGCGGTACGACGAGGCCTGCGCCGAGATCTACGGCGATCACTATGGCAAGAAGAACACAGAG
GAGAAGATCTATCTGCCCCCTATCCCTGCCGACGAGATCAGAAATCCTGTGGTGCTGAGGGCCCTGTCCCAGGCAAGAAAAGTGAT
CAACGGAGTGGTGCGCCGGTACGGATCTCCAGCCCGGATCCACATCGAGACCGCCAGAGAAGTGGGCAAGAGCTTCAAGGACCG
GAAGGAGATCGAGAAGAGACAGGAGGAGAATCGCAAGGATCGGGAGAAGGCCGCCGCCAAGTTTAGGGAGTACTTCCCTAACTT
TGTGGGCGAGCCAAAGTCTAAGGACATCCTGAAGCTGCGCCTGTACGAGCAGCAGCACGGCAAGTGTCTGTATAGCGGCAAGGA
GATCAATCTGGTGCGGCTGAACGAGAAGGGCTATGTGGAGATCGATCACGCCCTGCCTTTCTCCAGAACCTGGGACGATTCTTTTA
ACAATAAGGTGCTGGTGCTGGGCAGCGAGAACCAGAATAAGGGCAATCAGACACCATACGAGTATTTCAATGGCAAGGACAACTC
CAGGGAGTGGCAGGAGTTCAAGGCCCGCGTGGAGACCTCTAGATTTCCCAGGAGCAAGAAGCAGCGGATCCTGCTGCAGAAGTT
CGACGAGGATGGCTTTAAGGAGTGCAACCTGAATGACACCAGATACGTGAACCGGTTCCTGTGCCAGTTTGTGGCCGATCACATCC
TGCTGACCGGCAAGGGCAAGAGAAGGGTGTTCGCCTCTAATGGCCAGATCACAAACCTGCTGAGGGGATTTTGGGGACTGAGGA
AGGTGCGGGCAGAGAATGACAGACACCACGCACTGGATGCAGTGGTGGTGGCATGCAGCACCGTGGCAATGCAGCAGAAGATCA
CAAGATTCGTGAGGTATAAGGAGATGAACGCCTTTGACGGCAAGACCATCGATAAGGAGACAGGCAAGGTGCTGCACCAGAAGA
CCCACTTCCCCCAGCCTTGGGAGTTCTTTGCCCAGGAAGTGATGATCCGGGTGTTCGGCAAGCCAGACGGCAAGCCTGGCGGATCA
GGAGGCTCTGGCGGTTCAGGTGGATCAGGCGGTAGCGGAGGTTCAGGTGGTTTTGAGAGGAACTACGACCCCCGGGAGCTGAGA
AAGGAGACATACCTGCTGTATGAGATCAAGTGGGGCAAGTCCGGCAAGCTGTGGAGGCACTGGTGCCAGAACAATCGCACACAG
CACGCCGAGGTGTACTTCCTGGAGAACATCTTTAATGCCCGGAGATTCAATCCATCTACCCACTGTAGCATCACATGGTATCTGAGC
TGGTCCCCCTGCGCCGAGTGTTCTCAGAAGATCGTGGATTTCCTGAAGGAGCACCCTAACGTGAATCTGGAGATCTATGTGGCCCG
GCTGTACTATCCAGAGAACGAGAGGAATAGGCAGGGCCTGCGGGATCTGGTGAATTCCGGCGTGACCATCAGAATCATGGACCTG
CCAGATTACAACTATTGCTGGAAGACCTTCGTGAGCGATCAGGGAGGCGACGAGGATTACTGGCCAGGACACTTCGCCCCTTGGA
TCAAGCAGTATAGCCTGAAGCTGGGCTCCTCTGGCTCTGAGACACCTGGCACAAGCGAGAGCGCAACACCTGAAAGCAGCGGCGA
GTTTGAGGAGGCCGATACCCCAGAGAAGCTGAGGACACTGCTGGCAGAGAAGCTGTCTAGCAGGCCAGAGGCAGTGCACGAGTA
CGTGACCCCACTGTTCGTGTCCAGGGCACCCAATCGGAAGATGTCTGGCGCCCACAAGGACACACTGAGAAGCGCCAAGAGGTTT
GTGAAGCACAACGAGAAGATCTCCGTGAAGAGAGTGTGGCTGACCGAGATCAAGCTGGCCGATCTGGAGAACATGGTGAATTAC
AAGAACGGCAGGGAGATCGAGCTGTATGAGGCCCTGAAGGCAAGGCTGGAGGCCTACGGAGGAAATGCCAAGCAGGCCTTCGA
CCCAAAGGATAACCCCTTTTATAAGAAGGGAGGACAGCTGGTGAAGGCCGTGCGGGTGGAGAAGACCCAGGAGAGCGGCGTGCT
GCTGAATAAGAAGAACGCCTACACAATCGCCGACAATGGCGATATGGTGAGAGTGGACGTGTTCTGTAAGGTGGATAAGAAGGG
CAAGAATCAGTACTTTATCGTGCCTATCTATGCCTGGCAGGTGGCCGAGAACATCCTGCCAGACATCGATTGCAAGGGCTACAGAA
TCGACGATAGCTATACATTCTGTTTTTCCCTGCACAAGTATGACCTGATCGCCTTCCAGAAGGATGAGAAGTCCAAGGTGGAGTTTG
CCTACTATATCAATTGCGACTCCTCTAACGGCAGGTTCTACCTGGCCTGGCACGATAAGGGCAGCAAGGAGCAGCAGTTTCGCATC
TCCACCCAGAATCTGGTGCTGATCCAGAAGTATCAGGTGAACGAGCTGGGCAAGGAGATCAGGCCATGTCGGCTGAAGAAGCGCC
CACCCGTGCGGAGCGGAGGATCCGGAGGATCTGGAGGCAGCACCAACCTGTCTGACATCATCGAGAAGGAGACAGGCAAGCAGC
TGGTCATCCAGGAGAGCATCCTGATGCTGCCCGAAGAAGTCGAAGAAGTGATCGGAAACAAGCCTGAGAGCGATATCCTGGTCCA
TACCGCCTACGACGAGAGTACCGACGAAAATGTGATGCTGCTGACATCCGACGCCCCAGAGTATAAGCCCTGGGCTCTGGTCATCC
AGGATTCCAACGGAGAGAACAAAATCAAAATGCTGTCTGGCGGCTCAAAAAGAACCGCCGACGGCAGCGAATTCGAGCCCAAGA
AGAAGAGGAAAGTC

Nme2-rAPOBEC1-nt: BPSV40-NLS, Nme2Cas9, rAPOBEC1, UGI, Linkers

AAACGGACAGCCGACGGAAGCGAGTTCGAGTCACCAAAGAAGAAGCGGAAAGTCAGTAGCTCAGAGACTGGCCCAGTGGCTGTG
GACCCCACATTGAGACGGCGGATCGAGCCCCATGAGTTTGAGGTATTCTTCGATCCGAGAGAGCTCCGCAAGGAGACCTGCCTGCT
TTACGAAATTAATTGGGGGGGCCGGCACTCCATTTGGCGACATACATCACAGAACACTAACAAGCACGTCGAAGTCAACTTCATCG
AGAAGTTCACGACAGAAAGATATTTCTGTCCGAACACAAGGTGCAGCATTACCTGGTTTCTCAGCTGGAGCCCATGCGGCGAATGT
AGTAGGGCCATCACTGAATTCCTGTCAAGGTATCCCCACGTCACTCTGTTTATTTACATCGCAAGGCTGTACCACCACGCTGACCCCC
GCAATCGACAAGGCCTGCGGGATTTGATCTCTTCAGGTGTGACTATCCAAATTATGACTGAGCAGGAGTCAGGATACTGCTGGAGA
AACTTTGTGAATTATAGCCCGAGTAATGAAGCCCACTGGCCTAGGTATCCCCATCTGTGGGTACGACTGTACGTTCTTGAACTGTAC
TGCATCATACTGGGCCTGCCTCCTTGTCTCAACATTCTGAGAAGGAAGCAGCCACAGCTGACATTCTTTACCATCGCTCTTCAGTCTT
GTCATTACCAGCGACTGCCCCCACACATTCTCTGGGCCACCGGGTTGAAATCTGGCGGATCTAGCGGAGGATCCTCTGGCAGCGAG
ACACCAGGAACAAGCGAGTCAGCAACACCAGAGAGCAGTGGCGGCAGCAGCGGCGGCAGCGCCGCCTTCAAGCCTAACCCAATC
AATTACATCCTGGGACTGGCCATCGGAATCGCATCCGTGGGATGGGCTATGGTGGAGATCGACGAGGAGGAGAATCCTATCCGGC
TGATCGATCTGGGCGTGAGAGTGTTTGAGAGGGCCGAGGTGCCAAAGACCGGCGATTCTCTGGCTATGGCCCGGAGACTGGCAC
GGAGCGTGAGGCGCCTGACACGGAGAAGGGCACACAGGCTGCTGAGGGCACGCCGGCTGCTGAAGAGAGAGGGCGTGCTGCAG
GCAGCAGACTTCGATGAGAATGGCCTGATCAAGAGCCTGCCAAACACCCCCTGGCAGCTGAGAGCAGCCGCCCTGGACAGGAAGC
TGACACCACTGGAGTGGTCTGCCGTGCTGCTGCACCTGATCAAGCACCGCGGCTACCTGAGCCAGCGGAAGAACGAGGGAGAGA
CAGCAGACAAGGAGCTGGGCGCCCTGCTGAAGGGAGTGGCCAACAATGCCCACGCCCTGCAGACCGGCGATTTCAGGACACCTG
CCGAGCTGGCCCTGAATAAGTTTGAGAAGGAGTCCGGCCACATCAGAAACCAGAGGGGCGACTATAGCCACACCTTCTCCCGCAA
GGATCTGCAGGCCGAGCTGATCCTGCTGTTCGAGAAGCAGAAGGAGTTTGGCAATCCACACGTGAGCGGAGGCCTGAAGGAGGG
AATCGAGACCCTGCTGATGACACAGAGGCCTGCCCTGTCCGGCGACGCAGTGCAGAAGATGCTGGGACACTGCACCTTCGAGCCT
GCAGAGCCAAAGGCCGCCAAGAACACCTACACAGCCGAGCGGTTTATCTGGCTGACAAAGCTGAACAATCTGAGAATCCTGGAGC
AGGGATCCGAGAGGCCACTGACCGACACAGAGAGGGCCACCCTGATGGATGAGCCTTACCGGAAGTCTAAGCTGACATATGCCCA
GGCCAGAAAGCTGCTGGGCCTGGAGGACACCGCCTTCTTTAAGGGCCTGAGATACGGCAAGGATAATGCCGAGGCCTCCACACTG
ATGGAGATGAAGGCCTATCACGCCATCTCTCGCGCCCTGGAGAAGGAGGGCCTGAAGGACAAGAAGTCCCCCCTGAACCTGAGCT
CCGAGCTGCAGGATGAGATCGGCACCGCCTTCTCTCTGTTTAAGACCGACGAGGATATCACAGGCCGCCTGAAGGACAGGGTGCA
GCCTGAGATCCTGGAGGCCCTGCTGAAGCACATCTCTTTCGATAAGTTTGTGCAGATCAGCCTGAAGGCCCTGAGAAGGATCGTGC
CACTGATGGAGCAGGGCAAGCGGTACGACGAGGCCTGCGCCGAGATCTACGGCGATCACTATGGCAAGAAGAACACAGAGGAG
AAGATCTATCTGCCCCCTATCCCTGCCGACGAGATCAGAAATCCTGTGGTGCTGAGGGCCCTGTCCCAGGCAAGAAAAGTGATCAA
CGGAGTGGTGCGCCGGTACGGATCTCCAGCCCGGATCCACATCGAGACCGCCAGAGAAGTGGGCAAGAGCTTCAAGGACCGGAA
GGAGATCGAGAAGAGACAGGAGGAGAATCGCAAGGATCGGGAGAAGGCCGCCGCCAAGTTTAGGGAGTACTTCCCTAACTTTGT
GGGCGAGCCAAAGTCTAAGGACATCCTGAAGCTGCGCCTGTACGAGCAGCAGCACGGCAAGTGTCTGTATAGCGGCAAGGAGAT
CAATCTGGTGCGGCTGAACGAGAAGGGCTATGTGGAGATCGATCACGCCCTGCCTTTCTCCAGAACCTGGGACGATTCTTTTAACA
ATAAGGTGCTGGTGCTGGGCAGCGAGAACCAGAATAAGGGCAATCAGACACCATACGAGTATTTCAATGGCAAGGACAACTCCAG
GGAGTGGCAGGAGTTCAAGGCCCGCGTGGAGACCTCTAGATTTCCCAGGAGCAAGAAGCAGCGGATCCTGCTGCAGAAGTTCGA
CGAGGATGGCTTTAAGGAGTGCAACCTGAATGACACCAGATACGTGAACCGGTTCCTGTGCCAGTTTGTGGCCGATCACATCCTGC
TGACCGGCAAGGGCAAGAGAAGGGTGTTCGCCTCTAATGGCCAGATCACAAACCTGCTGAGGGGATTTTGGGGACTGAGGAAGG
TGCGGGCAGAGAATGACAGACACCACGCACTGGATGCAGTGGTGGTGGCATGCAGCACCGTGGCAATGCAGCAGAAGATCACAA
GATTCGTGAGGTATAAGGAGATGAACGCCTTTGACGGCAAGACCATCGATAAGGAGACAGGCAAGGTGCTGCACCAGAAGACCC
ACTTCCCCCAGCCTTGGGAGTTCTTTGCCCAGGAAGTGATGATCCGGGTGTTCGGCAAGCCAGACGGCAAGCCTGAGTTTGAGGA
GGCCGATACCCCAGAGAAGCTGAGGACACTGCTGGCAGAGAAGCTGTCTAGCAGGCCAGAGGCAGTGCACGAGTACGTGACCCC
ACTGTTCGTGTCCAGGGCACCCAATCGGAAGATGTCTGGCGCCCACAAGGACACACTGAGAAGCGCCAAGAGGTTTGTGAAGCAC
AACGAGAAGATCTCCGTGAAGAGAGTGTGGCTGACCGAGATCAAGCTGGCCGATCTGGAGAACATGGTGAATTACAAGAACGGC
AGGGAGATCGAGCTGTATGAGGCCCTGAAGGCAAGGCTGGAGGCCTACGGAGGAAATGCCAAGCAGGCCTTCGACCCAAAGGAT
AACCCCTTTTATAAGAAGGGAGGACAGCTGGTGAAGGCCGTGCGGGTGGAGAAGACCCAGGAGAGCGGCGTGCTGCTGAATAAG
AAGAACGCCTACACAATCGCCGACAATGGCGATATGGTGAGAGTGGACGTGTTCTGTAAGGTGGATAAGAAGGGCAAGAATCAG
TACTTTATCGTGCCTATCTATGCCTGGCAGGTGGCCGAGAACATCCTGCCAGACATCGATTGCAAGGGCTACAGAATCGACGATAG
CTATACATTCTGTTTTTCCCTGCACAAGTATGACCTGATCGCCTTCCAGAAGGATGAGAAGTCCAAGGTGGAGTTTGCCTACTATAT
CAATTGCGACTCCTCTAACGGCAGGTTCTACCTGGCCTGGCACGATAAGGGCAGCAAGGAGCAGCAGTTTCGCATCTCCACCCAGA
ATCTGGTGCTGATCCAGAAGTATCAGGTGAACGAGCTGGGCAAGGAGATCAGGCCATGTCGGCTGAAGAAGCGCCCACCCGTGC
GGAGCGGAGGATCCGGAGGATCTGGAGGCAGCACCAACCTGTCTGACATCATCGAGAAGGAGACAGGCAAGCAGCTGGTCATCC
AGGAGAGCATCCTGATGCTGCCCGAAGAAGTCGAAGAAGTGATCGGAAACAAGCCTGAGAGCGATATCCTGGTCCATACCGCCTA
CGACGAGAGTACCGACGAAAATGTGATGCTGCTGACATCCGACGCCCCAGAGTATAAGCCCTGGGCTCTGGTCATCCAGGATTCCA
ACGGAGAGAACAAAATCAAAATGCTGTCTGGCGGCTCAAAAAGAACCGCCGACGGCAGCGAATTCGAGCCCAAGAAGAAGAGGA
AAGTC

Nme2-rAPOBEC1-i1: BPSV40-NLS, Nme2Cas9, rAPOBEC1, UGI, Linkers

AAACGGACAGCCGACGGAAGCGAGTTCGAGTCACCAAAGAAGAAGCGGAAAGTCGAAGATATGGCCGCCTTCAAGCCTAACCCA
ATCAATTACATCCTGGGACTGGCCATCGGAATCGCATCCGTGGGATGGGCTATGGTGGAGATCGACGAGGAGGAGAATCCTATCC
GGCTGATCGATCTGGGCGTGAGAGTGTTTGAGAGGGCCGAGGTGCCAAAGACCGGCGATTCTCTGGCTATGGCCCGGAGACTGG
CACGGAGCGTGAGGCGCCTGACACGGAGAAGGGCACACAGGCTGCTGAGGGCACGCCGGCTGCTGAAGAGAGAGGGCGTGCTG
CAGGCAGCAGACTTCGATGAGAATGGCCTGATCAAGAGCCTGCCAAACACCCCCTGGCAGCTGAGAGCAGCCGCCCTGGACAGGA
AGCTGACACCACTGGAGTGGTCTGCCGTGCTGCTGCACCTGATCAAGCACCGCGGCTACCTGAGCCAGCGGAAGAACGAGGGAG
AGACAGCAGACAAGGAGCTGGGCGCCCTGCTGAAGGGAGTGGCCAACAATGCCCACGCCCTGCAGACCGGCGATTTCAGGACAC
CTGCCGAGCTGGCCCTGAATAAGTTTGAGAAGGAGTCCGGCCACATCAGAAACCAGAGGGGCGACTATAGCCACACCTTCTCCCG
CAAGGATCTGCAGGCCGAGCTGATCCTGCTGTTCGAGAAGCAGAAGGAGTTTGGCAATCCACACGTGAGCGGAGGCCTGAAGGA
GGGAATCGAGACCCTGCTGATGACACAGAGGCCTGCCCTGTCCGGCGACGCAGTGCAGAAGATGCTGGGACACTGCACCTTCGAG
CCTGCAGAGCCAAAGGCCGCCAAGAACACCTACACAGCCGAGCGGTTTATCTGGCTGACAAAGCTGAACAATCTGAGAATCCTGG
AGCAGGGCGGATCAGGAGGCTCTGGCGGTTCAGGTGGATCAGGCGGTAGCGGAGGTTCAGGTGGTAGCTCAGAGACTGGCCCA
GTGGCTGTGGACCCCACATTGAGACGGCGGATCGAGCCCCATGAGTTTGAGGTATTCTTCGATCCGAGAGAGCTCCGCAAGGAGA
CCTGCCTGCTTTACGAAATTAATTGGGGGGGCCGGCACTCCATTTGGCGACATACATCACAGAACACTAACAAGCACGTCGAAGTC
AACTTCATCGAGAAGTTCACGACAGAAAGATATTTCTGTCCGAACACAAGGTGCAGCATTACCTGGTTTCTCAGCTGGAGCCCATGC
GGCGAATGTAGTAGGGCCATCACTGAATTCCTGTCAAGGTATCCCCACGTCACTCTGTTTATTTACATCGCAAGGCTGTACCACCAC
GCTGACCCCCGCAATCGACAAGGCCTGCGGGATTTGATCTCTTCAGGTGTGACTATCCAAATTATGACTGAGCAGGAGTCAGGATA
CTGCTGGAGAAACTTTGTGAATTATAGCCCGAGTAATGAAGCCCACTGGCCTAGGTATCCCCATCTGTGGGTACGACTGTACGTTCT
TGAACTGTACTGCATCATACTGGGCCTGCCTCCTTGTCTCAACATTCTGAGAAGGAAGCAGCCACAGCTGACATTCTTTACCATCGC
TCTTCAGTCTTGTCATTACCAGCGACTGCCCCCACACATTCTCTGGGCCACCGGGTTGAAAGGCTCCTCTGGCTCTGAGACACCTGG
CACAAGCGAGAGCGCAACACCTGAAAGCAGCGGCGGATCCGAGAGGCCACTGACCGACACAGAGAGGGCCACCCTGATGGATGA
GCCTTACCGGAAGTCTAAGCTGACATATGCCCAGGCCAGAAAGCTGCTGGGCCTGGAGGACACCGCCTTCTTTAAGGGCCTGAGA
TACGGCAAGGATAATGCCGAGGCCTCCACACTGATGGAGATGAAGGCCTATCACGCCATCTCTCGCGCCCTGGAGAAGGAGGGCC
TGAAGGACAAGAAGTCCCCCCTGAACCTGAGCTCCGAGCTGCAGGATGAGATCGGCACCGCCTTCTCTCTGTTTAAGACCGACGAG
GATATCACAGGCCGCCTGAAGGACAGGGTGCAGCCTGAGATCCTGGAGGCCCTGCTGAAGCACATCTCTTTCGATAAGTTTGTGCA
GATCAGCCTGAAGGCCCTGAGAAGGATCGTGCCACTGATGGAGCAGGGCAAGCGGTACGACGAGGCCTGCGCCGAGATCTACGG
CGATCACTATGGCAAGAAGAACACAGAGGAGAAGATCTATCTGCCCCCTATCCCTGCCGACGAGATCAGAAATCCTGTGGTGCTGA
GGGCCCTGTCCCAGGCAAGAAAAGTGATCAACGGAGTGGTGCGCCGGTACGGATCTCCAGCCCGGATCCACATCGAGACCGCCAG
AGAAGTGGGCAAGAGCTTCAAGGACCGGAAGGAGATCGAGAAGAGACAGGAGGAGAATCGCAAGGATCGGGAGAAGGCCGCC
GCCAAGTTTAGGGAGTACTTCCCTAACTTTGTGGGCGAGCCAAAGTCTAAGGACATCCTGAAGCTGCGCCTGTACGAGCAGCAGCA
CGGCAAGTGTCTGTATAGCGGCAAGGAGATCAATCTGGTGCGGCTGAACGAGAAGGGCTATGTGGAGATCGATCACGCCCTGCCT
TTCTCCAGAACCTGGGACGATTCTTTTAACAATAAGGTGCTGGTGCTGGGCAGCGAGAACCAGAATAAGGGCAATCAGACACCATA
CGAGTATTTCAATGGCAAGGACAACTCCAGGGAGTGGCAGGAGTTCAAGGCCCGCGTGGAGACCTCTAGATTTCCCAGGAGCAAG
AAGCAGCGGATCCTGCTGCAGAAGTTCGACGAGGATGGCTTTAAGGAGTGCAACCTGAATGACACCAGATACGTGAACCGGTTCC
TGTGCCAGTTTGTGGCCGATCACATCCTGCTGACCGGCAAGGGCAAGAGAAGGGTGTTCGCCTCTAATGGCCAGATCACAAACCTG
CTGAGGGGATTTTGGGGACTGAGGAAGGTGCGGGCAGAGAATGACAGACACCACGCACTGGATGCAGTGGTGGTGGCATGCAG
CACCGTGGCAATGCAGCAGAAGATCACAAGATTCGTGAGGTATAAGGAGATGAACGCCTTTGACGGCAAGACCATCGATAAGGA
GACAGGCAAGGTGCTGCACCAGAAGACCCACTTCCCCCAGCCTTGGGAGTTCTTTGCCCAGGAAGTGATGATCCGGGTGTTCGGC
AAGCCAGACGGCAAGCCTGAGTTTGAGGAGGCCGATACCCCAGAGAAGCTGAGGACACTGCTGGCAGAGAAGCTGTCTAGCAGG
CCAGAGGCAGTGCACGAGTACGTGACCCCACTGTTCGTGTCCAGGGCACCCAATCGGAAGATGTCTGGCGCCCACAAGGACACAC
TGAGAAGCGCCAAGAGGTTTGTGAAGCACAACGAGAAGATCTCCGTGAAGAGAGTGTGGCTGACCGAGATCAAGCTGGCCGATC
TGGAGAACATGGTGAATTACAAGAACGGCAGGGAGATCGAGCTGTATGAGGCCCTGAAGGCAAGGCTGGAGGCCTACGGAGGA
AATGCCAAGCAGGCCTTCGACCCAAAGGATAACCCCTTTTATAAGAAGGGAGGACAGCTGGTGAAGGCCGTGCGGGTGGAGAAG
ACCCAGGAGAGCGGCGTGCTGCTGAATAAGAAGAACGCCTACACAATCGCCGACAATGGCGATATGGTGAGAGTGGACGTGTTCT
GTAAGGTGGATAAGAAGGGCAAGAATCAGTACTTTATCGTGCCTATCTATGCCTGGCAGGTGGCCGAGAACATCCTGCCAGACAT
CGATTGCAAGGGCTACAGAATCGACGATAGCTATACATTCTGTTTTTCCCTGCACAAGTATGACCTGATCGCCTTCCAGAAGGATGA
GAAGTCCAAGGTGGAGTTTGCCTACTATATCAATTGCGACTCCTCTAACGGCAGGTTCTACCTGGCCTGGCACGATAAGGGCAGCA
AGGAGCAGCAGTTTCGCATCTCCACCCAGAATCTGGTGCTGATCCAGAAGTATCAGGTGAACGAGCTGGGCAAGGAGATCAGGCC
ATGTCGGCTGAAGAAGCGCCCACCCGTGCGGAGCGGAGGATCCGGAGGATCTGGAGGCAGCACCAACCTGTCTGACATCATCGA
GAAGGAGACAGGCAAGCAGCTGGTCATCCAGGAGAGCATCCTGATGCTGCCCGAAGAAGTCGAAGAAGTGATCGGAAACAAGCC
TGAGAGCGATATCCTGGTCCATACCGCCTACGACGAGAGTACCGACGAAAATGTGATGCTGCTGACATCCGACGCCCCAGAGTATA
AGCCCTGGGCTCTGGTCATCCAGGATTCCAACGGAGAGAACAAAATCAAAATGCTGTCTGGCGGCTCAAAAAGAACCGCCGACGG
CAGCGAATTCGAGCCCAAGAAGAAGAGGAAAGTC

Nme2-rAPOBEC1-i7: BPSV40-NLS, Nme2Cas9, rAPOBEC1, UGI, Linkers

AAACGGACAGCCGACGGAAGCGAGTTCGAGTCACCAAAGAAGAAGCGGAAAGTCGAAGATATGGCCGCCTTCAAGCCTAACCCA
ATCAATTACATCCTGGGACTGGCCATCGGAATCGCATCCGTGGGATGGGCTATGGTGGAGATCGACGAGGAGGAGAATCCTATCC
GGCTGATCGATCTGGGCGTGAGAGTGTTTGAGAGGGCCGAGGTGCCAAAGACCGGCGATTCTCTGGCTATGGCCCGGAGACTGG
CACGGAGCGTGAGGCGCCTGACACGGAGAAGGGCACACAGGCTGCTGAGGGCACGCCGGCTGCTGAAGAGAGAGGGCGTGCTG
CAGGCAGCAGACTTCGATGAGAATGGCCTGATCAAGAGCCTGCCAAACACCCCCTGGCAGCTGAGAGCAGCCGCCCTGGACAGGA
AGCTGACACCACTGGAGTGGTCTGCCGTGCTGCTGCACCTGATCAAGCACCGCGGCTACCTGAGCCAGCGGAAGAACGAGGGAG
AGACAGCAGACAAGGAGCTGGGCGCCCTGCTGAAGGGAGTGGCCAACAATGCCCACGCCCTGCAGACCGGCGATTTCAGGACAC
CTGCCGAGCTGGCCCTGAATAAGTTTGAGAAGGAGTCCGGCCACATCAGAAACCAGAGGGGCGACTATAGCCACACCTTCTCCCG
CAAGGATCTGCAGGCCGAGCTGATCCTGCTGTTCGAGAAGCAGAAGGAGTTTGGCAATCCACACGTGAGCGGAGGCCTGAAGGA
GGGAATCGAGACCCTGCTGATGACACAGAGGCCTGCCCTGTCCGGCGACGCAGTGCAGAAGATGCTGGGACACTGCACCTTCGAG
CCTGCAGAGCCAAAGGCCGCCAAGAACACCTACACAGCCGAGCGGTTTATCTGGCTGACAAAGCTGAACAATCTGAGAATCCTGG
AGCAGGGATCCGAGAGGCCACTGACCGACACAGAGAGGGCCACCCTGATGGATGAGCCTTACCGGAAGTCTAAGCTGACATATG
CCCAGGCCAGAAAGCTGCTGGGCCTGGAGGACACCGCCTTCTTTAAGGGCCTGAGATACGGCAAGGATAATGCCGAGGCCTCCAC
ACTGATGGAGATGAAGGCCTATCACGCCATCTCTCGCGCCCTGGAGAAGGAGGGCCTGAAGGACAAGAAGTCCCCCCTGAACCTG
AGCTCCGAGCTGCAGGATGAGATCGGCACCGCCTTCTCTCTGTTTAAGACCGACGAGGATATCACAGGCCGCCTGAAGGACAGGG
TGCAGCCTGAGATCCTGGAGGCCCTGCTGAAGCACATCTCTTTCGATAAGTTTGTGCAGATCAGCCTGAAGGCCCTGAGAAGGATC
GTGCCACTGATGGAGCAGGGCAAGCGGTACGACGAGGCCTGCGCCGAGATCTACGGCGATCACTATGGCAAGAAGAACACAGAG
GAGAAGATCTATCTGCCCCCTATCCCTGCCGACGAGATCAGAAATCCTGTGGTGCTGAGGGCCCTGTCCCAGGCAAGAAAAGTGAT
CAACGGAGTGGTGCGCCGGTACGGATCTCCAGCCCGGATCCACATCGAGACCGCCAGAGAAGTGGGCAAGAGCTTCAAGGACCG
GAAGGAGATCGAGAAGAGACAGGAGGAGAATCGCAAGGATCGGGAGAAGGCCGCCGCCAAGTTTAGGGAGTACTTCCCTAACTT
TGTGGGCGAGCCAAAGTCTAAGGACATCCTGAAGCTGCGCCTGTACGAGCAGCAGCACGGCAAGTGTCTGTATAGCGGCAAGGA
GATCAATCTGGTGCGGCTGAACGAGAAGGGCTATGTGGAGATCGATCACGCCCTGCCTTTCTCCAGAACCTGGGACGATTCTTTTA
ACAATAAGGTGCTGGTGCTGGGCAGCGAGAACCAGAATAAGGGCAATCAGACACCATACGAGTATTTCAATGGCAAGGACAACTC
CAGGGAGTGGCAGGAGTTCAAGGCCCGCGTGGAGACCTCTAGATTTCCCAGGAGCAAGAAGCAGCGGATCCTGCTGCAGAAGTT
CGACGAGGATGGCTTTAAGGAGTGCAACCTGAATGACACCAGATACGTGAACCGGTTCCTGTGCCAGTTTGTGGCCGATCACATCC
TGCTGACCGGCAAGGGCAAGAGAAGGGTGTTCGCCTCTAATGGCCAGATCACAAACCTGCTGAGGGGATTTTGGGGACTGAGGA
AGGTGCGGGCAGAGAATGACAGACACCACGCACTGGATGCAGTGGTGGTGGCATGCAGCACCGTGGCAATGCAGCAGAAGATCA
CAAGATTCGTGAGGTATAAGGAGATGAACGCCTTTGACGGCAAGACCATCGATAAGGAGGGCGGATCAGGAGGCTCTGGCGGTT
CAGGTGGATCAGGCGGTAGCGGAGGTTCAGGTGGTAGCTCAGAGACTGGCCCAGTGGCTGTGGACCCCACATTGAGACGGCGGA
TCGAGCCCCATGAGTTTGAGGTATTCTTCGATCCGAGAGAGCTCCGCAAGGAGACCTGCCTGCTTTACGAAATTAATTGGGGGGGC
CGGCACTCCATTTGGCGACATACATCACAGAACACTAACAAGCACGTCGAAGTCAACTTCATCGAGAAGTTCACGACAGAAAGATA
TTTCTGTCCGAACACAAGGTGCAGCATTACCTGGTTTCTCAGCTGGAGCCCATGCGGCGAATGTAGTAGGGCCATCACTGAATTCCT
GTCAAGGTATCCCCACGTCACTCTGTTTATTTACATCGCAAGGCTGTACCACCACGCTGACCCCCGCAATCGACAAGGCCTGCGGGA
TTTGATCTCTTCAGGTGTGACTATCCAAATTATGACTGAGCAGGAGTCAGGATACTGCTGGAGAAACTTTGTGAATTATAGCCCGA
GTAATGAAGCCCACTGGCCTAGGTATCCCCATCTGTGGGTACGACTGTACGTTCTTGAACTGTACTGCATCATACTGGGCCTGCCTC
CTTGTCTCAACATTCTGAGAAGGAAGCAGCCACAGCTGACATTCTTTACCATCGCTCTTCAGTCTTGTCATTACCAGCGACTGCCCCC
ACACATTCTCTGGGCCACCGGGTTGAAAGGCTCCTCTGGCTCTGAGACACCTGGCACAAGCGAGAGCGCAACACCTGAAAGCAGC
GGCACAGGCAAGGTGCTGCACCAGAAGACCCACTTCCCCCAGCCTTGGGAGTTCTTTGCCCAGGAAGTGATGATCCGGGTGTTCG
GCAAGCCAGACGGCAAGCCTGAGTTTGAGGAGGCCGATACCCCAGAGAAGCTGAGGACACTGCTGGCAGAGAAGCTGTCTAGCA
GGCCAGAGGCAGTGCACGAGTACGTGACCCCACTGTTCGTGTCCAGGGCACCCAATCGGAAGATGTCTGGCGCCCACAAGGACAC
ACTGAGAAGCGCCAAGAGGTTTGTGAAGCACAACGAGAAGATCTCCGTGAAGAGAGTGTGGCTGACCGAGATCAAGCTGGCCGA
TCTGGAGAACATGGTGAATTACAAGAACGGCAGGGAGATCGAGCTGTATGAGGCCCTGAAGGCAAGGCTGGAGGCCTACGGAG
GAAATGCCAAGCAGGCCTTCGACCCAAAGGATAACCCCTTTTATAAGAAGGGAGGACAGCTGGTGAAGGCCGTGCGGGTGGAGA
AGACCCAGGAGAGCGGCGTGCTGCTGAATAAGAAGAACGCCTACACAATCGCCGACAATGGCGATATGGTGAGAGTGGACGTGT
TCTGTAAGGTGGATAAGAAGGGCAAGAATCAGTACTTTATCGTGCCTATCTATGCCTGGCAGGTGGCCGAGAACATCCTGCCAGAC
ATCGATTGCAAGGGCTACAGAATCGACGATAGCTATACATTCTGTTTTTCCCTGCACAAGTATGACCTGATCGCCTTCCAGAAGGAT
GAGAAGTCCAAGGTGGAGTTTGCCTACTATATCAATTGCGACTCCTCTAACGGCAGGTTCTACCTGGCCTGGCACGATAAGGGCAG
CAAGGAGCAGCAGTTTCGCATCTCCACCCAGAATCTGGTGCTGATCCAGAAGTATCAGGTGAACGAGCTGGGCAAGGAGATCAGG
CCATGTCGGCTGAAGAAGCGCCCACCCGTGCGGAGCGGAGGATCCGGAGGATCTGGAGGCAGCACCAACCTGTCTGACATCATCG
AGAAGGAGACAGGCAAGCAGCTGGTCATCCAGGAGAGCATCCTGATGCTGCCCGAAGAAGTCGAAGAAGTGATCGGAAACAAGC
CTGAGAGCGATATCCTGGTCCATACCGCCTACGACGAGAGTACCGACGAAAATGTGATGCTGCTGACATCCGACGCCCCAGAGTAT
AAGCCCTGGGCTCTGGTCATCCAGGATTCCAACGGAGAGAACAAAATCAAAATGCTGTCTGGCGGCTCAAAAAGAACCGCCGACG
GCAGCGAATTCGAGCCCAAGAAGAAGAGGAAAGTC

Nme2-rAPOBEC1-i8: BPSV40-NLS, Nme2Cas9, rAPOBEC1, UGI, Linkers

AAACGGACAGCCGACGGAAGCGAGTTCGAGTCACCAAAGAAGAAGCGGAAAGTCGAAGATATGGCCGCCTTCAAGCCTAACCCA
ATCAATTACATCCTGGGACTGGCCATCGGAATCGCATCCGTGGGATGGGCTATGGTGGAGATCGACGAGGAGGAGAATCCTATCC
GGCTGATCGATCTGGGCGTGAGAGTGTTTGAGAGGGCCGAGGTGCCAAAGACCGGCGATTCTCTGGCTATGGCCCGGAGACTGG
CACGGAGCGTGAGGCGCCTGACACGGAGAAGGGCACACAGGCTGCTGAGGGCACGCCGGCTGCTGAAGAGAGAGGGCGTGCTG
CAGGCAGCAGACTTCGATGAGAATGGCCTGATCAAGAGCCTGCCAAACACCCCCTGGCAGCTGAGAGCAGCCGCCCTGGACAGGA
AGCTGACACCACTGGAGTGGTCTGCCGTGCTGCTGCACCTGATCAAGCACCGCGGCTACCTGAGCCAGCGGAAGAACGAGGGAG
AGACAGCAGACAAGGAGCTGGGCGCCCTGCTGAAGGGAGTGGCCAACAATGCCCACGCCCTGCAGACCGGCGATTTCAGGACAC
CTGCCGAGCTGGCCCTGAATAAGTTTGAGAAGGAGTCCGGCCACATCAGAAACCAGAGGGGCGACTATAGCCACACCTTCTCCCG
CAAGGATCTGCAGGCCGAGCTGATCCTGCTGTTCGAGAAGCAGAAGGAGTTTGGCAATCCACACGTGAGCGGAGGCCTGAAGGA
GGGAATCGAGACCCTGCTGATGACACAGAGGCCTGCCCTGTCCGGCGACGCAGTGCAGAAGATGCTGGGACACTGCACCTTCGAG
CCTGCAGAGCCAAAGGCCGCCAAGAACACCTACACAGCCGAGCGGTTTATCTGGCTGACAAAGCTGAACAATCTGAGAATCCTGG
AGCAGGGATCCGAGAGGCCACTGACCGACACAGAGAGGGCCACCCTGATGGATGAGCCTTACCGGAAGTCTAAGCTGACATATG
CCCAGGCCAGAAAGCTGCTGGGCCTGGAGGACACCGCCTTCTTTAAGGGCCTGAGATACGGCAAGGATAATGCCGAGGCCTCCAC
ACTGATGGAGATGAAGGCCTATCACGCCATCTCTCGCGCCCTGGAGAAGGAGGGCCTGAAGGACAAGAAGTCCCCCCTGAACCTG
AGCTCCGAGCTGCAGGATGAGATCGGCACCGCCTTCTCTCTGTTTAAGACCGACGAGGATATCACAGGCCGCCTGAAGGACAGGG
TGCAGCCTGAGATCCTGGAGGCCCTGCTGAAGCACATCTCTTTCGATAAGTTTGTGCAGATCAGCCTGAAGGCCCTGAGAAGGATC
GTGCCACTGATGGAGCAGGGCAAGCGGTACGACGAGGCCTGCGCCGAGATCTACGGCGATCACTATGGCAAGAAGAACACAGAG
GAGAAGATCTATCTGCCCCCTATCCCTGCCGACGAGATCAGAAATCCTGTGGTGCTGAGGGCCCTGTCCCAGGCAAGAAAAGTGAT
CAACGGAGTGGTGCGCCGGTACGGATCTCCAGCCCGGATCCACATCGAGACCGCCAGAGAAGTGGGCAAGAGCTTCAAGGACCG
GAAGGAGATCGAGAAGAGACAGGAGGAGAATCGCAAGGATCGGGAGAAGGCCGCCGCCAAGTTTAGGGAGTACTTCCCTAACTT
TGTGGGCGAGCCAAAGTCTAAGGACATCCTGAAGCTGCGCCTGTACGAGCAGCAGCACGGCAAGTGTCTGTATAGCGGCAAGGA
GATCAATCTGGTGCGGCTGAACGAGAAGGGCTATGTGGAGATCGATCACGCCCTGCCTTTCTCCAGAACCTGGGACGATTCTTTTA
ACAATAAGGTGCTGGTGCTGGGCAGCGAGAACCAGAATAAGGGCAATCAGACACCATACGAGTATTTCAATGGCAAGGACAACTC
CAGGGAGTGGCAGGAGTTCAAGGCCCGCGTGGAGACCTCTAGATTTCCCAGGAGCAAGAAGCAGCGGATCCTGCTGCAGAAGTT
CGACGAGGATGGCTTTAAGGAGTGCAACCTGAATGACACCAGATACGTGAACCGGTTCCTGTGCCAGTTTGTGGCCGATCACATCC
TGCTGACCGGCAAGGGCAAGAGAAGGGTGTTCGCCTCTAATGGCCAGATCACAAACCTGCTGAGGGGATTTTGGGGACTGAGGA
AGGTGCGGGCAGAGAATGACAGACACCACGCACTGGATGCAGTGGTGGTGGCATGCAGCACCGTGGCAATGCAGCAGAAGATCA
CAAGATTCGTGAGGTATAAGGAGATGAACGCCTTTGACGGCAAGACCATCGATAAGGAGACAGGCAAGGTGCTGCACCAGAAGA
CCCACTTCCCCCAGCCTTGGGAGTTCTTTGCCCAGGAAGTGATGATCCGGGTGTTCGGCAAGCCAGACGGCAAGCCTGGCGGATCA
GGAGGCTCTGGCGGTTCAGGTGGATCAGGCGGTAGCGGAGGTTCAGGTGGTAGCTCAGAGACTGGCCCAGTGGCTGTGGACCCC
ACATTGAGACGGCGGATCGAGCCCCATGAGTTTGAGGTATTCTTCGATCCGAGAGAGCTCCGCAAGGAGACCTGCCTGCTTTACGA
AATTAATTGGGGGGGCCGGCACTCCATTTGGCGACATACATCACAGAACACTAACAAGCACGTCGAAGTCAACTTCATCGAGAAGT
TCACGACAGAAAGATATTTCTGTCCGAACACAAGGTGCAGCATTACCTGGTTTCTCAGCTGGAGCCCATGCGGCGAATGTAGTAGG
GCCATCACTGAATTCCTGTCAAGGTATCCCCACGTCACTCTGTTTATTTACATCGCAAGGCTGTACCACCACGCTGACCCCCGCAATC
GACAAGGCCTGCGGGATTTGATCTCTTCAGGTGTGACTATCCAAATTATGACTGAGCAGGAGTCAGGATACTGCTGGAGAAACTTT
GTGAATTATAGCCCGAGTAATGAAGCCCACTGGCCTAGGTATCCCCATCTGTGGGTACGACTGTACGTTCTTGAACTGTACTGCATC
ATACTGGGCCTGCCTCCTTGTCTCAACATTCTGAGAAGGAAGCAGCCACAGCTGACATTCTTTACCATCGCTCTTCAGTCTTGTCATT
ACCAGCGACTGCCCCCACACATTCTCTGGGCCACCGGGTTGAAAGGCTCCTCTGGCTCTGAGACACCTGGCACAAGCGAGAGCGCA
ACACCTGAAAGCAGCGGCGAGTTTGAGGAGGCCGATACCCCAGAGAAGCTGAGGACACTGCTGGCAGAGAAGCTGTCTAGCAGG
CCAGAGGCAGTGCACGAGTACGTGACCCCACTGTTCGTGTCCAGGGCACCCAATCGGAAGATGTCTGGCGCCCACAAGGACACAC
TGAGAAGCGCCAAGAGGTTTGTGAAGCACAACGAGAAGATCTCCGTGAAGAGAGTGTGGCTGACCGAGATCAAGCTGGCCGATC
TGGAGAACATGGTGAATTACAAGAACGGCAGGGAGATCGAGCTGTATGAGGCCCTGAAGGCAAGGCTGGAGGCCTACGGAGGA
AATGCCAAGCAGGCCTTCGACCCAAAGGATAACCCCTTTTATAAGAAGGGAGGACAGCTGGTGAAGGCCGTGCGGGTGGAGAAG
ACCCAGGAGAGCGGCGTGCTGCTGAATAAGAAGAACGCCTACACAATCGCCGACAATGGCGATATGGTGAGAGTGGACGTGTTCT
GTAAGGTGGATAAGAAGGGCAAGAATCAGTACTTTATCGTGCCTATCTATGCCTGGCAGGTGGCCGAGAACATCCTGCCAGACAT
CGATTGCAAGGGCTACAGAATCGACGATAGCTATACATTCTGTTTTTCCCTGCACAAGTATGACCTGATCGCCTTCCAGAAGGATGA
GAAGTCCAAGGTGGAGTTTGCCTACTATATCAATTGCGACTCCTCTAACGGCAGGTTCTACCTGGCCTGGCACGATAAGGGCAGCA
AGGAGCAGCAGTTTCGCATCTCCACCCAGAATCTGGTGCTGATCCAGAAGTATCAGGTGAACGAGCTGGGCAAGGAGATCAGGCC
ATGTCGGCTGAAGAAGCGCCCACCCGTGCGGAGCGGAGGATCCGGAGGATCTGGAGGCAGCACCAACCTGTCTGACATCATCGA
GAAGGAGACAGGCAAGCAGCTGGTCATCCAGGAGAGCATCCTGATGCTGCCCGAAGAAGTCGAAGAAGTGATCGGAAACAAGCC
TGAGAGCGATATCCTGGTCCATACCGCCTACGACGAGAGTACCGACGAAAATGTGATGCTGCTGACATCCGACGCCCCAGAGTATA
AGCCCTGGGCTCTGGTCATCCAGGATTCCAACGGAGAGAACAAAATCAAAATGCTGTCTGGCGGCTCAAAAAGAACCGCCGACGG
CAGCGAATTCGAGCCCAAGAAGAAGAGGAAAGTC
