# Supplementary material for: Domain-inlaid Nme2Cas9 adenine base editors with improved activity and targeting scope
Source: Nat Commun. 2024 Feb 17;15:1458. doi: 10.1038/s41467-024-45763-5 (PMC10874451; doi:10.1038/s41467-024-45763-5)
Supplement: Supplementary file 2 — Reporting Summary [file 41467_2024_45763_MOESM2_ESM.pdf]

nature portfolio

Corresponding author(s): Erik J. Sontheimer
Last updated by author(s): 1/26/2023

Reporting Summary

Nature Portfolio wishes to improve the reproducibility of the work that we publish. This form provides structure for consistency and transparency in reporting. For further information on Nature Portfolio policies, see our Editorial Policies and the Editorial Policy Checklist.

Statistics

For all statistical analyses, confirm that the following items are present in the figure legend, table legend, main text, or Methods section.

n/a	Confirmed	
☐	☒	The exact sample size (*n*) for each experimental group/condition, given as a discrete number and unit of measurement
☐	☒	A statement on whether measurements were taken from distinct samples or whether the same sample was measured repeatedly
☐	☒	The statistical test(s) used AND whether they are one- or two-sided<br>*Only common tests should be described solely by name; describe more complex techniques in the Methods section.*
☒	☐	A description of all covariates tested
☐	☒	A description of any assumptions or corrections, such as tests of normality and adjustment for multiple comparisons
☐	☒	A full description of the statistical parameters including central tendency (e.g. means) or other basic estimates (e.g. regression coefficient) AND variation (e.g. standard deviation) or associated estimates of uncertainty (e.g. confidence intervals)
☐	☒	For null hypothesis testing, the test statistic (e.g. *F*, *t*, *r*) with confidence intervals, effect sizes, degrees of freedom and *P* value noted<br>*Give P values as exact values whenever suitable.*
☒	☐	For Bayesian analysis, information on the choice of priors and Markov chain Monte Carlo settings
☒	☐	For hierarchical and complex designs, identification of the appropriate level for tests and full reporting of outcomes
☒	☐	Estimates of effect sizes (e.g. Cohen's *d*, Pearson's *r*), indicating how they were calculated

Our web collection on statistics for biologists contains articles on many of the points above.

Software and code

Policy information about availability of computer code

Data collection	FACS data was collected from a MACSQuant VYB from Miltenyi Biotec. NGS amplicon deep sequencing data were generated using a Illumina MiniSeq or NextSeq 2000 systems. The western blot data was acquired using a Bio-Rad ChemiDoc MP Imaging System.
Data analysis	Microsoft® Excel® for Microsoft 365 MSO (Version 2302 Build 16.0.16130.20298); GraphPad Prism 9.4.0; CRISPResso 2.0.40; FlowJo 10.8.1

For manuscripts utilizing custom algorithms or software that are central to the research but not yet described in published literature, software must be made available to editors and reviewers. We strongly encourage code deposition in a community repository (e.g. GitHub). See the Nature Portfolio guidelines for submitting code & software for further information.

Data

Policy information about availability of data

All manuscripts must include a data availability statement. This statement should provide the following information, where applicable:

- Accession codes, unique identifiers, or web links for publicly available datasets
- A description of any restrictions on data availability
- For clinical datasets or third party data, please ensure that the statement adheres to our policy

Sequencing data that support the findings of this study will be available in the NCBI SRA (bioproject #PRJNA1033663) upon publication. Source data for all figures and associated data are provided within the Source Data file. Plasmids described in this paper will be made available from Addgene.

Human research participants

Policy information about studies involving human research participants and Sex and Gender in Research.

Reporting on sex and gender	n/a
Population characteristics	n/a
Recruitment	n/a
Ethics oversight	n/a

Note that full information on the approval of the study protocol must also be provided in the manuscript.

Field-specific reporting

Please select the one below that is the best fit for your research. If you are not sure, read the appropriate sections before making your selection.

☒ Life sciences ☐ Behavioural & social sciences ☐ Ecological, evolutionary & environmental sciences

For a reference copy of the document with all sections, see nature.com/documents/nr-reporting-summary-flat.pdf

Life sciences study design

All studies must disclose on these points even when the disclosure is negative.

Sample size	No statistical analyses were done to predetermine sample size for the mouse studies, however the sample size selected correlates with those generally used in the field to develop and validate genome editing technologies. All studies conducted in mammalian cells, had an n = 3 biological replicates performed independently from one another. The selected sample sizes for mammalian cell experiments were chosen as it also matches standards used within the genome editing field.
Data exclusions	Two samples were omitted due to poor read alignment of the sequencing reads:<br>Figure 2b-c: "Lsp1_CT_Nme2_Smu_i1_R3"<br>Figure 3c: "Rosa26_ON_Nme2-smu-i8_r1".
Replication	Three biological replicates were performed independently from one another. Replication attempts were successful as evidenced by two to three positive results.
Randomization	Mouse samples were randomly assigned for either control or treatment groups. No covariates were controlled for in these studies.
Blinding	Samples were prepared unblinded with identical conditions between treated groups. Experimentalist performing tests, also prepared the samples and reagents needed.

Reporting for specific materials, systems and methods

We require information from authors about some types of materials, experimental systems and methods used in many studies. Here, indicate whether each material, system or method listed is relevant to your study. If you are not sure if a list item applies to your research, read the appropriate section before selecting a response.

Materials & experimental systems

n/a	Involved in the study	
☐	☒	Antibodies
☐	☒	Eukaryotic cell lines
☒	☐	Palaeontology and archaeology
☐	☒	Animals and other organisms
☒	☐	Clinical data
☒	☐	Dual use research of concern

Methods

n/a	Involved in the study	
☒	☐	ChIP-seq
☐	☒	Flow cytometry
☒	☐	MRI-based neuroimaging

Antibodies

Antibodies used	Mouse-anti 6x-His (ThermoFisher #MA1-21315, dilution 1:2000); Rabbit-anti-LaminB1 (Abcam #AB16048, dilution, 1:10,000); Goat-anti-mouse (LI-COR IRDye®800CW, dilution 1:20,000); Goat-anti-rabbit (LI-COR IRDye®680RD, dilution 1:20,000)
Validation	Mouse-anti 6x-His (ThermoFisher #MA1-21315), has been independently validated by multiple groups for western blot in addition to verification by the manufacturer. This Antibody was verified by ThermoFisher with relative expression to ensure that the antibody binds to the antigen stated. Additionally, Thermo Fisher Scientific has adopted three concepts to help improve experimental reproducibility and reporting based on the recommendations of the International Working Group for Antibody Validation see also the manufacturers reference page. https://www.thermofisher.com/antibody/product/6x-His-Tag-Antibody-clone-HIS-H8-Monoclonal/MA1-21315<br><br>Rabbit-anti-LaminB1 (Abcam #AB16048), has been independently validated by multiple groups for western blot in addition to the manufacturer, by use of a knockout cell line. Abcam is leading the way in addressing this with our range of recombinant monoclonal antibodies and knockout edited cell lines for gold-standard validation. See also manufacturers reference page. https://www.abcam.com/products/primary-antibodies/lamin-b1-antibody-nuclear-envelope-marker-ab16048.html<br><br>Goat-anti-mouse IRDye®800CW (LI-COR #925-32210) and goat-anti-rabbit IRDye®680RD (LI-COR #926-68071) were validated by the manufacturer in addition to our group in previous publications (e.g., https://doi.org/10.1038/s41467-021-26518-y).

Eukaryotic cell lines

Policy information about cell lines and Sex and Gender in Research

Cell line source(s)	Cell line: HEK293T; Source: ATCC (ATCC® CRL-3216™)<br>Cell line: Neuro2a; Source: ATCC (ATCC® CCL-131™)<br>Cell line: ABE mCherry reporter HEK293T; Source (Dr. Scot Wolfe - Univ. Massachusetts Medical School; as described in previous manuscripts (https://doi.org/10.1038/s41467-021-22295-w) and (https://doi.org/10.1089/genbio.2022.0015)<br>Cell line: Rett - Patient Derivied Fibroblasts (PDF) cell with c.502 C>T, p.R168X mutation, derived from female patients.<br>Cell line: Rett - Patient Derivied Fibroblasts (PDF) cell with c.916 C>T, p.R306C mutation, derived from female patients.<br>Rett-PDF cells were obtained as a gift from the Rett Syndrome Research Trust (RSRT) repository. https://reverserett.org/research/initiatives/clinical-initiatives/#stack-20200723-233013
Authentication	Not authenticated.
Mycoplasma contamination	Not tested.
Commonly misidentified lines (See ICLAC register)	No commonly misidentified cell line was used.

Animals and other research organisms

Policy information about studies involving animals; ARRIVE guidelines recommended for reporting animal research, and Sex and Gender in Research

Laboratory animals	C57Bl/6 mice were purchased from the Jackson Laboratory (Stock No. 000664) and housed in the University of Massachusetts Medical School animal facility. Facility and living conditions consisted of 65-75°F (~18-23°C) ambient temperature, 40-60% humidity and a 14-hour light/10-hour dark cycle. Both male and female adult mice (ages: 8 to 15 weeks old) were used. Three mice per group were used for the animal studies.
Wild animals	The study did not involve wild animals.
Reporting on sex	Sex was not considered in this study. The selected sample sizes for mouse experiments were chosen as it matches standards used within the genome editing field for editor validation. references. Zhang et al., Gen Biotech 2022. Davis et al., Nat. BME 2022.
Field-collected samples	The study did not involve samples collected in the field.
Ethics oversight	All animal study procedures were approved by the Institutional Animal Care and Use Committee (IACUC) at the University of Massachusetts Medical School.

Note that full information on the approval of the study protocol must also be provided in the manuscript.

Flow Cytometry

Plots

Confirm that:

☒ The axis labels state the marker and fluorochrome used (e.g. CD4-FITC).

☒ The axis scales are clearly visible. Include numbers along axes only for bottom left plot of group (a 'group' is an analysis of identical markers).

☒ All plots are contour plots with outliers or pseudocolor plots.

☒ A numerical value for number of cells or percentage (with statistics) is provided.

Methodology

Sample preparation	Cells were transected and collected according to the Flow cytometry section in the methods.
Instrument	MACSQuant® VYB - Miltenyi Biotec
Software	Flowjo V10.8.1
Cell population abundance	Cell population abundance for the live and singlet gates were similar across the biological replicates and constructs tested. Based on the ABE editing plasmid construct used, mCherry+ efficiency ranged from 0-70%.
Gating strategy	Cells were selected for the live population based on forward and side scatter. Live cells were selected for singlets with forward scatter height and area. mCherry-positive cells were gated to measure overall ABE editing activity, with the gating strategy based on unedited cells.

☒ Tick this box to confirm that a figure exemplifying the gating strategy is provided in the Supplementary Information.
