# Supplementary material for: Domain-inlaid Nme2Cas9 adenine base editors with improved activity and targeting scope
Source: Nat Commun. 2024 Feb 17;15:1458. doi: 10.1038/s41467-024-45763-5 (PMC10874451; doi:10.1038/s41467-024-45763-5)
Supplement: Supplementary file 3 — Description of Additional Supplementary Files [file 41467_2024_45763_MOESM3_ESM.pdf]

Title: Supplementary Data 1.
Description: Oligonucleotides. Sequences of target sites and oligonucleotides used in this study Sheet I: NGS Target sites and spacers used in this manuscript. Column headings include: site ID, protospacer sequence (5' - 3'), Cas9 ortholog, reference and, oligonucleotide sequences for target site amplification. Sheet II: Information of members within the guide-target library. Sequences for library member features included in column headers.
